# Synthesis and Evaluation of 5-Heteroarylidene-2-thioxo-4-thiazolidinones as PIM1 Kinase Inhibitors

**DOI:** 10.3390/molecules31193413

**Published:** 2026-09-25

**Authors:** Kelly Malzewski, Alexander N. Erickson, William A. Donaldson

**Affiliations:** Department of Chemistry, Marquette University, P.O. Box 1881, Milwaukee, WI 53201-1881, USA; kelly.malzewski@marquette.edu (K.M.); alexander.erickson@marquette.edu (A.N.E.)

**Keywords:** nitrogen heterocycles, PIM1 kinase, single-crystal X-ray diffraction

## Abstract

The PIM kinases belong to the family of serine/threonine kinases which are involved in a wide range of cellular processes through multiple pathways that can lead to cell proliferation, apoptosis inhibition, migration, survival, and transcription. As such, inhibitors of PIM kinases are of pharmaceutical interest for their potential antitumor activity. A family of five known and seventeen new 5-heteroarylidene-2-thioxo-4-thiazolidinones were prepared through the Knoevenagel condensation of rhodanine with heteroarylaldehydes; the structure of **5i** was elucidated by single-crystal X-ray diffraction analysis. The synthesized compounds were evaluated for the inhibition of PIM1 kinase. Two of the previously unknown inhibitors, **5p** and **5u** (IC_50_ = 2.60 ± 0.29 nM and 9.01 ± 0.67 nM respectively), were further evaluated for selectivity within the PIM family of kinases as well as against a select group of six other kinases.

## 1. Introduction

The Proviral integration site for Moloney Murine Leukemia virus (PIM) kinases belong to the family of serine/threonine kinases consisting of three isoforms: PIM1, PIM2, PIM3 [1]. PIM kinases are involved in a wide range of cellular processes through multiple pathways that can lead to cell proliferation, apoptosis inhibition, migration, survival, transcription, and energy metabolism [2]. PIM1 is mainly expressed in bone marrow, spleen and thymus, and is highly expressed in prostate tumor cell lines and hematopoietic lymphocytes [3]. PIM2 expression is detectable in lymphoid and brain tissues and predominates in solid form tumors within the liver, prostate and hematologic malignancies [4]. PIM3 is primarily distributed in breast, kidney and brain. Overexpression of PIM is related with poor outcomes in multiple types of cancers [5], and thus inhibitors of PIM kinases present an attractive avenue for therapeutic development.

Three small-molecule PIM inhibitors, SGI-1776 [6], AZD1208 [7,8], and PIM447 (LGH-447) [9] (Figure 1), have undergone clinical trials for various prostate, ovarian, triple-negative breast and multiple hematologic cancers [1]. Although some promising results were observed for SGI-1776 (NCT01239108 and NCT00848601) and AZD1208 (NCT01489722 and NCT 01588548) both were eventually canceled due to cardiac toxicity for SGI-1176 and elevated CYP3A4 activity for AZD1208 leading to more rapid clearance. Phase I clinical studies of PIM447 with relapsed/refractory multiple myeloma patients (NCT02160951) were eventually canceled as disease progression was observed in 54 of 79 patients. There are currently no PIM kinase inhibitor therapeutics approved by the FDA.

PIM kinases share the bilobal-fold structure typical of the serine/threonine family of kinases, consisting of a N-terminal lobe, a C-terminal lobe and a hinge region linking these two parts in which ATP binds [2]. The N-terminal region contains antiparallel β-sheets, an αC-helix and an activation loop rich in glycine that binds ATP. The C-terminal region contains catalytic residues responsible for transferring phosphate. Within the N-terminal lobe, the activation loop (A-loop) contains an acidic (Asp) residue, creating a salt bridge with receptors in the C-terminal (catalytic loop) that results in continuous activation, proving the absence of the regulatory domain. A unique feature is the presence of a proline residue within the hinge region. All three PIM isoforms share a high amino-acid sequence homology with a 61% similarity between PIM1 and PIM2, a 71% similarity between PIM1 and PIM3 and a 44% similarity between PIM2 and PIM3 [10].

While small-molecule compounds containing the rhodanine scaffold have been portrayed as “frequent hitters” or assay-interfering compounds [11], others consider these as “privileged scaffolds” in drug discovery [12]. In a study of 163 rhodanines, hydantoins, thiohydantoins and thiazolidinediones, Mendgen et al. [13], concluded that “rhodanines and related scaffolds should not be regarded as problematic or promiscuous binders per se.” They further suggested that “particularly strong requirements, i.e., affinity in the lower nanomolar range and proven selectivity for the target, are applied in the further assessment of rhodanines and related compounds.”

A high-throughput screen, conducted by Davies, Russell and co-workers [14], of ~20,000 drug-like compounds revealed that rhodanine containing structures possessed inhibitory activity against PIM1 and PIM2. They observed that 5-arylidene-2-thioxo-4-thiazolidinones exhibited IC_50_ values in the 36 to 974 nM range. Computational docking (PDB 2C3I) suggested further optimization of 5-(2-phenylthiazo-4-yl)methylene-2-thioxo-4-thiazolidinone. These studies led to several compounds with low single digit nanomolar potency (Figure 2). While there was little selectivity between the PIM kinase isoforms, the *p*-CF_3_ analog (**1d**) exhibited good selectivity against a series of select 18 other kinases. Notably, **1d** demonstrated considerably activity for GSK-β.

Subsequently, Sawaguchi et al., reported that 5-[(3′-ethoxy-4′-hydroxyphenyl)methylene]-2-thioxo-4-thiazolidinone **2a** (Figure 3) inhibits PIM1 kinase with an IC_50_ = 420 nM [15]. Molecular docking of compound **2a** (PDB 3F2A) predicted hydrogen bonding interactions between the NH and the carbonyl group with Lys-67 and Asp-168 while the 3-ethoxy-4-hydroyphenyl group showed interactions with Asp-128 and Glu-171. Further optimization in this series afforded **2b**. Inhibition of compound **2b** increased significantly in dose-dependent manners with IC_50_ = 16 nM for PIM1. When assayed against PC-3, A549, HCT-116 and MV-4-11 cancer cell lines, **2b** exhibited IC_50_ values of 0.26, 0.13, 0.16, and 0.15 μM in only 10% serum conditions.

Our laboratory had previously studied the synthesis and evaluation of fourteen 5-(heteroarylmethylene)hydantoins as glycogen synthase kinase-3β (GSK-3β) inhibitors (Figure 4) [16]. Although subsequent reevaluation of **3a**-**c** in a kinase HotSpot^TM^ assay [17] at 25 μM revealed similar percent activities for GSK-3β and PIM1 inhibition, these hydantoin fragment-containing compounds were considerably less potent compared to the rhodamine-based compounds **1a**-**1d** or **2a**-**b**.

Inspired by the PIM1 kinase inhibitory activity of compounds **1a**-**d** and **2a**-**b**, we prepared a series of 5-(heteroarylmethylene)-2-thioxo-4-thiazolidinones **5a-v** and examined their inhibitory activity against PIM1.

## 2. Results and Discussion

### 2.1. Chemistry

Five known (**5a**, **5b**, **5h**, **5j**, and **5k**) and seventeen new 5-heteroarylidene-2-thioxo-4-thiazolidinones (**5c**-**g**, **5i,** and **5l**-**u**) were prepared through the Knoevenagel condensation of rhodanine with heteroarylaldehydes (Scheme 1). In addition, two known 5-arylidene-2-thioxo-4-thiazolidinones (**5v-w**) were prepared in a similar fashion. In all cases, the product precipitated from the reaction mixture during heating and was further precipitated at the end of heating by dilution with deionized water. The ^1^H NMR spectra of the crude product from two of the reactions (**5a** and **5w**), prepared using reaction conditions A, indicated the presence of unreacted rhodanine. These products were purified by recrystallization from ethanol/water. Under alternative reaction conditions B (NaOAc/HOAc) all reactions proceeded to completion.

While there are two possible stereoisomers (*Z*- and *E*-) about the C5–C6 olefinic bond only a single stereoisomer was detected, as evidenced by only a single set of signals in their ^1^H NMR spectra in d_6_-DMSO. Koettner et al., have recently reported on the preparation of several pure (Z)-5-arylidene-2-thioxothiazolidin-4-ones and the photochemical interconversion to a photostationary state (pss) mixture of (*Z*)- and (*E*)-stereoisomers [18]. These studies revealed that the (*Z*)-stereoisomers are more thermodynamically stable than their corresponding (*E*)-stereoisomers, typically by >1.9 kcal/mol. In addition, ^1^H NMR spectra of the pss mixtures indicated that the signal for H6 of the (*Z*)-stereoisomer appears shifted downfield of the corresponding signal for the (*E*)-stereoisomer [18]. This is presumably due to the anisotropic effect of the carbonyl functionality of the rhodanine fragment.

Products **5a**-**b**, **5h, 5j**-**k**, and **5v-w** were identified by comparison of their spectral data with values in the literature [13,14,19,20]. The structural assignments for 2-thioxo-4-thiazolidinones **5d**-**f** and **5l**-**u** are based on their ^1^H NMR and ^13^C NMR spectral data. In particular, singlets in their ^1^H NMR spectra in the range ca. δ 7.45–7.70 ppm and signals in their ^13^C NMR spectra in the range δ 200–202 and 169 ppm correspond to H6, C=S, and C=O respectively. Due to the known thermodynamic stability of the (Z)-stereoisomer compared to the (*E*)-stereoisomer (vide supra), all of the products were all assigned the (*Z*)-stereochemistry.

The ^13^C NMR spectra of (*Z*)-5-[(1*H*-imidazol-2-yl)methylene]- and (*Z*)-5-[(1*H*-benzimidazol-2-yl)methylene]-2-thioxo-4-thiazolidinones (**5b** and **5c**) exhibit sharp signals for C2 (C=S), C4 (C=O), C5, C6 and C2′ of the imidazole-2-yl or benzimidazol-2-yl moiety. However, the ^13^C NMR signals for C4′/C5′ of the imidazole-2-yl moiety and the aryl ring carbons of the benzimidazol-2-yl moiety appear as broadened signals; the broadening is more pronounced in more concentrated d_6_-DMSO solution. This broadening can be attributed to tautomerism due to proton transfer between the two nitrogen atoms of the imidazol-2-yl/benzimazol-2-yl nitrogens. This type of signal broadening has been previously observed for a variety of 2-substituted imidazoles and benzimidazoles [21].

The structural assignment for **5i** was corroborated by single-crystal X-ray diffraction analysis (Figure 5). The 2-thioxo-4-thiazolidinone, thiazole and phenyl rings of **5i** are nearly all co-planar in the crystal structure, similar to that observed for other 5-heteroarylidene-2-thioxo-4-thiazolidinones [18].

### 2.2. Biological Evaluation

#### 2.2.1. Inhibition of PIM1

The ability of sixteen of the 5-heteroarylidene-2-thioxo-4-thiazolidinones and two 5-arylidene-2-thioxo-4-thiazolidinones **5w**-**x** to inhibit PIM1 at 25 μM was measured in a kinase HotSpot^TM^ assay with 10 μM ATP; measurements of remaining percent activities were made in duplicate and values are reported as the average (Table 1). All compounds exhibited some inhibitory active at 25 μM, with remaining activity in the range 30.7% (**5b**) to 0.005% (**5k**). Compounds **5c**, **5d**, **5f**-**i**, **5k**, and **5m**-**w** were further assessed in 10-point inhibition measurement conducted in triplicate (Table 1). The PIM1 IC_50_ inhibitory activity of compounds **5h** and **5k** have previously been reported by Bataille et al. [14] and the values measured in the present studies using the HotSpot^TM^ assay (a ^33^P-labeled ATP displacement assay) agree reasonably well with the literature values (obtained using an enzyme coupled spectrophotometric assay). 5-(*N*-Methyl-3-indolylmethylene)-2-thioxo-4-thiazolidinone (**5a**) and the 5-(imidazolylmethylene)-2-thioxo-4-thiazolidinones (**5b**-**f**) exhibited modest PIM1 inhibitory activity. The thiazolyl analogs **5g** and **5i** (IC_50_ = 144 and 24.2 nM respectively) were 3 to 9 times more potent compared to their imidazolyl counterparts **5c** and **5f** (IC_50_ = 479 and 238 nM respectively). Known 2-phenylthiazo-4-yl compound **5h** [14], is greater than one order of magnitude more potent than the isomeric 2-phenylthiazo-5-yl compound **5i**.

Within the 5-[(pyridinyl)methylene]-2-thioxo-4-thiazolidinone series (**5j**-**u**) the presence of either a methyl, bromo, or methoxy substituent on the pyridinyl ring increased inhibitory activity compared to the unsubstituted parent compound **5j**. The 5- and 6-bromo-, 6-phenyl- and 6-methoxy-2-pyridinyl analogs **5j**, **5k**, **5l** and **5g** exhibit the greatest increase in potency with IC_50_ values in the single to low double-digit nanomolar range (IC_50_ = 16.1 ± 1.3 nM, 10.8 ± 1.6 nM, 9.01 ± 0.67 nM and 2.60 ± 0.29 nM respectively). The 5-methoxy-2-pyridinyl (**5o**) and 6-methoxy-3-pyridinyl compounds (**5q**) were significantly less potent (IC_50_ = 144 ± 9 nM and 115 ± 4 nM respectively) compared to isomeric **5p**. Finally, the 4-bromophenyl (**5v**) and 3-methoxyphenyl analog (**5w**) were less potent (IC_50_ = 20.3 ± 0.9 and 33.7 ± 1.5 nM respectively) compared to the pyridine analogs **5s** and **5p**, thus drawing attention to the superiority of this heterocycle over a phenyl ring.

#### 2.2.2. Selectivity for PIM Isoform and Selected Other Kinases

Since the kinase selectivity of the most potent PIM1 inhibitor (**5h**) was previously evaluated by Bataille et al. [14], the next two most potent inhibitors (**5p** and **5u**) were selected from the 5-[(pyridinylene]-2-thioxo-4-thiazolidinone series for further evaluation. The percent residual activity of the three isoforms (PIM1, PIM2 and PIM3) as well as a representative series of six kinases was determined at a single concentration (Figure 6, Table 2). Both **5p** and **5u** exhibited comparable potency to inhibit the PIM kinase family. Compound **5u** showed a high level of selectivity against DYRK2, EGFR, ERK2/MAPK1, GSK-3β and IGF-1R (defined as >20% inhibition); however, **5u** was somewhat potent as an inhibitor for CK2a. In comparison, compound **5p** was less selective, exhibiting meaningful inhibitory activity for CK2a, DYRK2 and GSK-3β.

#### 2.2.3. Molecular Docking

Dockings of **5a-u** were calculated using the Mcule online one-click program which uses the Vina docking algorithm (https://mcule.com). Vina uses a sophisticated gradient optimization method in its local optimizing procedure, and which was designed to improve speed of the execution and accuracy [22]. The authors of the program describe the scoring function as ‘more of a “machine learning” than directly physics-based in its nature.’ The protein structure used for docking (PDB 3F2A) was that obtained from co-crystals of PIM1 with (*E*)-3-{3-[6-(4-methyl [1,4]diazepan-1-yl)pyrazine-2-yl]phenyl}acrylic acid (DAPPA) [23] in which the acrylic acid functionality binds in the deep front pocket near the Lys67-Asp186 salt bridge. For compounds **5b**-**e**, **5l**-**t**, and **5v**-**w** at least one of the calculated docking poses has the ligand oriented such that the carbonyl group and N–H of the 2-thioxo-4-thiazoldinone is in proximity to the salt bridge (Table 1) as was also reported by Sawaguchi et al. for 5-arylidene-2-thioxo-4-thiazolidinones **2a** and **2b** [15]. In addition, these docking poses also evidenced pi-stacking between Phe89 (Phe18 in Mcule) and the heteroaryl group of each. This is best exemplified in the docking picture for **5p** (Figure 7a). In contrast, for **5h** and **5u** the top calculated poses oriented this moiety toward the outside of the ATP binding cleft, with hydrogen bonding between the carbonyl group and the N–H of the 2-thioxo-4-thiazoldinone are hydrogen bonded with the backbone N–H of Asp128 and the carboxylate of Asp131 (Figure 7b). In contrast, the docking computations for **5i**-**k** did not exhibit any interactions other than van der Waals contacts. For this reason, the predictive value of these computations may be limited.

## 3. Materials and Methods

### 3.1. Chemistry

#### 3.1.1. General Experimental

Melting points were measured in open capillary tubes on a MelTemp melting point apparatus and are uncorrected. Infrared spectra were recorded on a Nicolet iS50 FT-IR (Madison, WI, USA) instrument using ATR mode. NMR spectra were recorded on either a Varian UnityInova 400 MHz or a Varian VNMRS 600 MHz instrument (Palo Alto, CA, USA). d_6_-DMSO was purchased from Cambridge Isotope Laboratories (Tewksbury, MA, USA). ^1^H NMR were calibrated to 2.49 ppm for d_5_-DMSO present in the d_6_-DMSO solvent and ^13^C NMR spectra were calibrated from the central peak at 39.52 ppm for d_6_-DMSO. Coupling constants are reported in Hz. High-resolution mass spectra were obtained from the COSMIC lab at Old Dominion University using a Bruker SolariX FTICR-MS spectrometer (Bremen, Germany). Copies of the NMR and HRMS spectra can be found in the Supplementary Materials.

X-ray diffraction investigation of **5i** was conducted using on a Bruker D8 Venture 4-circle diffractometer (Madison, WI, USA) coupled to a Photon III C14 CMOS area detector (Karlsruhe, Germany) with Cu *K_α_* radiation (λ = 1.54184 Å) provided by a Incoatec IµS 3.0 micro-focus Cu X-ray source (Geesthacht, Germany) running at 50.0 kV and 1.1 mA. All data manipulations were carried out using the Bruker APEX-6 Software Suite [24]. Data were reduced, correcting for absorption, using the SADABS program [25,26]. Space group assignment was done using XPREP within the SHELXTL suite of programs [23]. Using the ShelXle [27,28] graphical interface, the structure was solved by dual space methods employed by ShelXT [29] and refined by full-matrix least-squares methods against F^2^ using ShelXL [30] with scattering factors and anomalous dispersion terms taken from the literature [31]. All non-hydrogen atoms not apparent from the initial solution were found from difference Fourier maps, and all heavy atoms were refined anisotropically. All hydrogen atoms were found from difference Fourier maps and allowed to refine freely. Copies of the crystal and refinement data for **5i** can be found in the Supplementary Materials.

Aldehydes **4a-k** and **4t-s** were purchased from Ambeed, Inc. (Buffalo Grove, IL, USA); aldehydes **4l, 4p and 4r** were purchased from Combi-Blocks (San Diego, CA, USA); aldehyde **4o** was purchased from Asta Tech (Bristol, PA, USA); aldehyde **4q** was purchased from Accella ChemBio Product List (San Diego, CA, USA); rhodamine was purchased from Sigma-Aldrich (St. Louis, MO, USA).

#### 3.1.2. General Procedure for Preparation of 5-(Heteroarylmethylene)-2-thioxo-4-thiazolidinones

Procedure A. To a solution of rhodanine (5 mmol) in 0.4 M NaOH (10 mL) was added a solution of the carboxaldehyde (5 mmol) dissolved in ethanol (7.5 mL). The solution was stirred at room temperature for 10 min and then glacial acetic acid (3 mL) was added. Typically solids began to precipitate from the mixture within 30 s. The mixture was heated to 60 °C for 1 h. After cooling to room temperature, the precipitate was collected via vacuum filtration, washed several times with H_2_O, and dried in air.

Procedure B. Rhodanine (5 mmol) and sodium acetate (5 mmol) were dissolved in glacial acetic acid (15 mL) with warming to 70 °C. The heteroaryl carboxaldehyde (5 mmol) was added to the mixture and the mixture heated to 100 °C for 5 to 7.5 h. After cooling to room temperature over a period of 8 h, the precipitate was collected via vacuum filtration, washed several times with H_2_O, and dried in air.

(*Z*)-5-[(*N*-Methylindol-3-yl)methylene]-2-thioxo-4-thiazolidinone (**5a**). The reaction of *N*-methylindole-3-carboxaldehyde (796 mg, 5.00 mmol) with rhodanine (665 mg, 4.99 mmol) was carried out by general procedure A. The crude product appeared to contain some unreacted rhodanine. For this reason, recrystallization of the crude product from ethanol/water gave **5a** as a brick-red solid (654 mg, 48%). A pure sample was recrystallized from ethanol/water; mp > 215 °C; ^1^H NMR (d_6_-acetone, 600 MHz) δ 7.99 (d, J = 1H, 7.8 Hz), 7.95 (s, 1H), 7.77 (s, 1H), 7.57 (d, 1H, J = 7.8 Hz), 7.37 (t, 1H, J = 7.5 Hz), 7.31 (t, 1H, J = 7.5 Hz), 4.05 (s, 3H, N–CH_3_); ^1^H NMR (d_6_-DMSO, 600 MHz) δ 7.95 (d, 1H, J = 7.8 Hz), 7.89 (s, 1H), 7.88 (s, 1H), 7.57 (d, 1H, J = 8.4 Hz), 7.33 (t, 1H, J = 7.8 Hz), 7.26 (t, 1H, J = 7.5 Hz), 3.94 (s, 3H, N–CH_3_); ^13^C NMR (d_6_-DMSO, 100 MHz) δ 194.9 (C=S), 169.6 (C=O), 137.0, 133.4, 127.3, 123.9, 123.4, 121.7, 118.6, 111.0, 110.0, 33.4 ppm; IR (atr): 1751, 1723, 1671, 1609, 1225, 1189 cm^–1^. The ^1^H NMR chemical shifts (d_6_-acetone) for this compound are consistent with the literature values [13].

(*Z*)-5-[(1*H*-Imidazol-2-yl)methylene]-2-thioxo-4-thiazolidinone (**5b**). The reaction of 1*H*-imidazole-2-carboxaldehyde (439 mg, 4.57 mmol) with rhodanine (607 mg, 4.56 mmol) was carried out by general procedure B to give **5b** as a deep burgundy-colored solid (640 mg, 67%); mp > 225 °C; ^1^H NMR (d_6_-DMSO, 600 MHz) δ 13.50 (br s, 1H), 12.90 (s, 1H), 7.49 (brs, 1H), 7.35 (br s, 1H), 7.32 (s, 1H); ^13^C NMR (d_6_-DMSO, 100 MHz) δ 199.8 (C=S), 169.2 (C=O), 142.1, 132.5 (br), 125.7, 121.1 (br), 116.7 ppm; IR (atr): 1681, 1602, 1542, 1423, 1394, 1321, 1310, 1239, 1196, 1154, 1100, 1071, 1017, 732, 668 cm^–1^. The NMR spectral data for this compound is consistent with the literature values [18].

(*Z*)-5-[(1*H*-Benzoimidazol-2-yl)methylene]-2-thioxo-4-thiazolidinone (**5c**). The reaction of 1*H*-benzoimidazole-2-carboxaldehyde (454 mg, 3.11 mmol) with rhodanine (422 mg, 3.17 mmol) was carried out by general procedure B to give **5c** as a deep burgundy-colored solid (576 mg, 68%); mp > 225 °C; ^1^H NMR (d_6_-DMSO, 600 MHz) δ 13.09 (br s, 1H), 7.76 (br s, 1H), 7.61 (br s, 1H), 7.45 (s, 1H, H-6), 7.30 (br s, 2H); ^13^C NMR (d_6_-DMSO, 100 MHz) δ 199.9 (C=S), 169.0 (C=O), 147.1, 131.8, 124.3 (br), 123.3 (br), 119.8 (br), 116.3, 112.4 (br) ppm; IR (atr): 3307, 1706, 1623, 1430, 1208, 1176, 739 cm^–1^. HRMS *m*/*z* 262.0103 (calcd for C_11_H_7_N_3_S_2_O+H^+^ 262.0103).

(*Z*)-5-[(Imidazol-4-yl)methylene]-2-sulfanylidene-1,3-thiazolidin-4-one (**5d**). The reaction of 4-imidazolecarboxaldehyde (574 mg, 5.97 mmol) with rhodanine (790 mg, 5.93 mmol) was carried out by general procedure B to give **5d** as a mustard-yellow solid (1.14 g, 91%); mp > 225 °C; ^1^H NMR (d_6_-DMSO, 600 MHz) δ 13.50 (br s, 1H), 12.70 (br s, 1H), 7.95 (s, 1H), 7.83 (s, 1H), 7.54 (s, 1H, H-6); ^13^C NMR (d_6_-DMSO, 100 MHz) δ 199.8 (C=S), 168.4 (C=O), 138.5, 135.7, 124.5, 124.1, 121.3 ppm; IR (atr): 1675, 1605, 1258, 1222, 1196 cm^–1^. HRMS *m*/*z* 211.9946 (calcd for C_7_H_5_N_3_S_2_O+H^+^ 211.9947).

(*Z*)-5-[(2-Methyl-1*H*-Imidazol-4-yl)methylene]-2-thioxo-4-thiazolidinone (**5e**). The reaction of 2-methyl-1*H*-imidazole-4-carboxaldehyde (538 mg, 4.89 mmol) with rhodanine (400 mg, 4.88 mmol) was carried out by general procedure B to give **5e** as a mustard-colored solid (561 mg, 51%); mp > 225 °C; ^1^H NMR (d_6_-DMSO, 600 MHz) δ 13.50 (br s, 1H), 12.40 (br s, 1H), 7.72 (s, 1H, H5′), 7.45 (s, 1H, H-6), 2.34 (s, CH_3_); ^13^C NMR (d_6_-DMSO, 100 MHz) δ 199.8 (C=S), 169.5 (C=O), 147.3, 135.4, 125.0, 124.3, 120.5, 14.0 ppm; IR (atr): 1690, 1597, 1575, 1549, 1426, 1305, 1222, 1190, 674, 551 cm^–1^. HRMS *m*/*z* 226.0104 (calcd for C_8_H_7_N_3_S_2_O+H^+^ 226.0103).

(*Z*)-5-[(2-Phenyl-1*H*-Imidazol-4-yl)methylene]-2-thioxo-4-thiazolidinone (**5f**). The reaction of 2-phenyl-1*H*-imidazole-4-carboxaldehyde (254 mg, 1.47 mmol) with rhodanine (192 mg, 1.44 mmol) was carried out by general procedure B to give **5f** as a brick-orange solid (291 mg, 71%); mp > 225 °C; ^1^H NMR (d_6_-DMSO, 600 MHz) δ 13.43 (br s, 1H), 13.28 (s, 1H), 8.02 (d, 2H, J = 7.8 Hz), 7.98 (s, 1H, H5′), 7.55 (s, 1H, H-6), 7.53 (t, 2H, J = 7.8 Hz), 7.44 (t, 1H, J = 7.5 Hz); ^13^C NMR (d_6_-DMSO, 100 MHz) δ 200.0 (C=S), 169.4 (C=O), 148.1, 136.7, 129.3, 129.2, 129.0, 126.1, 125.4, 123.6, 122.0 ppm; IR (atr): 3253, 1675, 1583, 1544, 1456, 1234, 1203, 685 cm^–1^. HRMS *m*/*z* 288.0159 (calcd for C_13_H_9_N_3_SO+H^+^ 288.0260).

(*Z*)-5-[(1,3-Benzothiazo-2-yl)methylene]-2-thioxo-4-thiazolidinone (**5g**). The reaction of 1,3-benzothiazole-2-carboxaldehyde (638 mg, 3.91 mmol) with rhodanine (518 mg, 3.97 mmol) was carried out by general procedure B to give **5g** as a mocha-colored solid (0.849 g, 78%); mp > 225 °C; ^1^H NMR (d_6_-DMSO, 600 MHz) δ 8.22 (d, 1H, J = 8.4 Hz), 8.17 (d, 1H, J = 8.4 H), 7.94 (s, 1H, H-6), 7.61 (t, 1H, J = 8.4 Hz), 7.54 (t, 1H, J = 8.4 Hz); ^13^C NMR (d_6_-DMSO, 100 MHz) δ 199.8 (C=S), 168.8 (C=O), 161.3, 153.0, 136.0, 132.9, 127.4, 126.5, 123.5, 122.8, 119.7 ppm; IR (atr): 1690, 1595, 1587, 1427, 1220, 753, 727 cm^–1^; HRMS *m*/*z* 278.9714 (calcd for C_11_H_6_N_2_S_3_O+H^+^ 278.9715).

(*Z*)-5-[(2′-Phenylthiazo-4-yl)methylene]-2-thioxo-4-thiazolidinone (**5h**). The reaction of 2-phenylthiazole-4-carboxaldehyde (776 mg, 4.10 mmol) with rhodanine (548 mg, 4.11 mmol) was carried out by general procedure B to give **5h** as a lemon yellow-colored solid (1.116 g, 89%); mp > 225 °C; ^1^H NMR (d_6_-DMSO, 600 MHz) δ 8.39 (s, 1H, H5′), 8.03 (d, 2H, J = 7.2 H), 7.65 (s, 1H, H-6), 7.62–7.55 (m, 3H); ^13^C NMR (d_6_-DMSO, 100 MHz) δ 168.2 (C=O), 150.5, 132.1, 131.2, 129.7, 127.4, 126.5, 122.3 ppm, signal for C=S not observed; IR (atr): 1711, 1606, 1440, 1198, 1139 cm^–1^. The ^1^H NMR spectral data for this compound is consistent with the literature spectral data [14].

(*Z*)-5-[(2′-Phenylthiazo-5-yl)methylene]-2-thioxo-4-thiazolidinone (**5i**). The reaction of 2-phenylthiazole-5-carboxaldehyde (966 mg, 5.05 mmol) with rhodanine (673 mg, 5.05 mmol) was carried out by general procedure B to give **5i** as an orange-red-colored solid (803 mg, 52%); mp > 225 °C; ^1^H NMR (d_6_-DMSO, 600 MHz) δ 8.46 (s, 1H, H4′), 8.10–8.04 (m, 2H), 7.98 (s, 1H), 7.60–7.53 (m, 3H); ^13^C NMR (d_6_-DMSO, 100 MHz) δ 194.2 (C=S), 172.0, 168.9 (C=O), 150.3, 133.3, 132.2, 131.5, 129.5, 127.1, 126.6, 121.5 ppm; IR (atr): 1727, 1687, 1586, 1246, 1208, 1193, 1152, 755, 675, 630 cm^–1^; HRMS *m*/*z* 304.9870 (calcd for C_13_H_8_N_2_S_3_O+H^+^ 304.9872). Crystals of **5i** for X-ray diffraction analysis were deposited from a solution of the material dissolved in d_6_-DMSO in a 5mm NMR tube at ambient temperature. Crystal Data: C_15_H_8_D_6_N_2_O_2_S_4_, (C_13_H_8_N_2_OS_3_, C_2_D_6_OS), M_r_ = 388.56, Monoclinic, *C*2/*c*, (No. 15, *a* = 38.572(3) Å, *b* = 5.1661(4) Å, *c* = 17.2662(11) Å, α = 90°, β = 102.583(3)°, γ = 90°, V = 3357.9(4) Å^3^), Z = 8, T = 100(2) K, λ = 1.54184 Å (Cu *K_α_*), μ = 5.287 mm^−1^, d_calc_ = 1.537 g/cm^3^, 42176 reflections collected; 3635 unique (R_int_ = 0.0979), giving R_1_ = 0.0318, wR_2_ = 0.0844 for 3111 data with [I>2σ(I)] and R_1_ = 0.0462, wR_2_ = 0.0898 for all 3635 data. CCDC 2574955 contains the supplementary crystallographic data for this paper. These data can be obtained free of charge from the Cambridge Crystallographic Data Centre, 12 Union Road, Cambridge CB2 1EZ, UK.

(*Z*)-5-[(2′-Pyridinyl)methylene]-2-thioxo-4-thiazolidinone (**5j**). The reaction of pyridine-2-carboxaldehyde (0.48 mL, 0.55 g, 5.0 mmol) with rhodanine (667 mg, 5.00 mmol) was carried out by general procedure A to give **5j** as a green-yellow solid (862 mg, 76%); mp > 215 °C; ^1^H NMR (d_6_-DMSO, 600 MHz) δ 8.78 (d, 1H, J = 4.2 Hz, H6′), 7.95 (t, 1H, J = 7.5 Hz, H4′), 7.90 (d, 1H, J = 7.8 Hz, H3′), 7.67 (s, 1H, H-6), 7.43 (dd, 1H, J = 4.8, 6.0 Hz, H5′); ^13^C NMR (d_6_-DMSO, 100 MHz) δ 202.1 (C=S), 169.4 (C=O), 151.1, 149.6, 137.7, 129.8, 128.3, 127.6, 124.1 ppm; IR (atr): 3048, 1704, 1580, 1431, 1228, 1183 cm^–1^. The ^1^H NMR spectral data is consistent with the literature values [17].

(*Z*)-5-[(Quinolin-2-yl)methylene]-2-thioxo-4-thiazolidinone (**5k**). The reaction of quinoline-2-carboxaldehyde (995 mg, 6.33 mmol) with rhodanine (802 mg, 6.02 mmol) was carried out by general procedure B to give **5k** as a olive-brown-colored solid (1.06 g, 65%); mp 260 °C (dec.); ^1^H NMR (d_6_-DMSO, 600 MHz) δ 13.70 (br s, NH), 8.49 (d, 1H, J = 8.5 Hz), 8.20 (dd, 1H, J = 0.8, 8.0 Hz), 7.97 (d, 1H, J = 8.5 Hz), 7.84 (ddd, 1H, J = 1.4, 6.9, 8.5 Hz), 7.68 (ddd, 1H, J = 1.1, 6.9, 8.0 Hz); ^13^C NMR (d_6_-DMSO, 100 MHz) δ 202.5 (C=S), 169.3 (C=O), 151.6, 147.1, 137.4, 131.5, 130.8, 128.4, 128.1, 127.9, 126.9, 126.8, 124.6 ppm. The NMR spectral data for this compound is consistent with the literature spectral data [14].

(*Z*)-5-[(4′-Methyl-2′-pyridinyl)methylene]-2-thioxo-4-thiazolidinone (**5l**). The reaction of 4-methylpyridine-2-carboxaldehyde (0.56 mL, 5.04 mmol) with rhodanine (667 mg, 5.01 mmol) was carried out by general procedure A to give **5l** as a yellow solid (914 mg, 77%); mp > 215 °C; ^1^H NMR (d_6_-DMSO, 600 MHz) δ 8.63 (d, 1H, *J* = 4.8 Hz, H6′), 7.73 (s, 1H, H3′), 7.60 (s, 1H, H-6), 7.27 (d, 1H, J = 4.2 Hz, H5′), 2.36 (s, 3H, CH_3_); ^13^C NMR (d_6_-DMSO, 100 MHz) δ 202.1 (C=S), 169.3 (C=O), 150.9, 149.2, 148.5, 129.7, 128.9, 127.6, 124.8, 20.4 (CH_3_) ppm; IR (atr): 1712, 1611, 1595, 1442, 1248, 1201 cm^–1^. HRMS *m*/*z* 258.9969 (calcd for C_10_H_8_N_2_S_2_O+Na^+^ 258.9970).

(*Z*)-5-[(5′-Methyl-2′-pyridinyl)methylene]-2-thioxo-4-thiazolidinone (**5m**). The reaction of 5-methylpyridine-2-carboxaldehyde (608 mg, 5.00 mmol) with rhodanine (668 mg, 5.02 mmol) was carried out by general procedure A to give **5m** as a yellow solid (965 mg, 82%); mp > 215 °C; ^1^H NMR (d_6_-DMSO, 600 MHz) δ 8.62 (s, H6′), 7.79–7.73 (m, 2H, H3′ & H4′), 7.62 (s, 1H, H-6), 2.35 (s, 3H, CH_3_); ^13^C NMR (d_6_-DMSO, 100 MHz) δ 202.0 (C=S), 169.6 (C=O), 150.0. 148.5, 137.7, 134.2, 128.7, 127.7, 127.6, 18.1 (CH_3_) ppm; IR (atr): 1713, 1612, 1549, 1443, 1234, 1188 cm^–1^. HRMS *m*/*z* 258.9969 (calcd for C_10_H_8_N_2_S_2_O+Na^+^ 258.9970).

(*Z*)-5-[(6′-Methyl-2′-pyridinyl)methylene]-2-thioxo-4-thiazolidinone (**5n**). The reaction of 6-methylpyridine-2-carboxaldehyde (614 mg, 5.07 mmol) with rhodanine (667 mg, 5.07 mmol) was carried out by general procedure A to give **5n** as a golden yellow solid (883 mg, 74%); mp > 215 °C; ^1^H NMR (d_6_-DMSO, 600 MHz) δ 7.83 (t, 1H, J = 7.5 Hz, H4′), 7.70 (d, 1H, J = 7.2 Hz, H3′), 7.64 (s, 1H, H-6), 7.30 (d, 1H, J = 7.8 Hz, H5′), 2.58 (s, 3H, CH_3_); ^13^C NMR (d_6_-DMSO, 100 MHz) δ 202.5 (C=S), 169.4 (C=O), 158.3, 150.3, 137.9, 129.5, 127.7, 125.5, 123.7, 23.7 (CH_3_) ppm; IR (atr): 1700, 1609, 1576, 1433, 1225, 1190 cm^–1^; HRMS *m*/*z* 258.9969 (calcd for C_10_H_8_N_2_S_2_O+Na^+^ 258.9970).

(*Z*)-5-[(5′-Methoxy-2′-pyridinyl)methylene]-2-thioxo-4-thiazolidinone (**5o**). The reaction of 5-methoxypyridine-2-carboxaldehyde (353 mg, 2.62 mmol) with rhodanine (344 mg, 2.58 mmol) was carried out by general procedure B to give **5o** as a greenish-yellow solid (509 mg, 78%); mp > 225 °C; ^1^H NMR (d_6_-DMSO, 600 MHz) δ 13.6 (br s, 1H, NH), 8.51 (s, 1H, H6′), 7.87 (d, 1H, J = 8.7 Hz), 7.63 (s, 1H, H-6), 7.53 (d, 1H, J = 8.7 Hz), 3.90 (s, 3H, OCH_3_); ^13^C NMR (d_6_-DMSO, 100 MHz) δ 201.7 (C=S), 169.4 (C=O), 155.7, 143.6, 138.2, 129.4, 127.7, 126.8, 121.0, 56.1 (OCH_3_) ppm; IR (atr): 1695, 1580, 1549, 1427, 1402, 1299, 1229, 1184 cm^–1^; HRMS *m*/*z* 273.0100 (calcd for C_10_H_8_N_2_S_2_O_2_+H^+^ 273.0100).

(*Z*)-5-[(6′-Methoxy-2′-pyridinyl)methylene]-2-thioxo-4-thiazolidinone (**5p**). The reaction of 6-methoxypyridine-2-carboxaldehyde (0.61 mL, 5.7 mmol) with rhodanine (665 mg, 4.99 mmol) was carried out by general procedure A to give **5p** as an orange-yellow solid (946 mg, 75%); mp > 215 °C; ^1^H NMR (d_6_-DMSO, 600 MHz) δ 7.82 (t, 1H, J = 7.5 Hz, H4′), 7.56 (s, 1H, H-6), 7.48 (d, 1H, J = 7.2 Hz), 6.88 (d, 1H, J = 7.8 Hz), 4.01 (s, 3H, OCH_3_); ^13^C NMR (d_6_-DMSO, 100 MHz) δ 201.0 (C=S), 169.8 (C=O), 164.0, 149.2, 140.7, 129.3, 128.3, 122.7, 112.7, 55.0 (OCH_3_) ppm; IR (atr): 3140, 1690, 1576, 1468, 1447, 1413, 1347, 1283, 1266, 1220, 1187, 1152, 1072 cm^–1^; HRMS *m*/*z* 274.9918 (calcd for C_10_H_8_N_2_S_2_O_2_+Na^+^ 274.9919).

(*Z*)-5-[(6′-Methoxy-3′-pyridinyl)methylene]-2-thioxo-4-thiazolidinone (**5q**). The reaction of 6-methoxypyridine-3-carboxaldehyde (356 mg, 2.62 mmol) with rhodanine (349 mg, 2.62 mmol) was carried out by general procedure B to give **5q** as a yellow solid (283 mg, 43%); mp > 225 °C; ^1^H NMR (d_6_-DMSO, 600 MHz) δ 8.50 (s, 1H, H2′), 7.86 (d, 1H, J = 8.7 Hz), 7.60 (s, 1H, H-6), 6.99 (d, 1H, J = 8.7 Hz), 3.92 (s, 3H, OCH_3_); ^13^C NMR (d_6_-DMSO, 100 MHz) δ 195.4 (C=S), 169.9 (C=O), 164.4, 151.1, 139.2, 128.4, 124.5, 123.9, 111.7, 53.9 (OCH_3_) ppm; IR (atr): 1690, 1592, 1498, 1442, 1385, 1315, 1306, 1200, 1141 cm^–1^. HRMS *m*/*z* 253.0099 (calcd for C_10_H_8_N_2_S_2_O_2_+H^+^ 253.0100).

(*Z*)-5-[(4′-Bromo-2′-pyridinyl)methylene]-2-thioxo-4-thiazolidinone (**5r**). The reaction of 4-bromopyridine-2-carboxaldehyde (745 mg, 4.01 mmol) with rhodanine (533 mg, 4.00 mmol) was carried out by general procedure A to give **5r** as a fluffy yellow solid (863 mg, 72%); mp > 215 °C; ^1^H NMR (d_6_-DMSO, 600 MHz) δ 8.65 (d, 1H, J = 5.4 Hz, H6′), 8.23 (d, 1H, J = 1.2 Hz, H3′), 7.71 (dd, 1H, J = 1.8, 4.8 Hz, H5′), 7.64 (s, 1H, H-6); ^13^C NMR (d_6_-DMSO, 100 MHz) δ 201.6 (C=S), 169.3 (C=O), 152.8, 150.5, 133.2, 131.6, 130.6, 126.7, 126.0 ppm; IR (atr): 1725, 1613, 1563, 1438, 1228, 1187, 831 cm^–1^. HRMS *m*/*z* 300.9098 (calcd for C_9_H_5_N_2_S_2_OBr+H^+^ 300.9099).

(*Z*)-5-[(5′-Bromo-2′-pyridinyl)methylene]-2-thioxo-4-thiazolidinone (**5s**). The reaction of 5-bromopyridine-2-carboxaldehyde (931 mg, 5.01 mmol) with rhodanine (675 mg, 5.00 mmol) was carried out by general procedure A to give **5s** as a yellow solid (1.20 g, 80%); mp > 215 °C; ^1^H NMR (d_6_-DMSO, 400 MHz) δ 8.91 (s, 1H, H6′), 8.21 (d, 1H, J = 8.01 Hz), 7.86 (d, 1H, J = 8.4 Hz), 7.66 (s, 1H, H-6); ^13^C NMR (d_6_-DMSO, 100 MHz) δ 201.4 (C=S), 169.5 (C=O), 150.4, 150.0, 140.2, 130.6, 129.3, 126.3, 120.7 ppm; IR (atr): 3035, 1709, 1611, 1443, 1228, 1186, 918, 835, 758, 525 cm^–1^. HRMS *m*/*z* 322.8918 (calcd for C_9_H_5_N_2_S_2_OBr+Na^+^ 322.8919).

(*Z*)-5-[(6′-Bromo-2′-pyridinyl)methylene]-2-thioxo-4-thiazolidinone (**5t**). The reaction of 6-bromopyridine-2-carboxaldehyde (931 mg, 5.01 mmol) with rhodanine (667 mg, 5.00 mmol) was carried out by general procedure A to give **5t** as a golden-yellow solid (1.17 g, 78%); mp > 215 °C; ^1^H NMR (d_6_-DMSO, 600 MHz) δ 7.91 (d, 1H, J = 7.2 Hz), 7.87 (t, 1H, J = 7.8 Hz, H4′), 7.66 (d, 1H, J = 7.8 Hz), 7.63 (s, 1H, H-6); ^13^C NMR (d_6_-DMSO, 100 MHz) δ 201.3 (C=S), 169.5 (C=O), 152.4, 140.9, 140.8, 131.5, 128.4, 127.3, 125.8 ppm; IR (atr): 1692, 1602, 1567, 1543, 1443, 1231, 1192, 1156, 1127, 790 cm^–1^. HRMS *m*/*z* 322.8918 (calcd for C_9_H_5_N_2_S_2_OBr+Na^+^ 322.8919).

(*Z*)-5-[(6′-Phenyl-2-pyridinyl)methylene]-2-thioxo-4-thiazolidinone (**5u**). The reaction of 6-phenylpyridine-2-carboxaldehyde (250 mg, 1.36 mmol) with rhodanine (186 mg, 1.40 mmol) was carried out by general procedure B to give **5u** as an olive-yellow solid (359 mg, 89%); mp > 225 °C; ^1^H NMR (d_6_-DMSO, 600 MHz) δ 8.16 (d, 2H, J = 7.2 Hz), 8.03 (t, 1H, J = 7.8 Hz), 8.00 (d, 1H, J = 7.8 Hz), 7.86 (d, 1H, J = 7.2 Hz), 7.72 (s, 1H, H-6), 7.59 (t, 2H, J = 7.8 Hz), 7.52 (t, 1H, J = 7.5 Hz, H4′); ^13^C NMR (d_6_-DMSO, 100 MHz) δ 201.4 (C=S), 169.6 (C=O), 156.9, 150.9, 138.7, 137.9, 129.8, 129.5, 129.0, 127.7, 127.1, 127.0, 121.0 ppm; IR (atr): 1718, 1700, 1614, 1571, 1439, 1223, 1181, 757, 680, 522 cm^–1^. HRMS *m*/*z* 299.0307 (calcd for C_15_H_10_N_2_S_2_O+H^+^ 299.0307).

(*Z*)-5-[(4′-Bromophenyl)methylene]-2-thioxo-4-thiazolidinone (**5v**). The reaction of 4-bromobenzaldehyde (697 mg, 3.77 mmol) with rhodanine (500 mg, 3.75 mmol) was carried out by general procedure B to give **5v** as a yellow-colored solid (912 mg, 81%); mp >215 °C; ^1^H NMR (d_6_-DMSO, 400 MHz) δ 7.70 (d, 2H, J = 8.4 Hz), 7.58 (s, 1H, H-6), 7.50 (d, 2H, J = 8.4 Hz) ppm. The NMR spectral data is consistent with the literature values [17]. IR (atr): 1701, 1593, 1437, 1398, 1219, 824, 800, 744, 514 cm^–1^.

(*Z*)-5-[(3′-Methoxyphenyl)methylene]-2-thioxo-4-thiazolidinone (**5w**). The reaction of 3-methoxybenzaldehyde (620 mg, 4.55 mmol) with rhodanine (669 mg, 5.02 mmol) was carried out by general procedure B to give **5w** as a golden-yellow solid (835 mg, 73%). A pure sample was recrystallized from ethanol/water; mp > 215 °C; ^1^H NMR (d_6_-DMSO, 600 MHz) δ 7.60 (s, 1H, H-6), 7.45 (t, 1H, J = 8.4 Hz, H5′), 7.16–7.13 (m, 2H), 7.07 (dd, 1H, J = 1.8, 8.4 Hz), 3.80 (s, 3H, OCH_3_); ^13^C NMR (d_6_-DMSO, 100 MHz) δ 195.8 (C=S), 169.5 (C=O), 159.8, 134.4, 131.7, 130.7, 126.0, 122.5, 116.8, 115.6, 55.4 ppm; IR (atr): 3020, 1695, 1594, 1572, 1431, 1270, 1214, 912, 764, 671 cm^–1^. The NMR spectral data is consistent with the literature values [17].

### 3.2. Biological Evaluation

#### Inhibition of PIM1 Kinase

In vitro inhibition assays of **5a**-**w** were performed by Reaction Biology (Malvern, PA, USA) using their HotSpot^TM^ assay as previously described [32]. The following description is similar to that in the reference. Briefly, the PIM1 kinase and each compound along with required cofactors were prepared in reaction buffer (20 mM Hepes pH 7.5, 10 mM MgCl_2_, 1 mM EGTA, 0.01% Brij35, 0.02 mg/mL BSA, 0.1 mM Na_3_VO_4_, 2 mM DTT, 1% DMSO). Compounds were delivered into the reaction. After 20 min, a mixture of ATP and ^33^P ATP to a final concentration of 10 μM. After reaction for 120 min at 25 °C, the mixture was spotted onto P81 ion exchange filter paper. Unbound phosphate was removed by extensive washing with 0.75% phosphoric acid. Background derived from control reactions with inactive enzyme were subtracted and the kinase activity expressed as percent remaining kinase activity compared to vehicle. Percent inhibition at a single concentration were conducted in duplicate at 25 μM inhibitor; values are reported as the average. IC_50_ determinations were conducted in triplicate; and curve fits were obtained using Prism. Values are reported as the average with calculated standard deviation. Staurosporine was tested in a 10-dose IC_50_ 4-fold serial dilution as a control; this gave an IC_50_ value of 1.3 nM.

## 4. Conclusions

5-Heteroarylidene-2-thioxo-4-thiazolidinones are readily prepared from rhodanine and heteroaryl aldehydes via a Knoevenagel condensation. All of the compounds were assigned to be Z-stereoisomers. While all of the 2-thioxo-4-thiazolidinones exhibited some level of PIM1 inhibitory activity, the 2-pyridinyl-substituted series were generally superior to the indol-3-yl-, imidazolyl and benzoimidazolyl- series. Only the known 2-phenylthiazo-4-yl- (**5h**) [14], and new 6-methoxy-2-pyridinyl- (**5p**) and 6-phenyl-2-pyridinyl-2-thioxo-4-thiazolidinones (**5u**) exhibited IC_50_ values in the single-digit nanomolar level. Compounds **5p** and **5u** also exhibited similar potent inhibitory activity against the isoforms PIM2 and PIM3 and were selective as inhibitors for these kinases compared to EGFR, EGR2/MAPK1, and IGF-1R kinases. In view of the mixed selectivity results, further refinement of this scaffold is imperative.

## Figures and Tables

**Figure 1:**
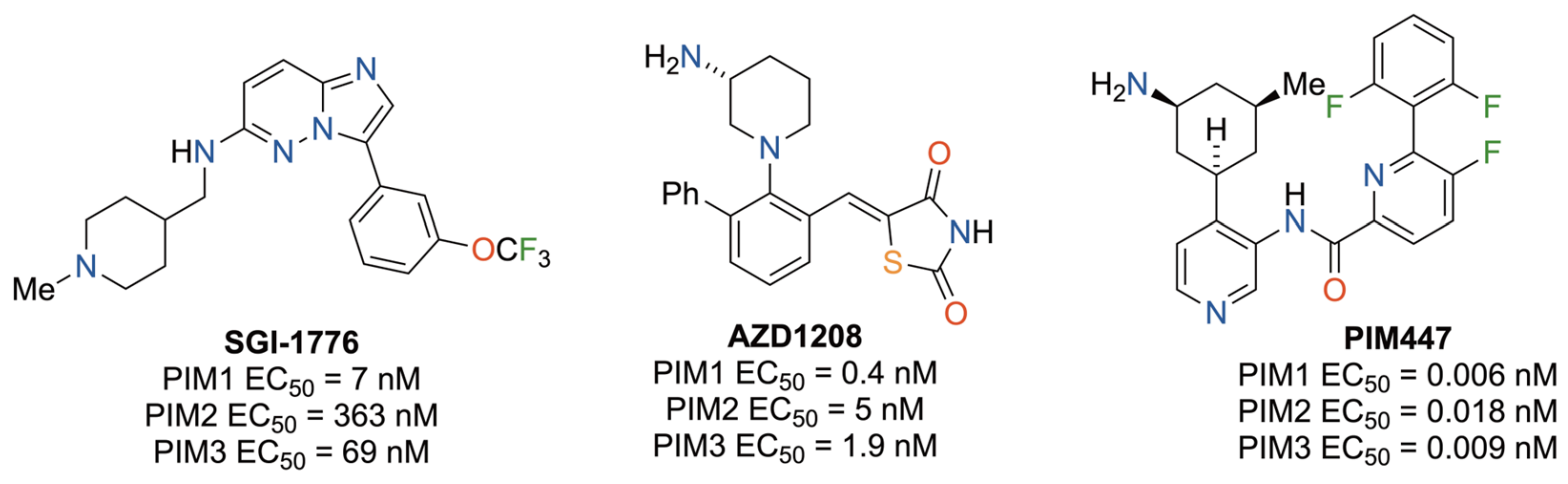
PIM kinase inhibitors which have undergone clinical trials.

**Figure 2:**
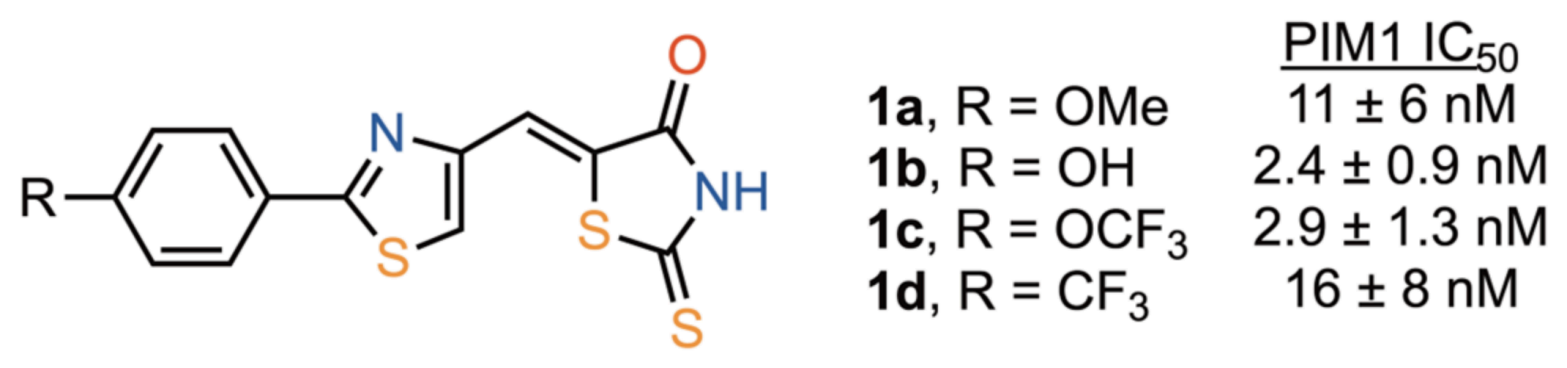
5-(2-Phenylthiazo-4-yl)methylene-2-thioxo-4-thiazolidinone PIM kinase inhibitors [14].

**Figure 3:**
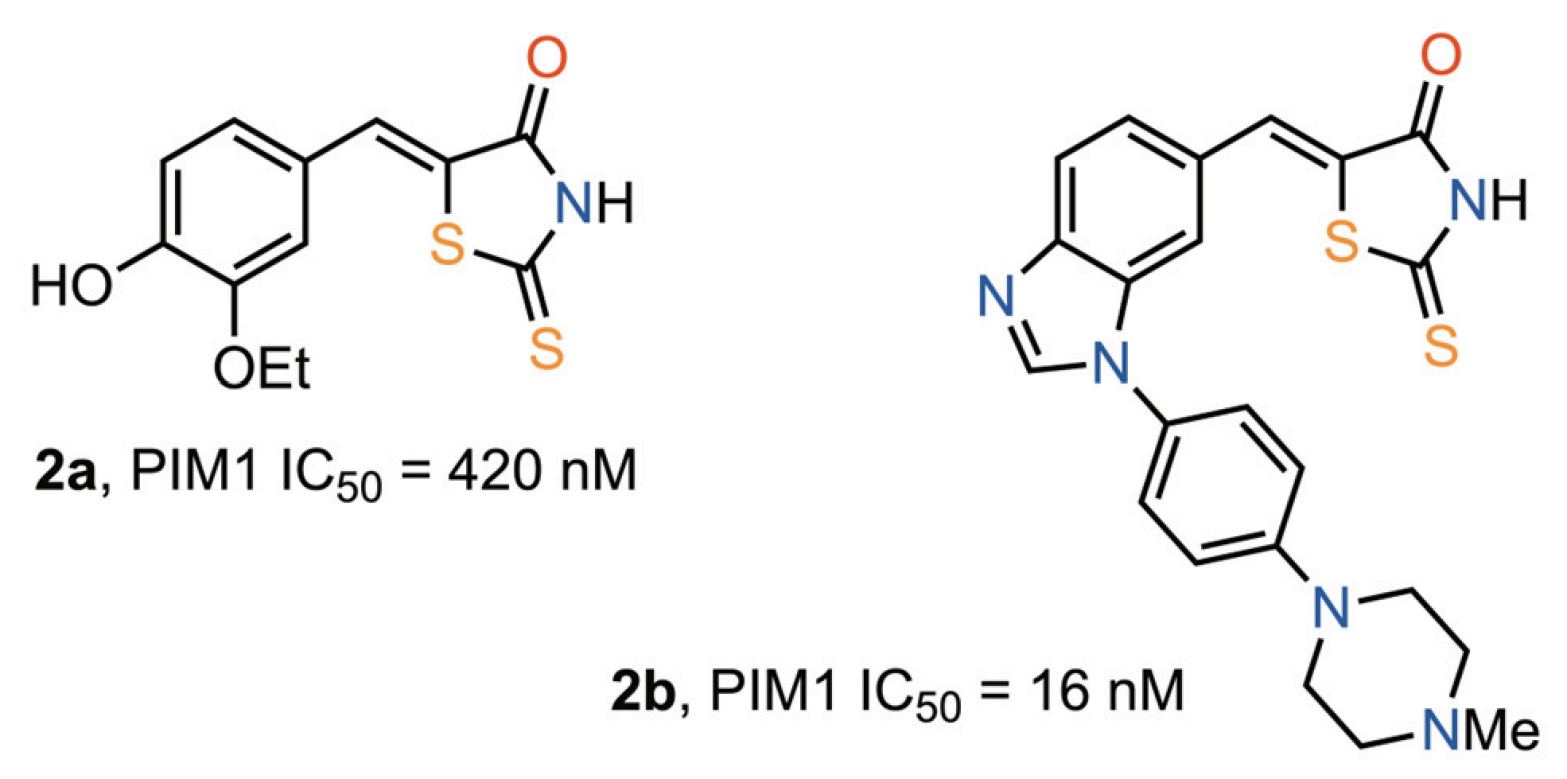
5-Arylidene-2-thioxo-4-thiazolidinone PIM kinase inhibitors [15].

**Figure 4:**
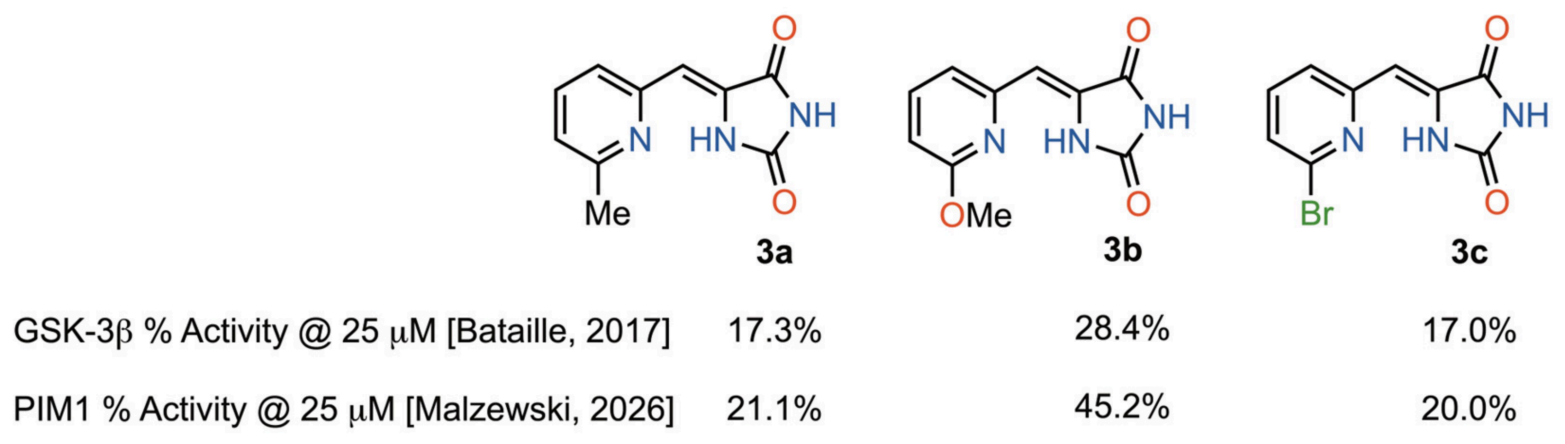
Evaluation of 5-(heteroarylmethylene)hydantoins as GSK-3β and PIM kinase inhibitors [14,17].

**Scheme 1:**
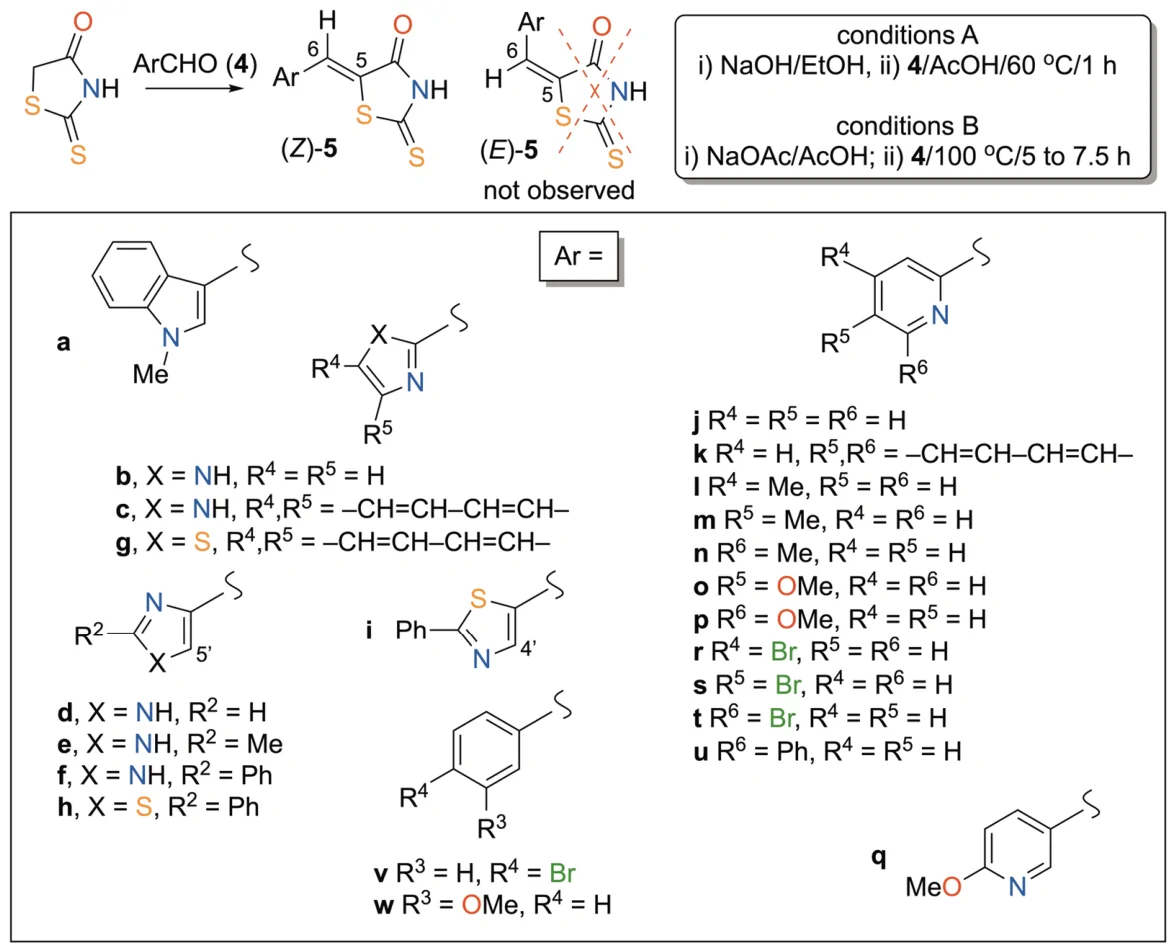
Synthesis of 5-heteroarylidene-2-thioxo-4-thiazolidinones.

**Figure 5:**
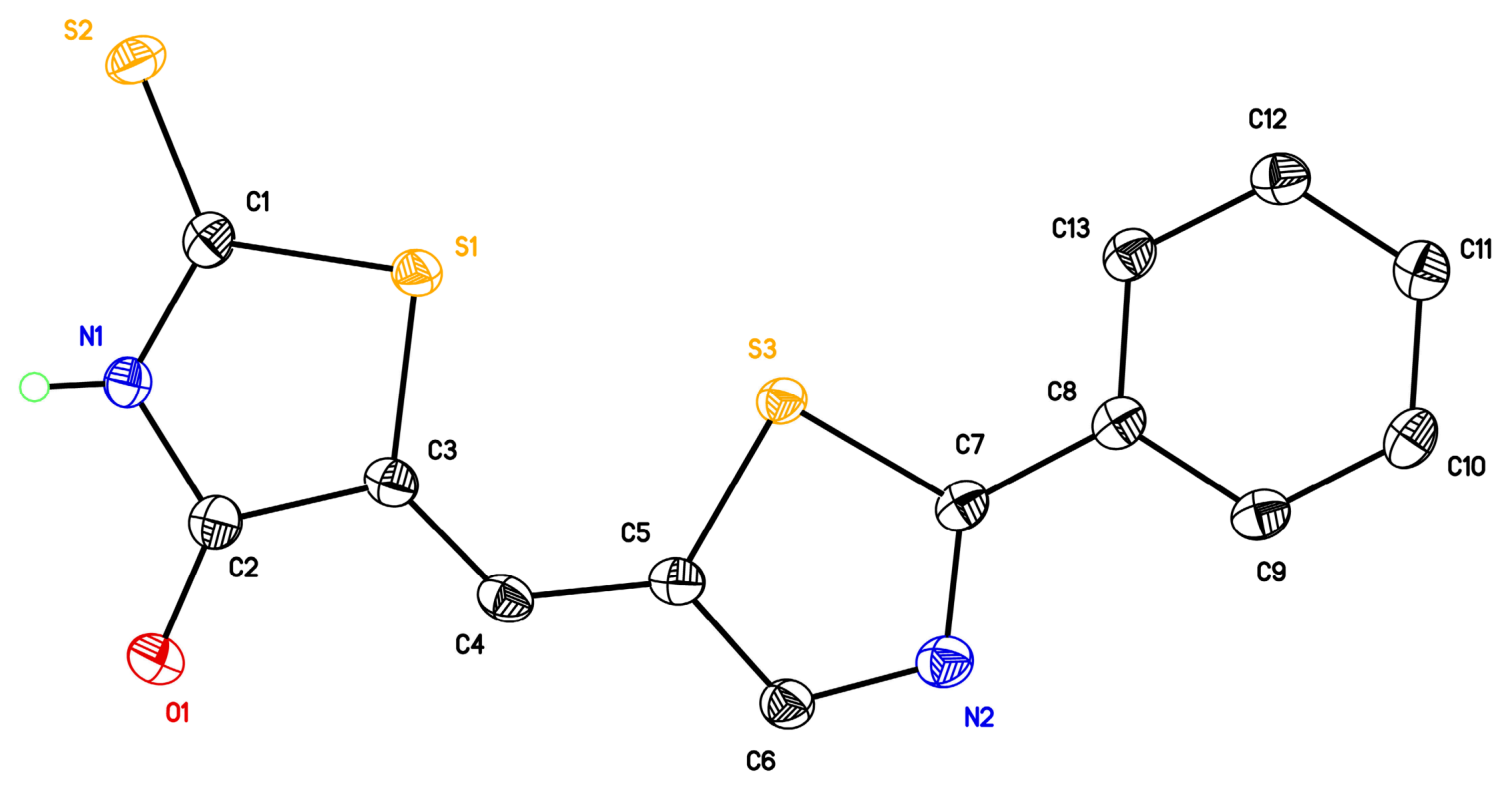
Ellipsoid plot of (*Z*)-5-[(2′-Phenylthiazo-5-yl)methylene]-2-thioxo-4-thiazolidinone (**5i**) (50%) with hydrogen atoms (except NH) and lattice solvent (d_6_-DMSO) omitted for clarity (CCDC 2574955). Atomic numbering does not correspond to nomenclature numbering.

**Figure 6:**
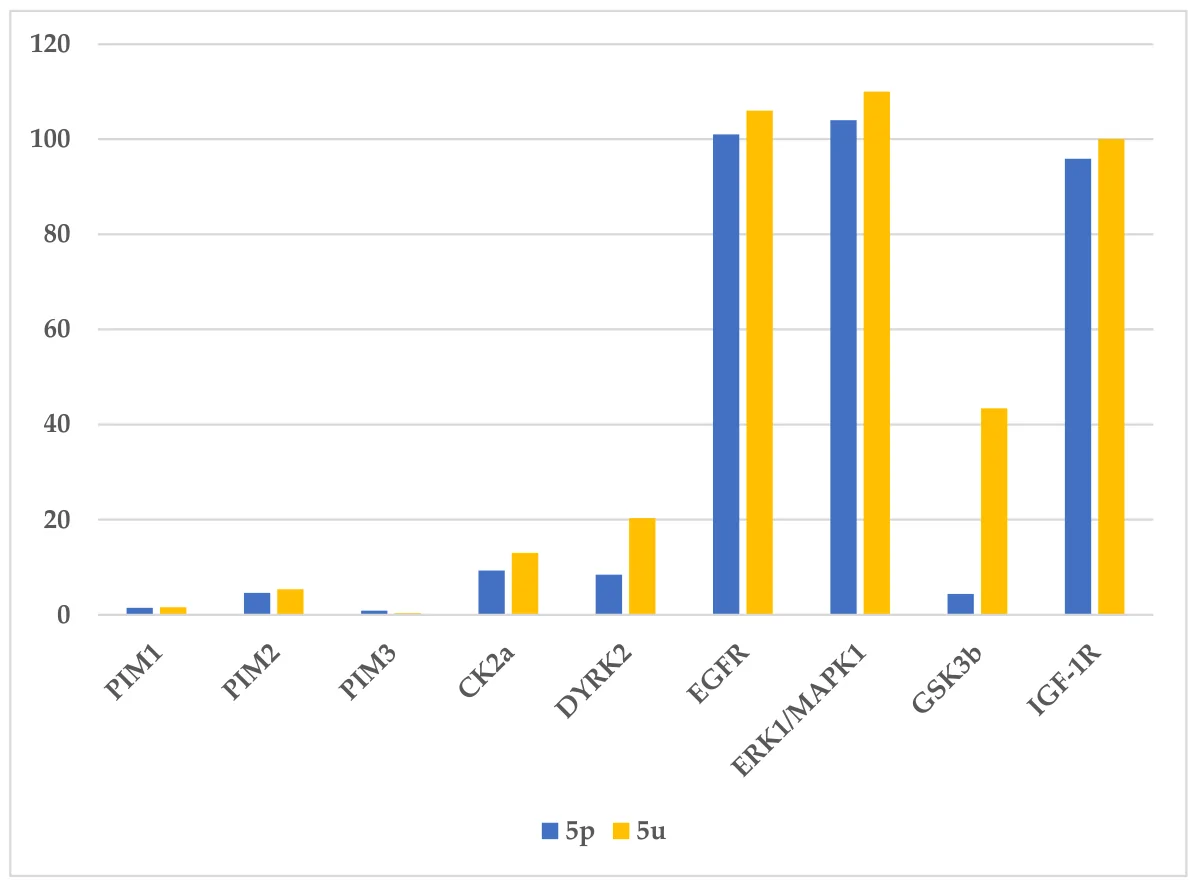
Percent (%) residual kinase activity @ 5 μM.

**Figure 7:**
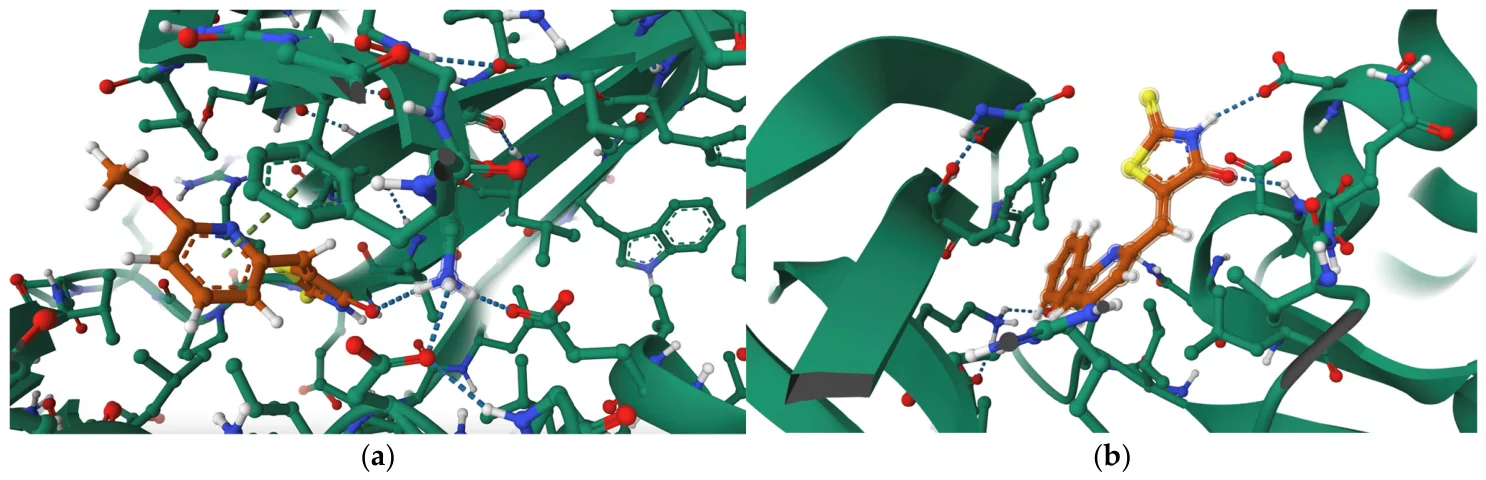
(**a**) Docking picture for **5p** into PDB 3F2A, showing interaction of the 2-thioxo-4-thiazolidinone carbonyl with the salt bridge formed between Asp186 (foreground) and Lys67 (enter of figure). This docking picture also displays the pi-stacking between Phe49 and the heteroarylidene group.; (**b**) Docking picture for **5u** into PDB3F2A, showing hydrogen bonding between the carbonyl group and the N–H of the 2-thioxo-4-thiazoldinone are hydrogen bonded with the backbone N–H of Asp128 and the carboxylate of Asp131.

**Table 1:** Inhibitory activity of 5-heteroarylidene-2-thioxo-4-thiazolidinones.

Compd	Ar	Percent Yield (Reaction Conditions)	Remaining Percent PIM1 Activity @ 25 μM	PIM1 IC_50_	Docking Score (pdb 3F2A)
5-(Indolylmethylene)-, 5-(Imidazolylmethylene)-, and 5-(thiazolylmethylene)-2-thioxo-4-thiazolidinones					
**5a**	*N*-methylindol-3-yl	48% (A)	2.61%	ND	–7.8 ^a^
**5b**	2-imidazolyl	67% (B)	30.7%	ND	–6.0 ^b^
**5c**	1,3-benzoimidazo-2-yl	68% (B)	ND	479 ± 9 nM	–7.0 ^b^
**5d**	1*H*-imidazol-4-yl	91% (B)	24.7%	62,800 ± 1200 nM	–5.8 ^b^
**5e**	2-methyl-1*H*-imidazol-4-yl	51% (B)	21.8%	ND	–6.2 ^b^
**5f**	2-phenyl-1*H*-imidazol-4-yl	71% (B)	ND	238 ± 6 nM	–8.1 ^a^
**5g**	1,3-benzothiazo-2-yl	78% (B)	0.78%	144 ± 12 nM	–6.6 ^a^
**5h**	2-phenylthiazo-4-yl	89% (B)	1.37%	1.29 ± 0.02 nM ^e^	–8.2 ^c^
**5i**	2-phenylthiazo-5-yl	52% (B)	1.31%	24.2 ± 0.9 nM	–7.1 ^d^
5-[(pyridinyl)methylene]-2-thioxo-4-thiazolidinones					
**5j**	2-pyridinyl	76% (A)	3.61%	ND	–6.6 ^d^
**5k**	2-quinolinyl	65% (B)	0.005%	15.0 ± 1.2 nM ^f^	–8.8 ^d^
**5l**	4-methyl-2-pyridinyl	77% (A)	1.70%	ND	–6.9 ^b^
**5m**	5-methyl-2-pyridinyl	82% (A)	0.55%	128 ± 16 nM	–7.0 ^b^
**5n**	6-methyl-2-pyridinyl	74% (A)	0.45%	224 ± 11 nM	–7.1 ^b^
**5o**	5-methoxy-2-pyridinyl	78% (B)	ND	144 ± 9 nM	–6.4 ^b^
**5p**	6-methoxy-2-pyridinyl	75% (A)	0.15%	2.60 ± 0.29 nM	–6.8 ^b^
**5q**	6-methoxy-3-pyridinyl	43% (B)	ND	115 ± 4 nM	–6.4 ^b^
**5r**	4-bromo-2-pyridinyl	72% (A)	1.67%	108 ± 6 nM	–6.7 ^b^
**5s**	5-bromo-2-pyridinyl	80% (A)	0.18%	16.1 ± 1.3 nM	–6.6 ^b^
**5t**	6-bromo-2-pyridinyl	78% (A)	0.25%	10.8 ± 1.6 nM	–7.7 ^b^
**5u**	6-phenyl-2-pyridinyl	87% (B)	ND	9.01 ± 0.67 nM	–9.3 ^c^
Substituted 5-[(phenyl)methylene]-2-thioxo-4-thiazolidinones					
**5v**	4-bromophenyl	81% (A)	0.90%	20.3 ± 0.9 nM	–7.5 ^b^
**5w**	3-methoxyphenyl	73% (A)	1.16%	33.7 ± 1.5 nM	–7.6 ^b^

^a^ The docking pose for this ligand is oriented such that the N–H of the 2-thioxo-4-thiazoldinone is hydrogen bonded with the carboxylate of Asp131 (Asp100 in Mcule). ^b^ The docking pose(s) for this ligand are oriented such that the carbonyl group of the 2-thioxo-4-thiazoldinone is in proximity to the salt bridge between Lys67 and Asp186 (Lys36 and Asp155 in Mcule) along with face-to-face pi-stacking between the heteroarylidene group and Phe49 (Phe18 in Mcule). ^c^ The docking pose(s) for this ligand are oriented such that the carbonyl group and the N–H of the 2-thioxo-4-thiazoldinone are hydrogen bonded with the backbone N–H of Asp128 and the carboxylate of Asp131 (Asp97 and Asp100 respectively in Mcule). ^d^ The docking pose for this ligand does not indicate any hydrogen bonding interactions. ^e^ Bataille et al. reported a PIM1 kinase IC_50_ = 6.7 ± 3.1 nM [14]. ^f^ Bataille et al. reported a PIM1 kinase IC_50_ = 55 nM [14].

**Table 2:** Kinase selectivity evaluation @ 5 μM. Values are presented as percent of residual activity.

Compd	PIM1	PIM2	PIM3	CK2a	DYRK2	EGFR	ERK2/ MAPK1	GSK3β	IGF-1R
**5p**	1.44%	4.59%	0.835%	9.28%	8.44%	101%	104%	4.38%	95.9%
**5u**	1.58%	5.36%	0.335%	13.0%	20.3%	106%	110%	43.4%	100%

## Data Availability

The data presented in this study are available on request from the corresponding author due to legal reasons.

## Supplementary Materials

The following supporting information can be downloaded at: https://www.mdpi.com/article/10.3390/molecules31193413/s1, X-ray crystallography data for **5i**, ^1^H and ^13^C NMR spectra of synthesized compounds and HRMS spectra of new compounds.
