# Supplementary material for: Synthesis and Evaluation of 5-Heteroarylidene-2-thioxo-4-thiazolidinones as PIM1 Kinase Inhibitors
Source: Molecules. 2026 Sep 25;31(19):3413. doi: 10.3390/molecules31193413 (PMC13666029; doi:10.3390/molecules31193413)

Supporting Information for

Synthesis and evaluation of 5-heteroarylidene-2-thioxo-4-thiazoldinones as PIM1 kinase inhibitors

Kelly Malzewski, Alexander N. Erickson and William A. Donaldson

Department of Chemistry, Marquette University, P. O. Box 1881, Milwaukee, WI 53201-1881, USA

This document includes:

X-ray Crystallography of **5i** S4-S6

^{1}H NMR spectrum of **5a** (d6-DMSO) S7

1H NMR spectrum of **5a** (d6-acetone) S8

^{13}C NMR spectrum of **5a** (d6-DMSO) S9

^{1}H NMR spectrum of **5b** (d6-DMSO) S10

^{13}C NMR spectrum of **5b** (d6-DMSO) S11

^{1}H NMR spectrum of **5c** (d6-DMSO) S12

^{13}C NMR spectrum of **5c** (d6-DMSO) S13

^{1}H NMR spectrum of **5d** (d6-DMSO) S14

^{13}C NMR spectrum of **5d** (d6-DMSO) S15

^{1}H NMR spectrum of **5e** (d6-DMSO) S16

^{13}C NMR spectrum of **5e** (d6-DMSO) S17

^{1}H NMR spectrum of **5f** (δ13.0 to 0.0 ppm) S18

^{1}H NMR spectrum of **5f** (δ10.0 to 0.0 ppm) S19

^{13}C NMR spectrum of **5f** (d6-DMSO) S20

^{1}H NMR spectrum of **5g** (d6-DMSO) S21

^{13}C NMR spectrum of **5g** (d6-DMSO) S22

^{1}H NMR spectrum of **5h** (d6-DMSO) S23

^{13}C NMR spectrum of **5h** (d6-DMSO) S24

^{1}H NMR spectrum of **5i** (d6-DMSO) S25

^{13}C NMR spectrum of **5i** (d6-DMSO) S26

^{1}H NMR spectrum of **5j** (d6-DMSO) S27

^{13}C NMR spectrum of **5j** (d6-DMSO) S28

^{1}H NMR spectrum of **5k** (d6-DMSO) S29

^{13}C NMR spectrum of **5k** (d6-DMSO) S30

^{1}H NMR spectrum of **5l** (d6-DMSO) S31

^{13}C NMR spectrum of **5l (d6-DMSO)** S32
^{1}H NMR spectrum of **5m** (d6-DMSO) S33
^{13}C NMR spectrum of **5m** (d6-DMSO) S34
^{1}H NMR spectrum of **5n** (d6-DMSO) S35
^{13}C NMR spectrum of **5n** (d6-DMSO) S36
^{1}H NMR spectrum of **5o** (d6-DMSO) S37
^{13}C NMR spectrum of **5o** (d6-DMSO) S38
^{1}H NMR spectrum of **5p** (d6-DMSO) S39
^{13}C NMR spectrum of **5p** (d6-DMSO) S40
^{1}H NMR spectrum of **5q** (d6-DMSO) S41
^{13}C NMR spectrum of **5q** (d6-DMSO) S42
^{1}H NMR spectrum of **5r** (d6-DMSO) S43
^{13}C NMR spectrum of **5r** (d6-DMSO) S44
^{1}H NMR spectrum of **5s** (d6-DMSO) S45
^{13}C NMR spectrum of **5s** (d6-DMSO) S46
^{1}H NMR spectrum of **5t** (d6-DMSO) S47
^{13}C NMR spectrum of **5t** (d6-DMSO) S48
^{1}H NMR spectrum of **5u** (d6-DMSO) S49
^{13}C NMR spectrum of **5u** (d6-DMSO) S50
^{1}H NMR spectrum of **5v** (d6-DMSO) S51
^{1}H NMR spectrum of **5w** (d6-DMSO) S52
^{13}C NMR spectrum of **5w** (d6-DMSO) S53
HRMS spectrum of **5c** S54
HRMS spectrum of **5d** S55
HRMS spectrum of **5e** S56
HRMS spectrum of **5f** S57
HRMS spectrum of **5g** S58
HRMS spectrum of **5i** S59
HRMS spectrum of **5l** S60
HRMS spectrum of **5m** S61
HRMS spectrum of **5n** S62

HRMS spectrum of **5o** S62

HRMS spectrum of **5p** S63

HRMS spectrum of **5q** S64

HRMS spectrum of **5r** S65

HRMS spectrum of **5s** S66

HRMS spectrum of **5t** S67

HRMS spectrum of **5u** S68

X-Ray Crystallography

General Considerations

Compound **5i** was characterized at the Marquette University Diffraction Facility. A small specimen was retrieved, placed on a microscope slide, coated in inert oil, mounted on a MiTeGen Dual Thickness MicroMount and placed on a goniometer head under a cold stream of N_2 gas. Data were collected at 100(2) K on a Bruker D8 Venture 4-circle diffractometer coupled to a Photon III C14 CMOS area detector with Cu K_α radiation (λ=1.54184 Å) provided by a Incoatec IµS 3.0 micro-focus Cu X-ray source running at 50.0 kV and 1.1 mA. All data manipulations for **5i** were carried out using the Bruker APEX-6 Software Suite. [Ref S1] Data were reduced, correcting for absorption, using the program SADABS. [Ref S2] Space group assignment was done using XPREP within the SHELXTL suite of programs. [Ref S3] Using the ShelXle graphical interface, the structure was solved by dual space methods employed by ShelXT and refined by full-matrix least-squares methods against F^2 using ShelXL with scattering factors and anomalous dispersion terms taken from the literature. [Ref S4]. All non-hydrogen atoms not apparent from the initial solution were found from difference Fourier maps, and all heavy atoms were refined anisotropically.

CCDC entry 2574955 contains the supplementary crystallographic data for this paper. These data can be obtained free of charge via http://www.ccdc.cam.ac.uk/data_request/cif, or by emailing data_request@ccdc.cam.ac.uk, or by contacting The Cambridge Crystallographic Data Centre, 12 Union Road, Cambridge CB2 1EZ, UK; fax: +44 1223 336033.

The relevant crystallographic and structure refinement data for **5i** are given in Table S1.

[Ref S1]: Bruker (2025). Apex-6 (Version 2024.9-1) and SAINT (Version 8.41), Bruker AXS Inc., Madison, Wisconsin, USA.
[Ref S2]: (*a*) SADABS (Version 2016/2) Bruker AXS Inc., Madison, Wisconsin, USA. (*b*) Krause, L.; Herbst-Irmer, R.; Sheldrick, G.M.; Stalke, D. Comparison of Silver and Molybdenum Microfocus X-ray Sources for Single-Crystal Structure Determination. *J. Appl. Cryst.* **2015**, *48*, 3-10.
[Ref S3]: XPREP (Version 2014/2), Bruker AXS Inc., Madison, Wisconsin, USA.
[Ref S4]: ShelXle: (*a*) Hübschle, C. B.; Sheldrick, G. M.; Dittrich, B. ShelXle: A Qt Graphical User Interface for SHELXL. *J. Appl. Cryst*. **2011**, *44*, 1281-1284. SHELXT: (*b*) Sheldrick, G. M. SHELXT - Integrated Space-Group and Crystal-Structure Determination. *Acta Crystallogr. Sect. A*: *Found. Adv.* **2015**, *A71*, 3-8. SHELXL: (*c*) Sheldrick, G. M. Crystal Structure Refinement with SHELX. *Acta Crystallogr. Sect. C: Struct. Chem.* **2015**, *C71*, 3-8. Scattering Factors: (*d*) *International Tables for Crystallography,* 3rd ed.; Kluwer Academic Publishers: Dordrecht, Netherlands, 2004; Vol C.

(Z)-5-[(2’-Phenylthiazo-5-yl)methylene]-2-thioxo-4-thiazolidinone (5i)

Crystallization Conditions for 5i.

Crystals were deposited from a solution of the material dissolved in d_6-DMSO in a 5mm NMR tube at ambient temperature.

Special Refinement Details for 5i.

All hydrogen atoms were found from difference Fourier maps and allowed to refine freely.

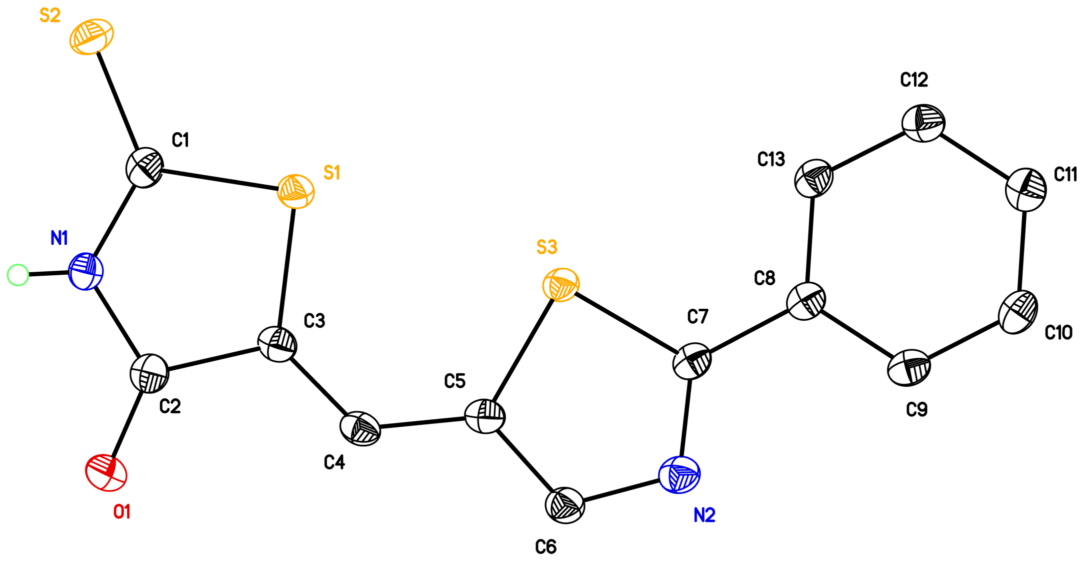

Figure S1. Ellipsoid plot of **5i** (50%) with hydrogen atoms (except N*H*) and lattice solvent (d_6-DMSO) omitted for clarity.

Table S1. Crystal and Refinement Data for **5i**.

Compound	**[(Z)-5-[(2'-Phenylthiazo-5-yl)methylene]-2-thioxo-4-thiazolidinone**, **5i**
Crystal Data	
Identification Code	DON3B
CCDC Accession Code	2574955
Empirical Formula	$C_{15}H_8D_6N_2O_2S_4$
Formula Weight	388.56
Color / Shape	Translucent Yellow / Needle
Crystal Size (mm)	0.462 × 0.022 × 0.011
Temperature (K)	100(2)
λ (Å)	1.54184 Cu K_α
Crystal System / Space Group	Monoclinic / *C*2/*c* (No. 15)
a (Å)	38.572(3)
b (Å)	5.1661(4)
c (Å)	17.2662(11)
α (°)	90
β (°)	102.583(3)
γ (°)	90
Volume ($Å^3$)	3357.9(4)
Z	8
Density (calculated) (g/cm^3)	1.537
Absorption Coefficient (μ) (mm^{-1})	5.287
F(000)	1584
Data Collection	
θ Range for Data Collection (°)	2.347 to 80.008
Index Ranges	$-48 \le h \le 45$, $-6 \le k \le 6$, $-21 \le l \le 45$
Total Reflections Collected	42176
Independent Reflections	3635 [R_{int} = 0.0979]
Observed Reflections [I>2σ(I)]	3111
Completeness to θ = 67.679° / θ_{max}	100.0 % / 98.8 %
Absorption Correction	multi-scan
Max. and Min. Transmission	0.7543 and 0.5932
Refinement	
Refinement Method	Full-matrix least-squares on F^2
Data / Restraints / Parameters	3635 / 0 / 264
Goodness-of-Fit on F^2	1.076
Final R Indices [I>2σ(I)]	R_1 = 0.0318, wR_2 = 0.0844
R Indices (all data)	R_1 = 0.0462, wR_2 = 0.0898
$\Delta\rho_{max}$ / $\Delta\rho_{min}$ ($e^- Å^{-3}$)	0.563 / -0.325

SpinWorks 4:

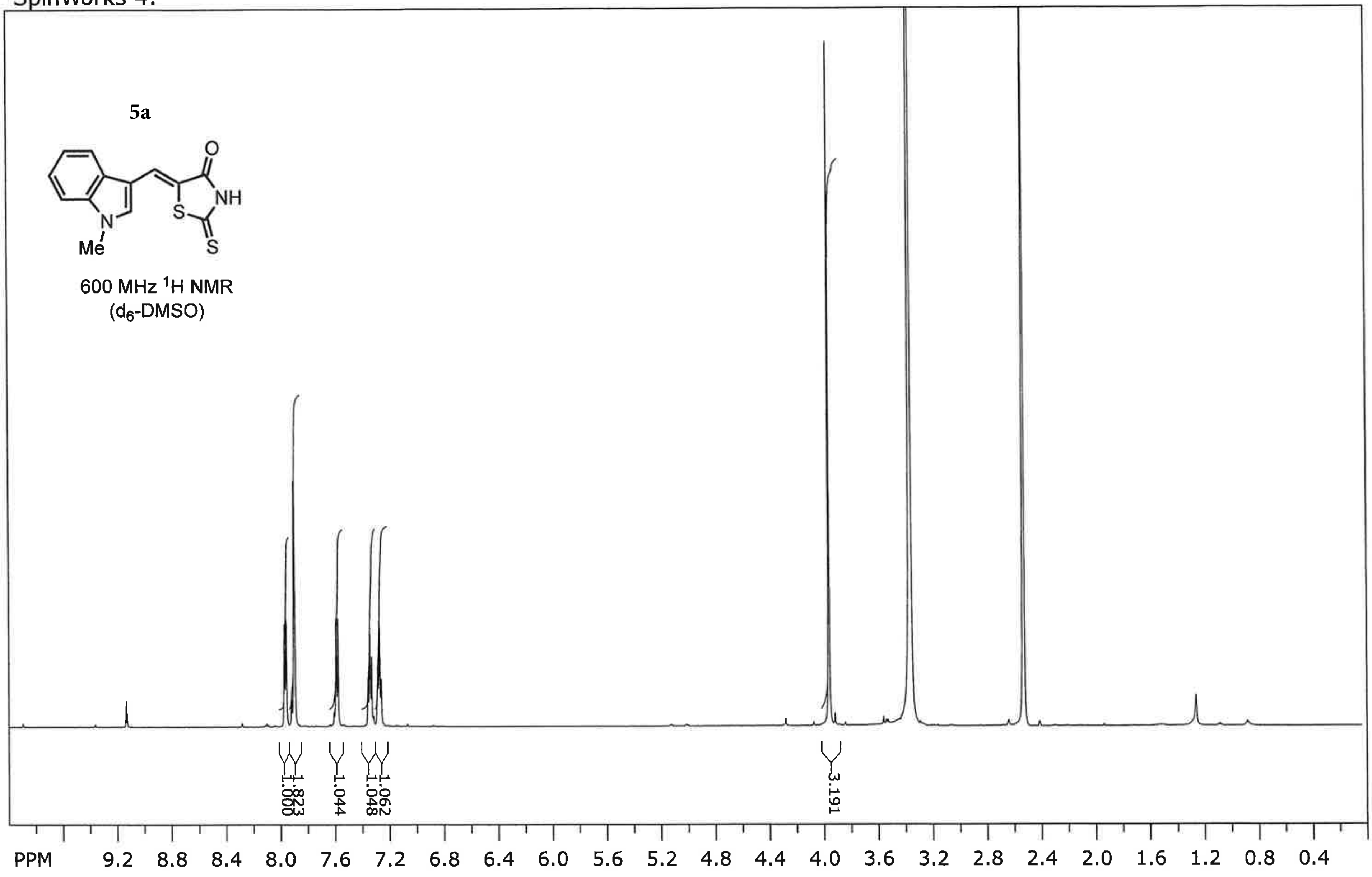

file: ...a\KM 060-069\KM-061-reetoh.fid\fid block# 1 expt: "s2pul"
transmitter freq.: 599.508636 MHz
time domain size: 32768 points
width: 9615.38 Hz = 16.0388 ppm = 0.293438 Hz/pt
number of scans: 32

freq. of 0 ppm: 599.505029 MHz
processed size: 32768 complex points
LB: 0.500 GF: 0.0000
Hz/cm: 239.744 ppm/cm: 0.39990

SpinWorks 4:

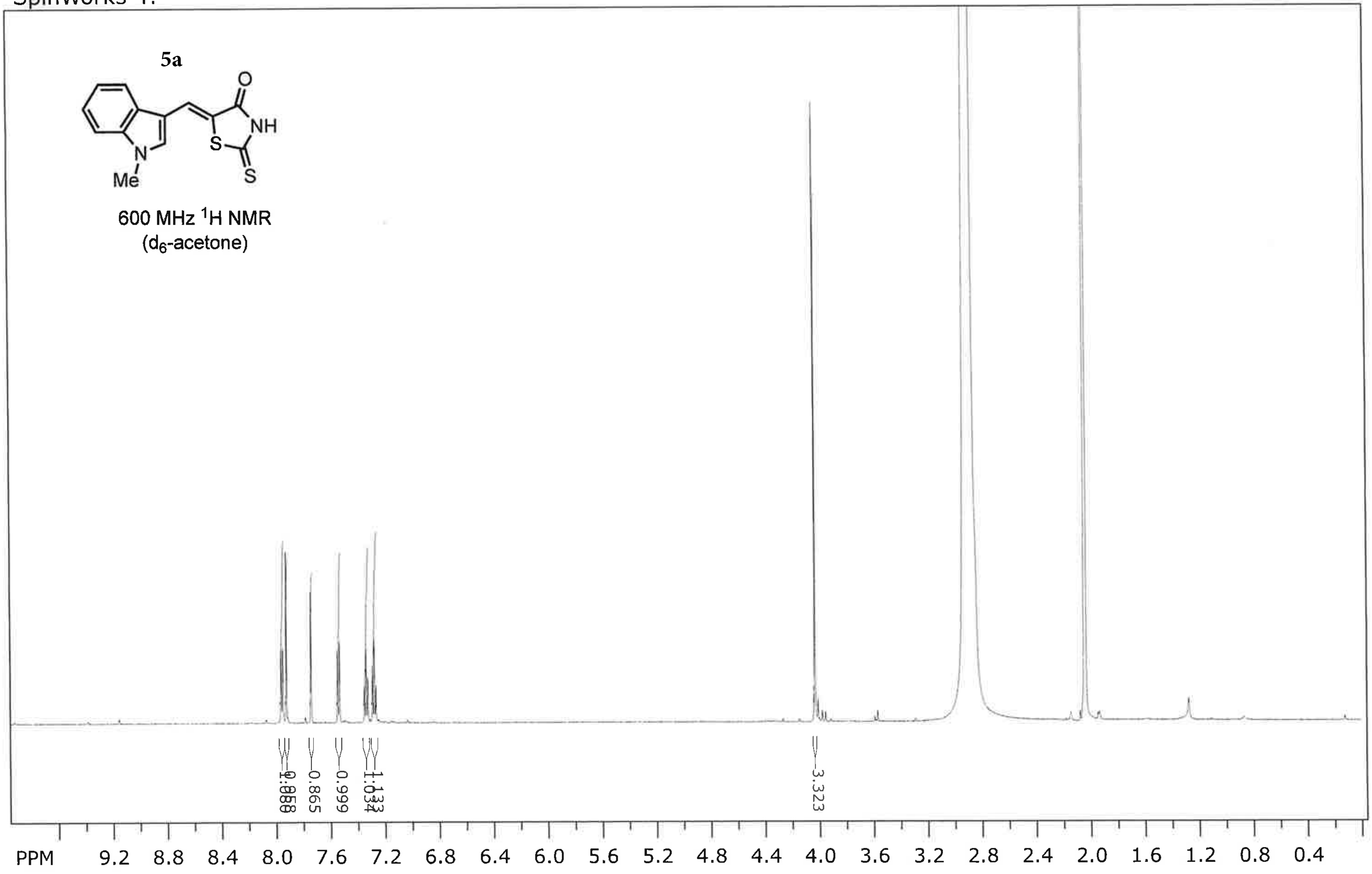

file: ...KM061_d6acetone_2026_06_29.fid\fid block# 1 expt: "s2pul"
transmitter freq.: 599.508853 MHz
time domain size: 32768 points
width: 9615.38 Hz = 16.0388 ppm = 0.293438 Hz/pt
number of scans: 16

freq. of 0 ppm: 599.505243 MHz
processed size: 32768 complex points
LB: 0.500 GF: 0.0000
Hz/cm: 239.744 ppm/cm: 0.39990

SpinWorks 4:

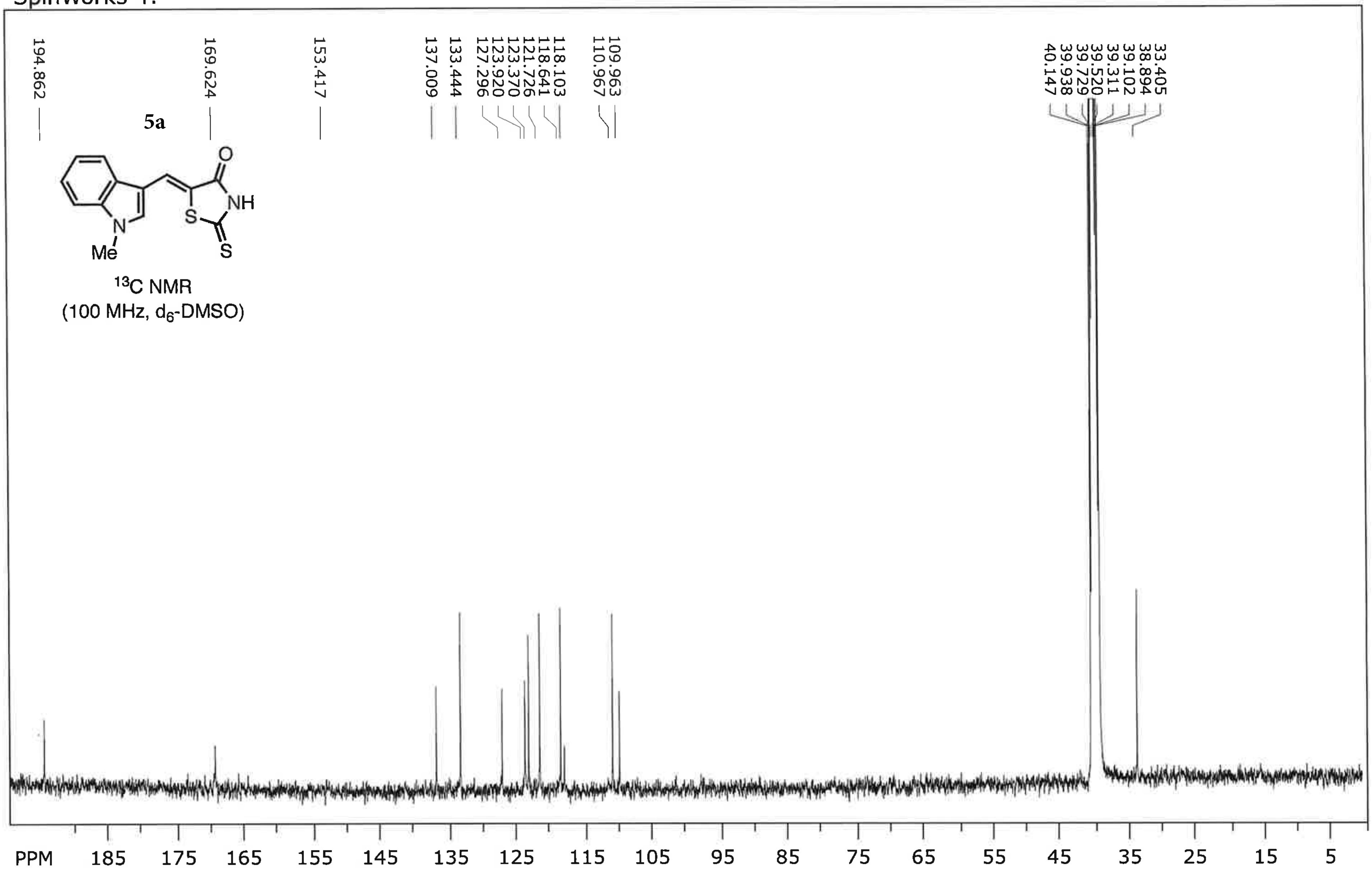

file: ...tra\KM 060-069\KM-061REC13.fid\fid block# 1 expt: "s2pul"
transmitter freq.: 100.522893 MHz
time domain size: 50000 points
width: 25000.00 Hz = 248.6996 ppm = 0.500000 Hz/pt
number of scans: 10000

freq. of 0 ppm: 100.511876 MHz
processed size: 65536 complex points
LB: 2.500 GF: 0.0000
Hz/cm: 803.889 ppm/cm: 7.99707

SpinWorks 4:

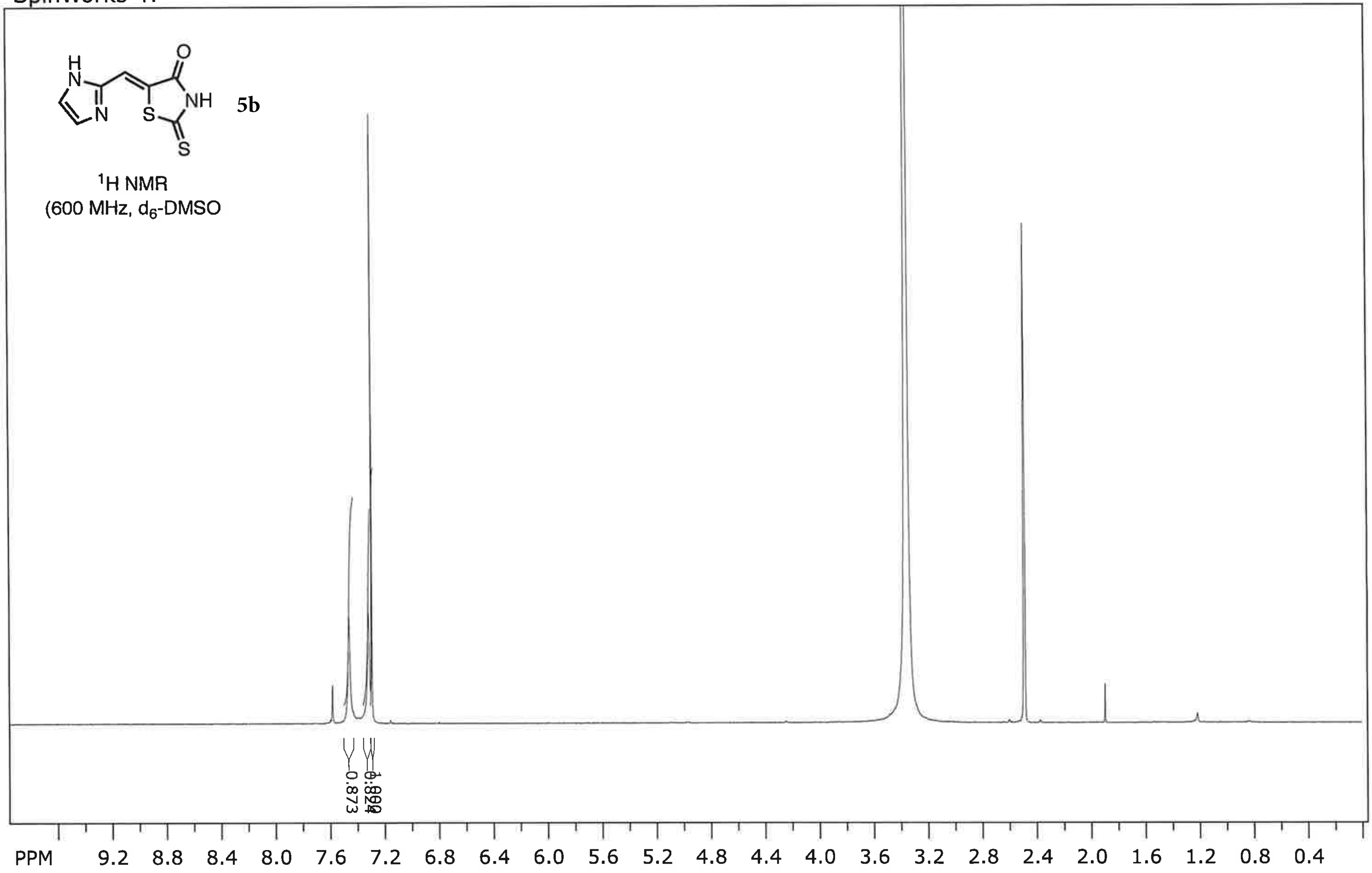

file: ...WD395_1H_d6DMSO_2025_12_03.fid\fid block# 1 expt: "s2pul"
transmitter freq.: 599.508636 MHz
time domain size: 32768 points
width: 9615.38 Hz = 16.0388 ppm = 0.293438 Hz/pt
number of scans: 8

freq. of 0 ppm: 599.505036 MHz
processed size: 32768 complex points
LB: 0.500 GF: 0.0000
Hz/cm: 239.744 ppm/cm: 0.39990

SpinWorks 4:

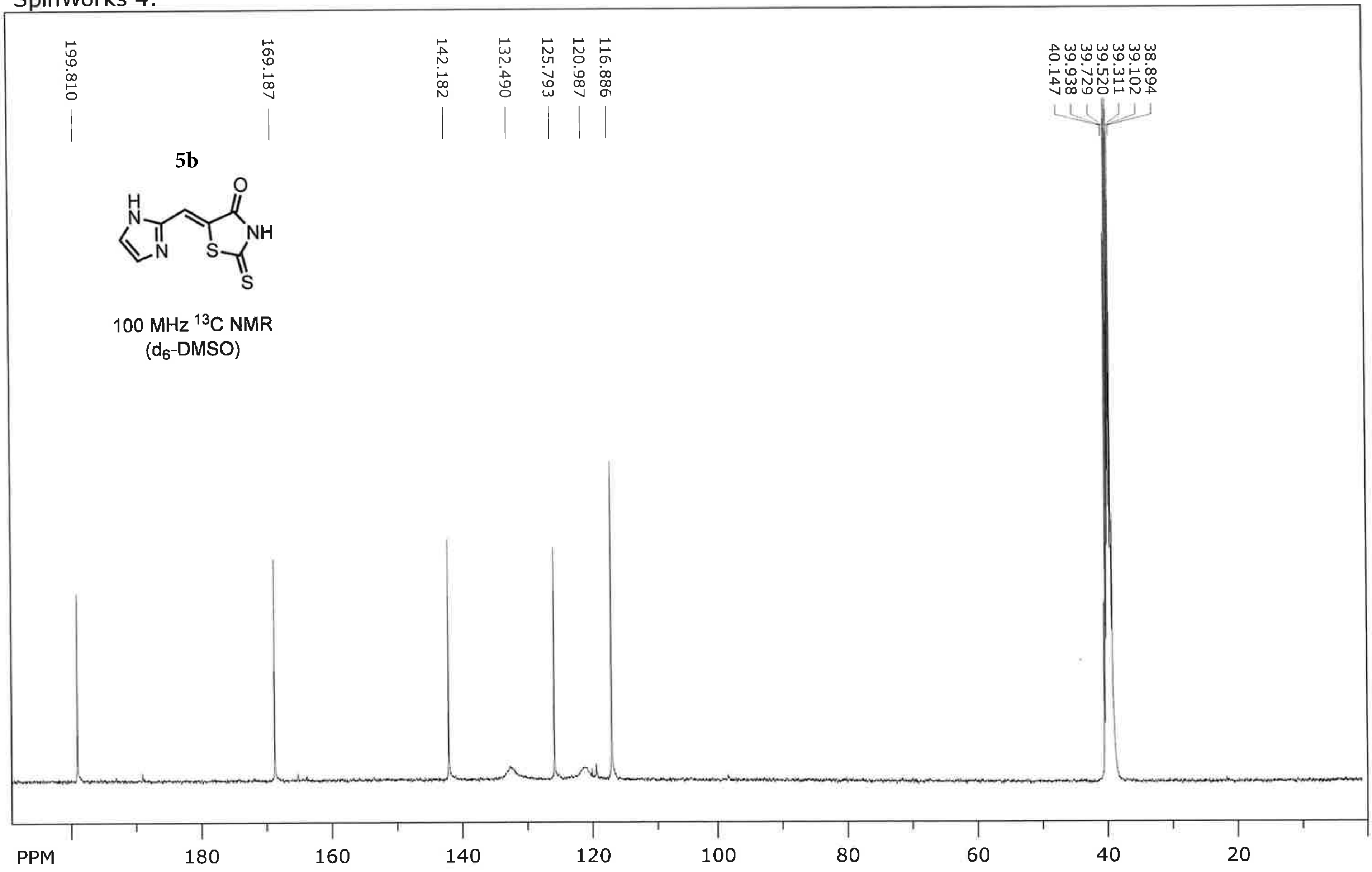

file: ...99\WD395_d6DMSO_2026_08_23.fid\fid block# 1 expt: "s2pul"
transmitter freq.: 100.522325 MHz
time domain size: 50000 points
width: 25000.00 Hz = 248.7010 ppm = 0.500000 Hz/pt
number of scans: 10000

freq. of 0 ppm: 100.511295 MHz
processed size: 65536 complex points
LB: 2.500 GF: 0.0000
Hz/cm: 844.444 ppm/cm: 8.40057

SpinWorks 4:

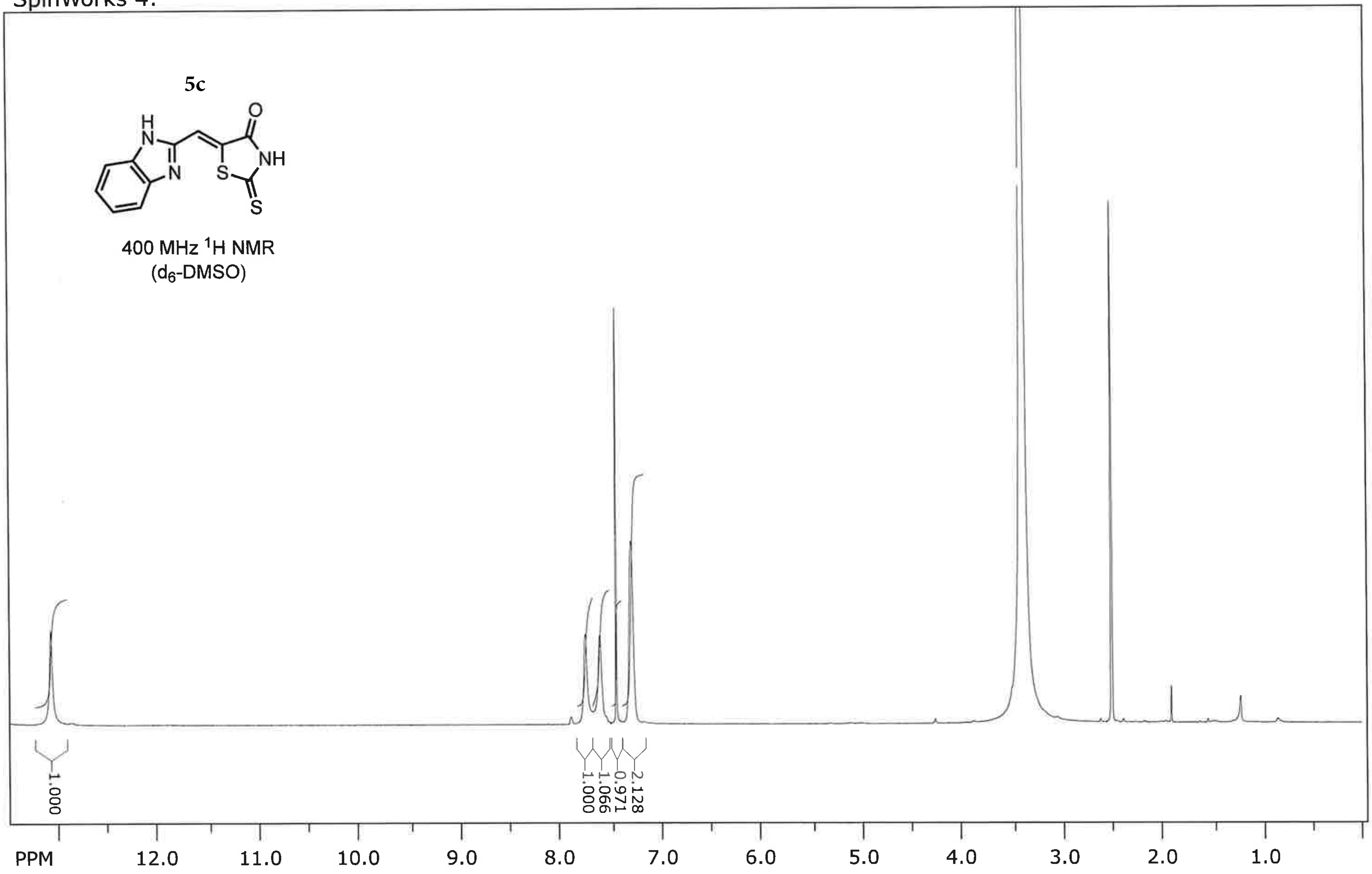

file: ...op\WD402_d6DMSO_2026_04_18.fid\fid block# 1 expt: "s2pul"
transmitter freq.: 599.508636 MHz
time domain size: 32768 points
width: 9615.38 Hz = 16.0388 ppm = 0.293438 Hz/pt
number of scans: 16

freq. of 0 ppm: 599.504931 MHz
processed size: 32768 complex points
LB: 0.500 GF: 0.0000
Hz/cm: 323.932 ppm/cm: 0.54033

SpinWorks 4:

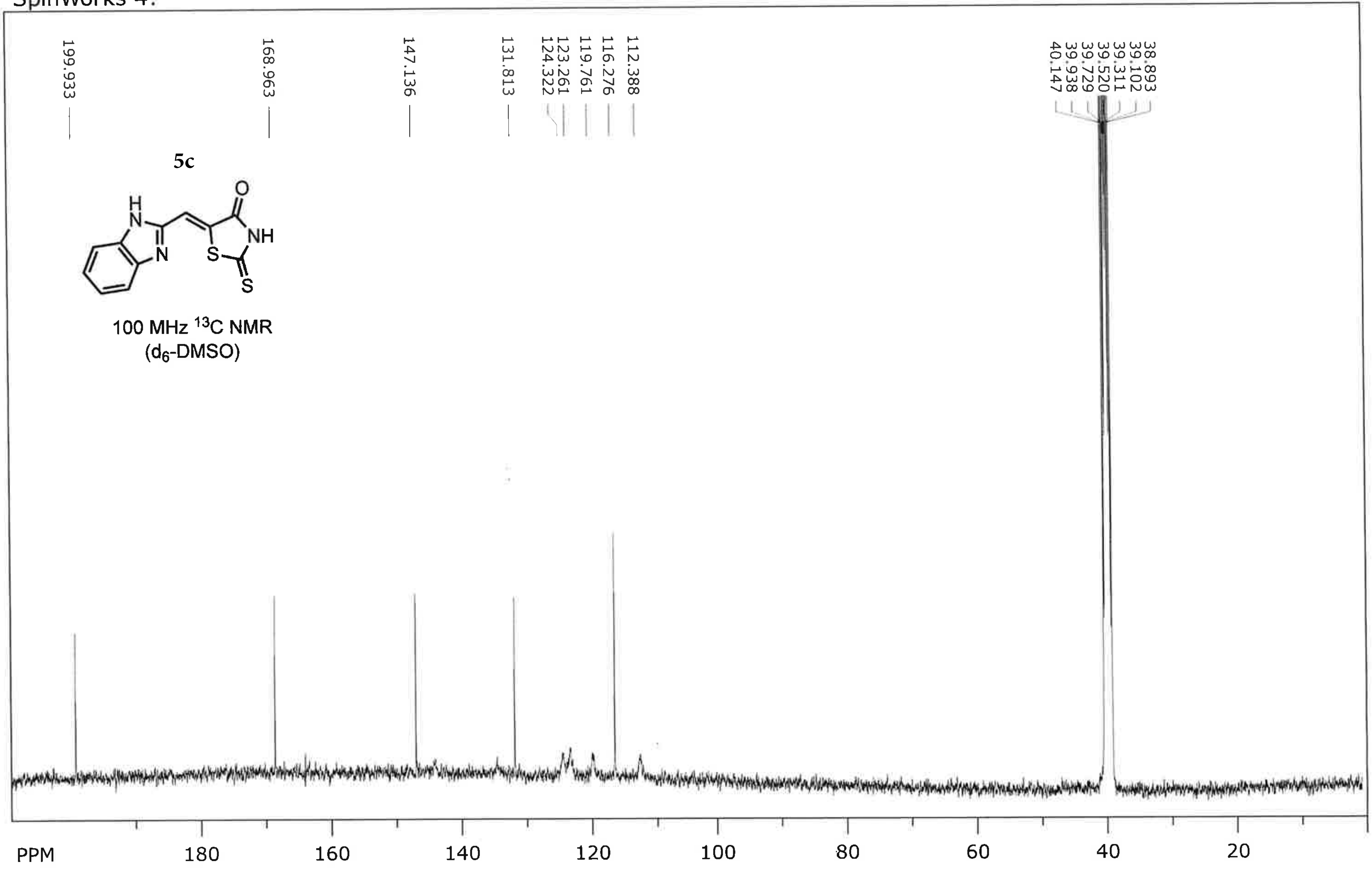

file: ...op\WD402_d6DMSO_2026_08_19.fid\fid block# 1 expt: "s2pul"
transmitter freq.: 100.522325 MHz
time domain size: 50000 points
width: 25000.00 Hz = 248.7010 ppm = 0.500000 Hz/pt
number of scans: 10000

freq. of 0 ppm: 100.511303 MHz
processed size: 65536 complex points
LB: 2.500 GF: 0.0000
Hz/cm: 843.889 ppm/cm: 8.39504

SpinWorks 4:

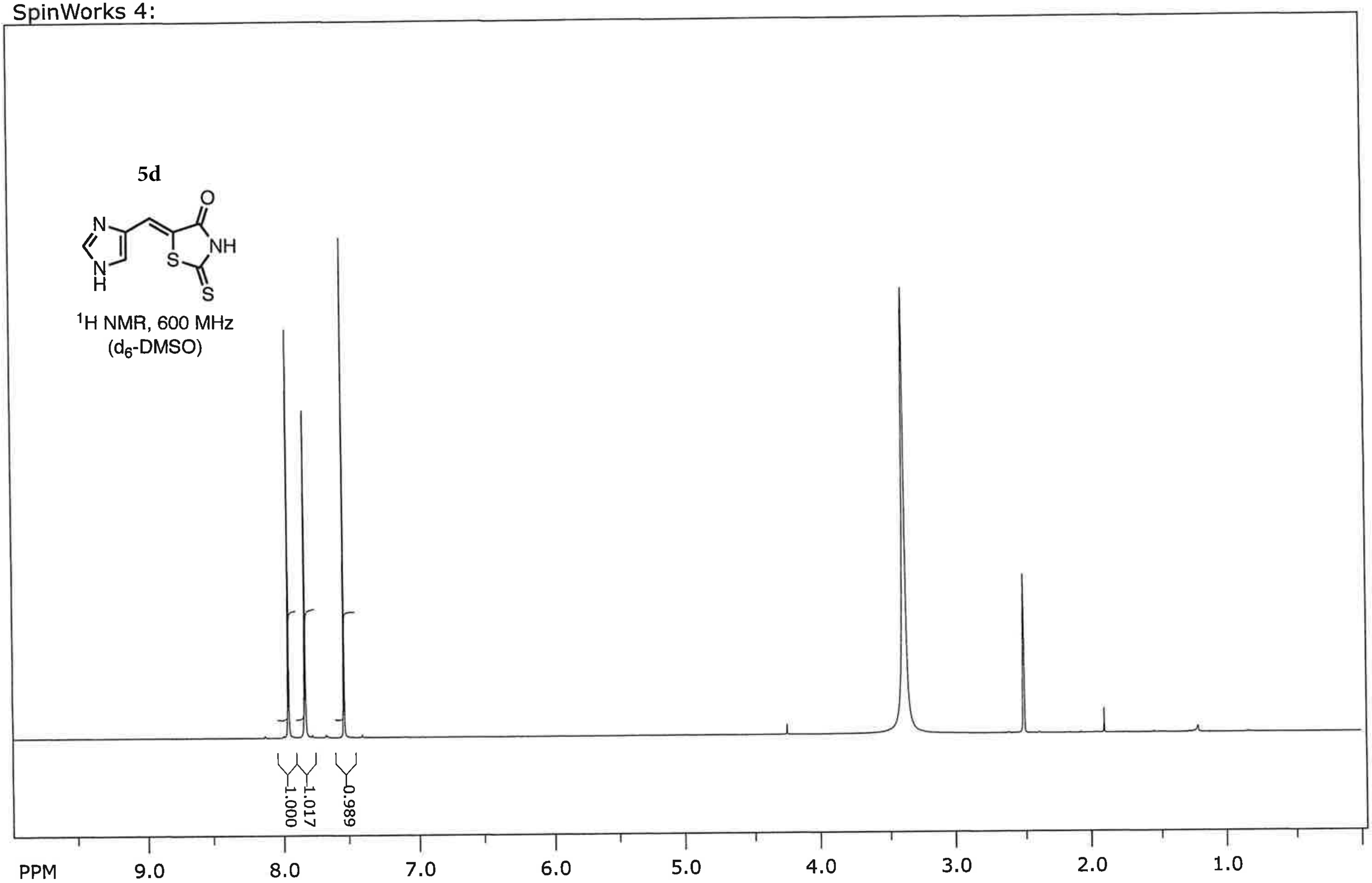

file: ...WD393_1H_d6DMSO_2025_11_07.fid\fid block# 1 expt: "s2pul"
transmitter freq.: 599.508636 MHz
time domain size: 32768 points
width: 9615.38 Hz = 16.0388 ppm = 0.293438 Hz/pt
number of scans: 8

freq. of 0 ppm: 599.505027 MHz
processed size: 32768 complex points
LB: 0.500 GF: 0.0000
Hz/cm: 239.957 ppm/cm: 0.40026

SpinWorks 4: SC

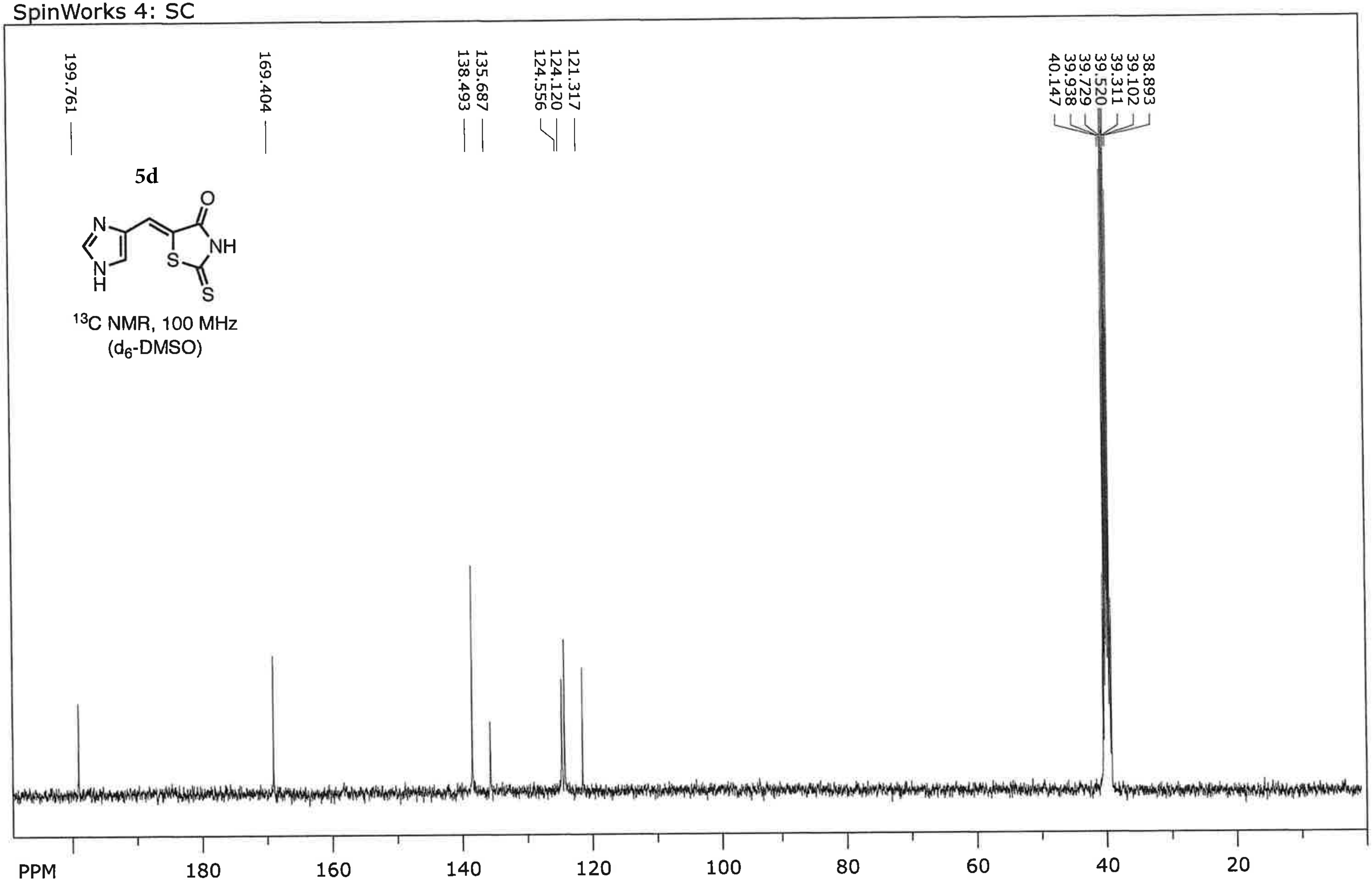

file: ...D393_13C_d6DMSO_2025_11_07.fid\fid block# 1 expt: "s2pul"
transmitter freq.: 100.522893 MHz
time domain size: 65536 points
width: 25000.00 Hz = 248.6996 ppm = 0.381470 Hz/pt
number of scans: 512

freq. of 0 ppm: 100.511875 MHz
processed size: 65536 complex points
LB: 2.500 GF: 0.0000
Hz/cm: 844.444 ppm/cm: 8.40052

SpinWorks 4:

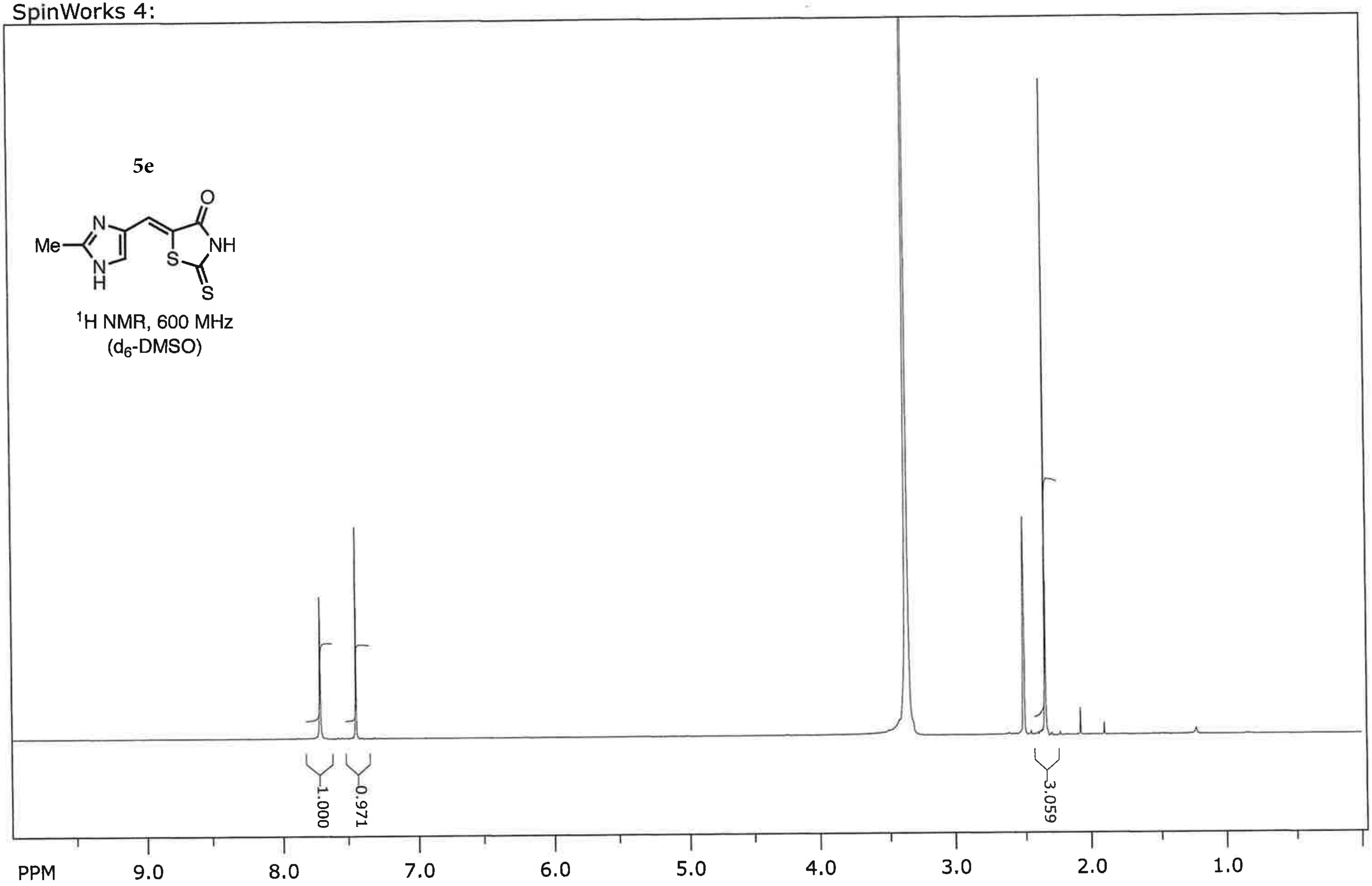

file: ...WD394_1H_d6DMSO_2025_11_07.fid\fid block# 1 expt: "s2pul"
transmitter freq.: 599.508636 MHz
time domain size: 32768 points
width: 9615.38 Hz = 16.0388 ppm = 0.293438 Hz/pt
number of scans: 8

freq. of 0 ppm: 599.505026 MHz
processed size: 32768 complex points
LB: 0.500 GF: 0.0000
Hz/cm: 239.957 ppm/cm: 0.40026

SpinWorks 4:

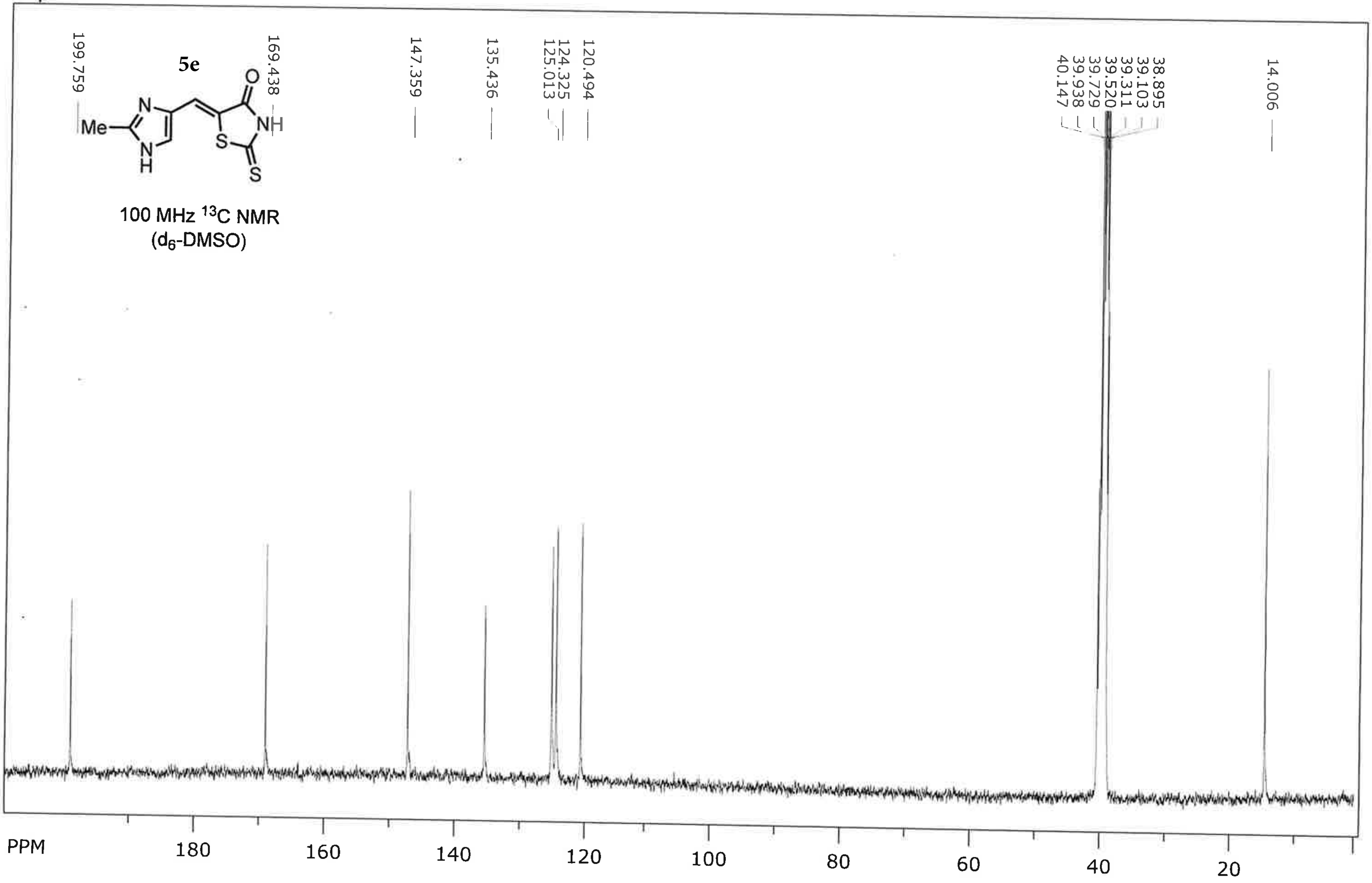

file: ...D394_13C_d6DMSO_2026_08_16.fid\fid block# 1 expt: "s2pul"
transmitter freq.: 100.522325 MHz
time domain size: 50000 points
width: 25000.00 Hz = 248.7010 ppm = 0.500000 Hz/pt
number of scans: 5000

freq. of 0 ppm: 100.511302 MHz
processed size: 65536 complex points
LB: 2.500 GF: 0.0000
Hz/cm: 844.444 ppm/cm: 8.40057

SpinWorks 4:

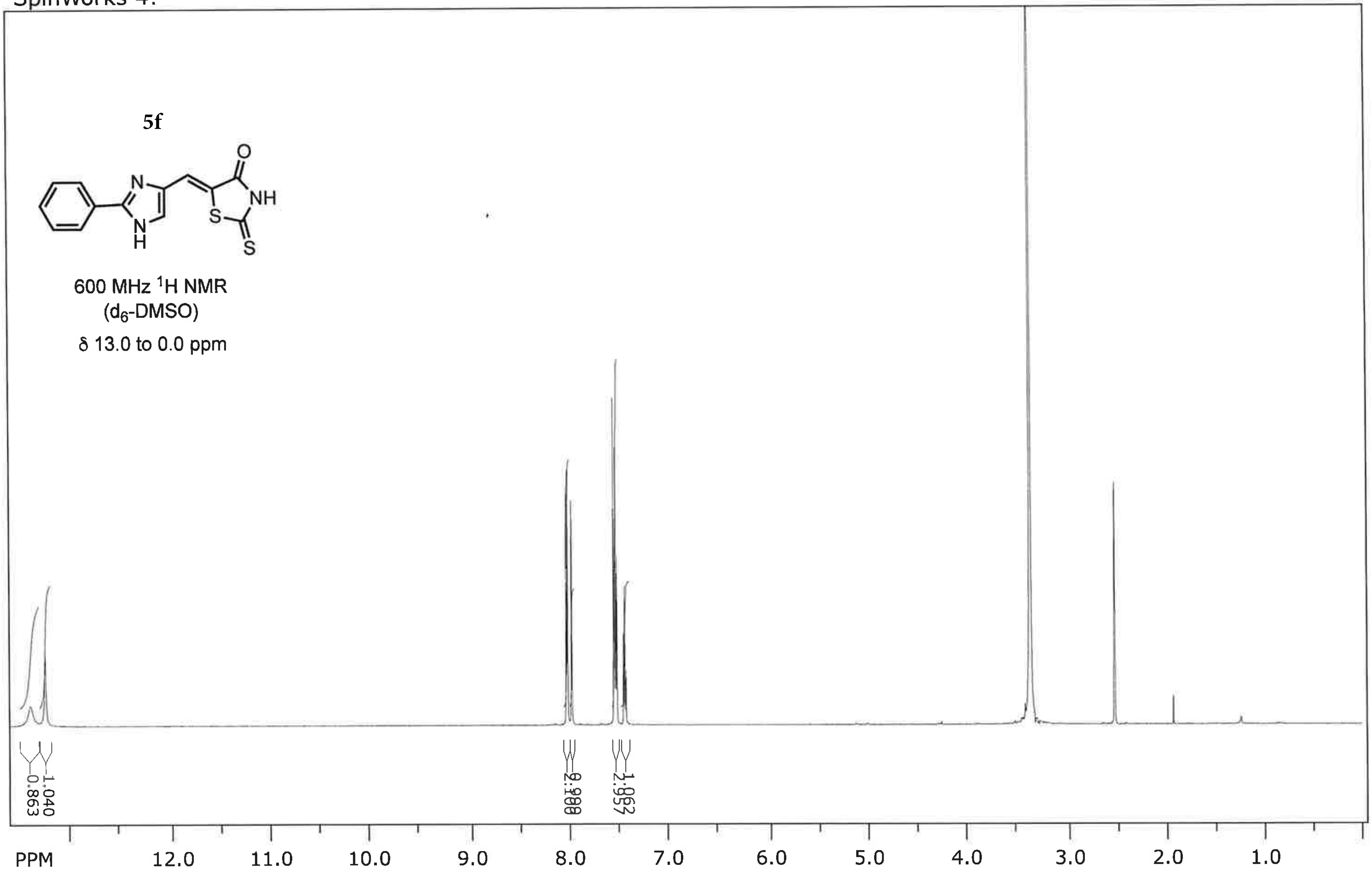

file: ...op\WD405_d6DMSO_2026_05_30.fid\fid block# 1 expt: "s2pul"
transmitter freq.: 599.508651 MHz
time domain size: 32768 points
width: 9615.38 Hz = 16.0388 ppm = 0.293438 Hz/pt
number of scans: 16

freq. of 0 ppm: 599.505046 MHz
processed size: 32768 complex points
LB: 0.500 GF: 0.0000
Hz/cm: 327.137 ppm/cm: 0.54567

SpinWorks 4:

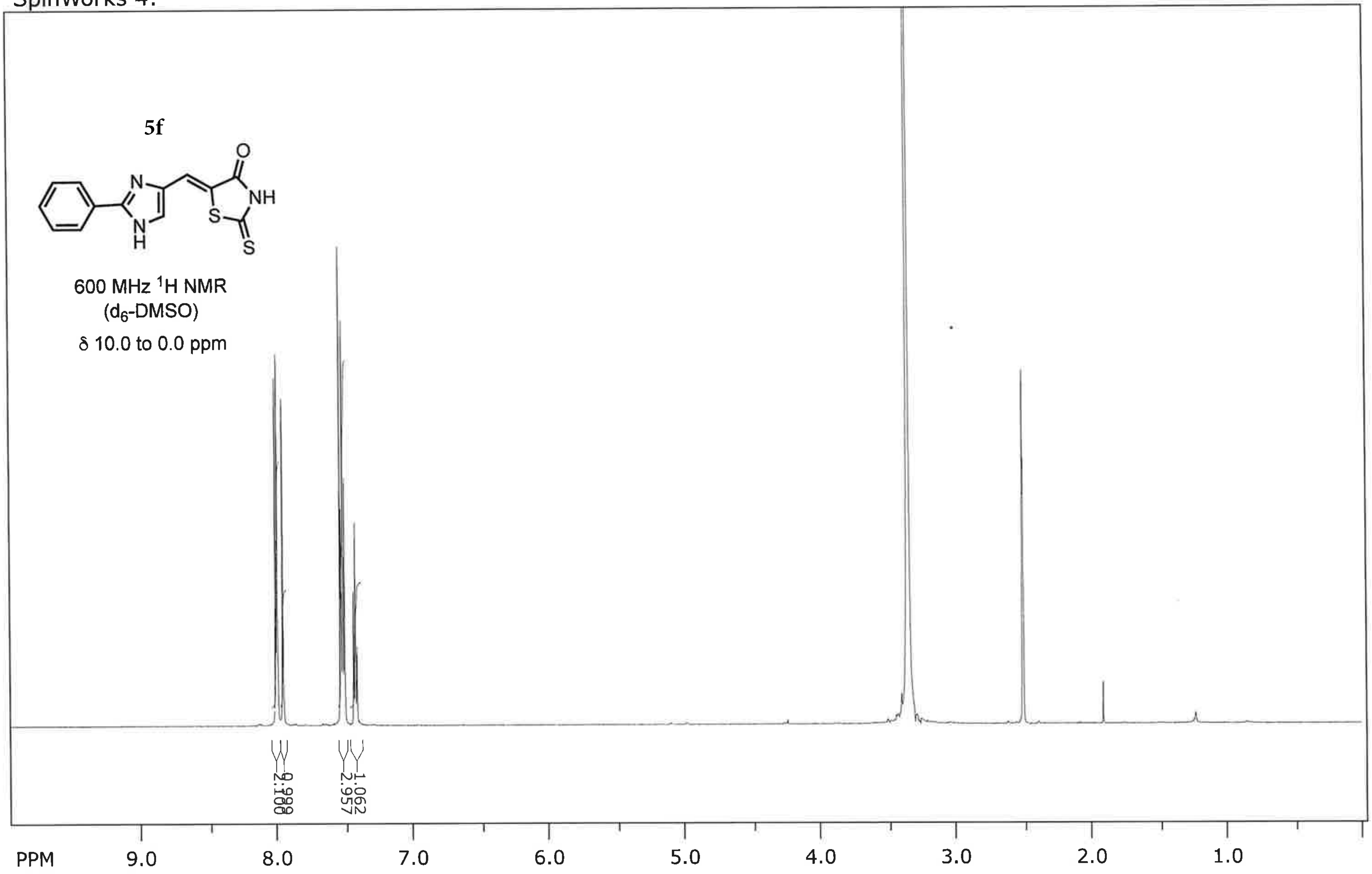

file: ...op\WD405_d6DMSO_2026_05_30.fid\fid block# 1 expt: "s2pul"
transmitter freq.: 599.508651 MHz
time domain size: 32768 points
width: 9615.38 Hz = 16.0388 ppm = 0.293438 Hz/pt
number of scans: 16

freq. of 0 ppm: 599.505046 MHz
processed size: 32768 complex points
LB: 0.500 GF: 0.0000
Hz/cm: 239.957 ppm/cm: 0.40026

SpinWorks 4:

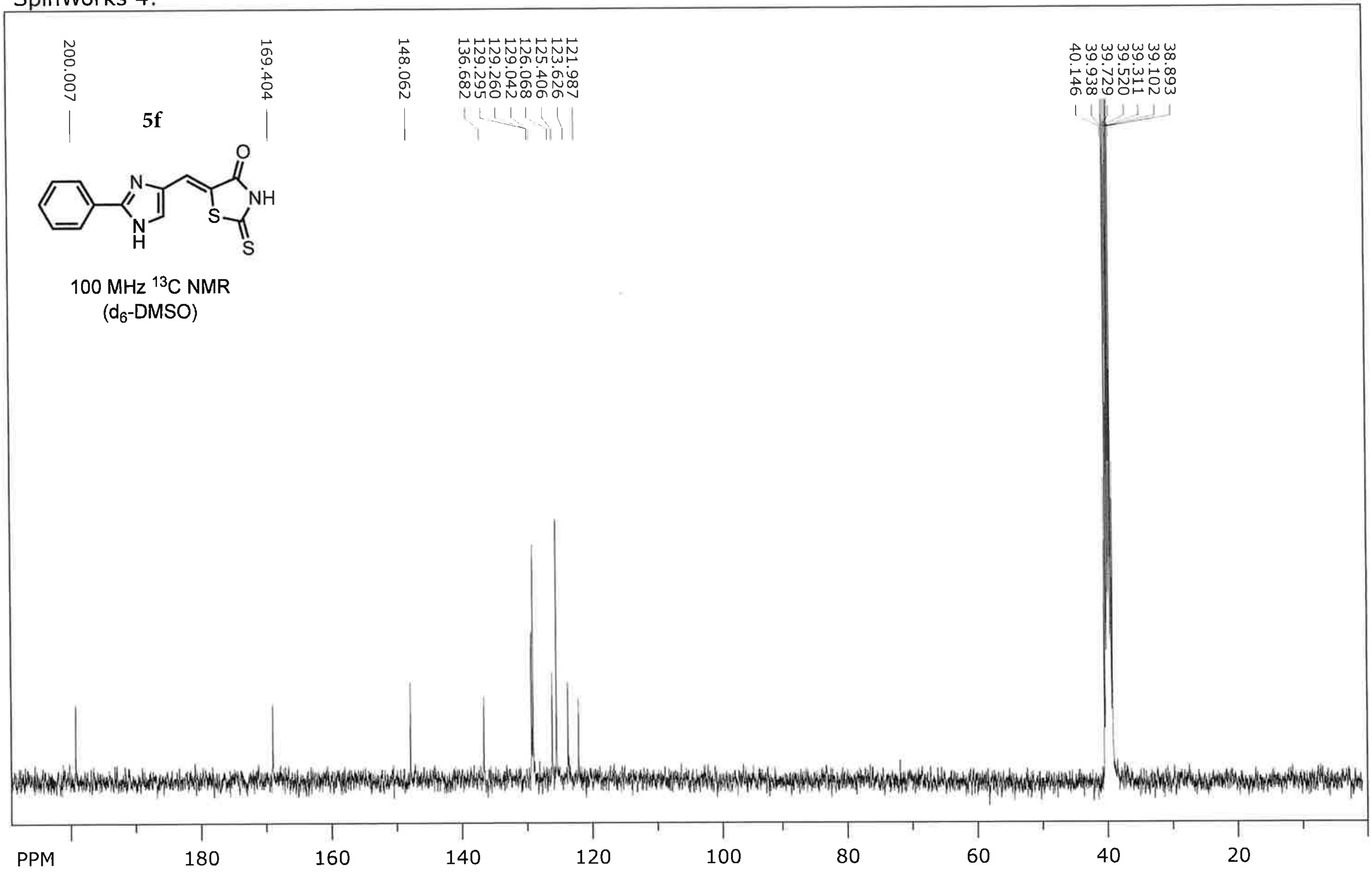

file: ...09\WD405_d6DMSO_2026_05_30.fid\fid block# 1 expt: "s2pul"
transmitter freq.: 100.522537 MHz
time domain size: 50000 points
width: 25000.00 Hz = 248.7004 ppm = 0.500000 Hz/pt
number of scans: 1000

freq. of 0 ppm: 100.511519 MHz
processed size: 65536 complex points
LB: 2.500 GF: 0.0000
Hz/cm: 844.444 ppm/cm: 8.40055

SpinWorks 4:

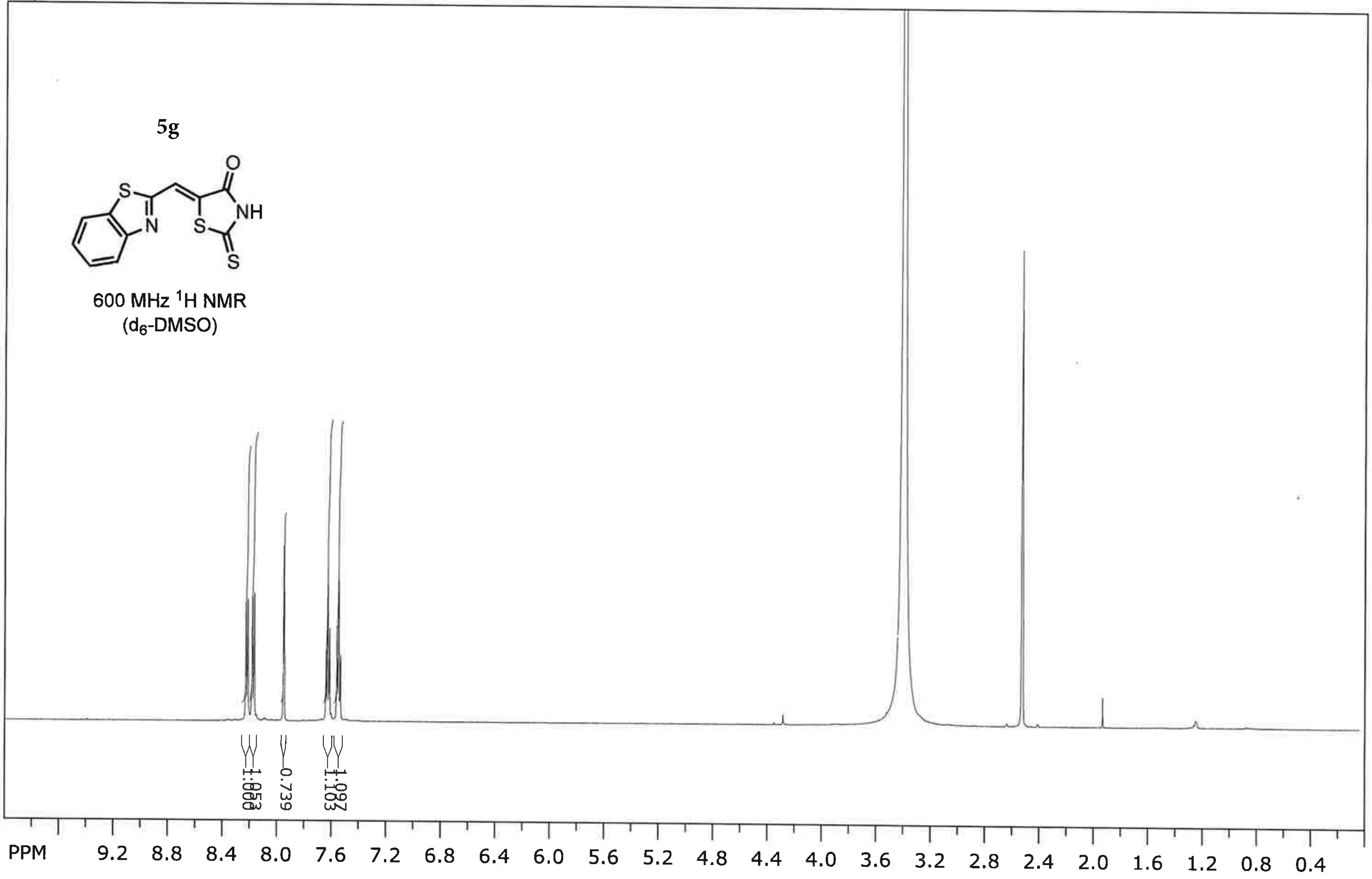

file: ...op\WD398_d6DMSO_2025_12_31.fid\fid block# 1 expt: "s2pul"
transmitter freq.: 599.508636 MHz
time domain size: 32768 points
width: 9615.38 Hz = 16.0388 ppm = 0.293438 Hz/pt
number of scans: 8

freq. of 0 ppm: 599.505039 MHz
processed size: 32768 complex points
LB: 0.500 GF: 0.0000
Hz/cm: 239.744 ppm/cm: 0.39990

SpinWorks 4:

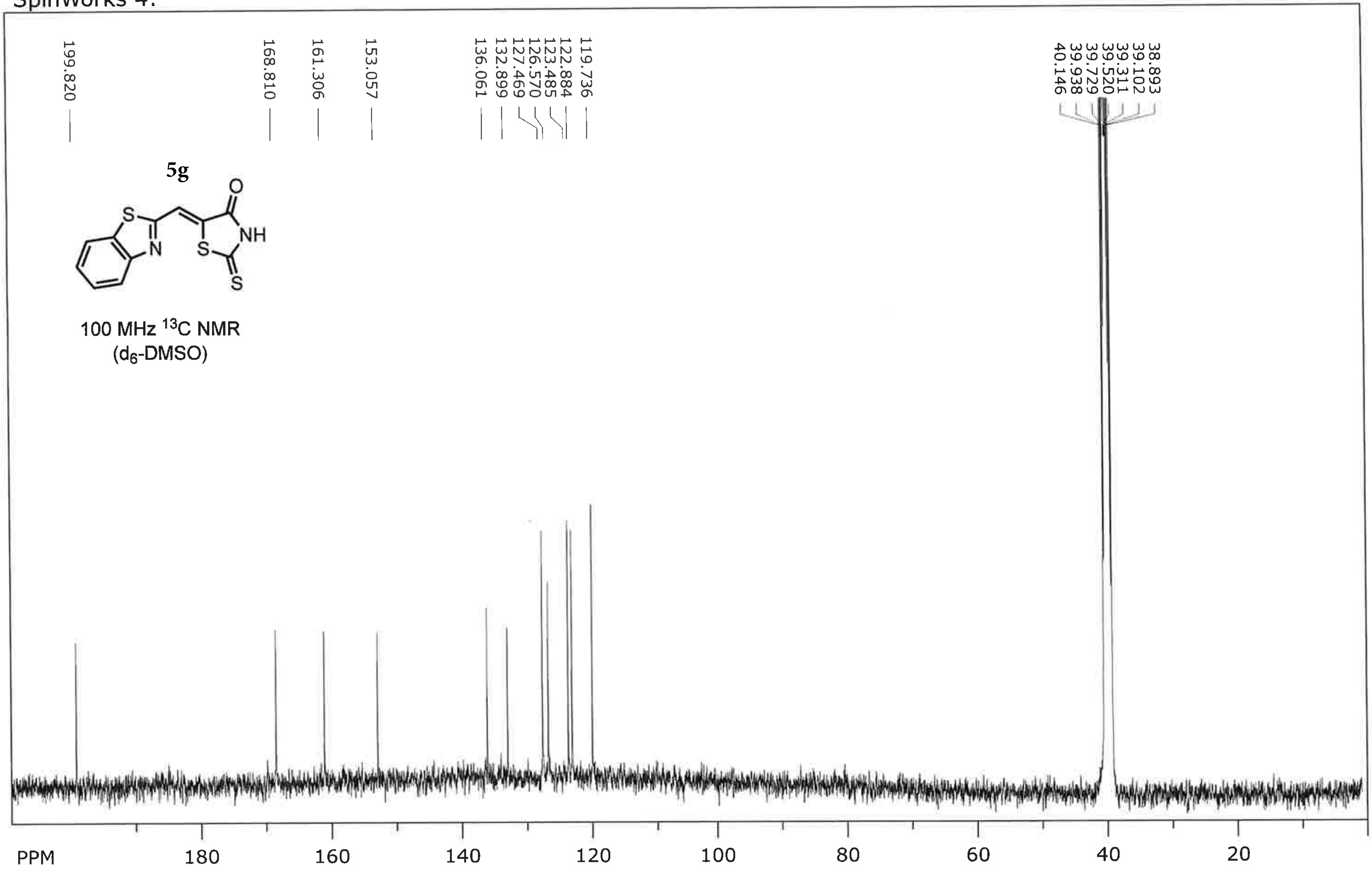

file: ...op\WD398_d6DMSO_2026_08_30.fid\fid block# 1 expt: "s2pul"
transmitter freq.: 100.522325 MHz
time domain size: 50000 points
width: 25000.00 Hz = 248.7010 ppm = 0.500000 Hz/pt
number of scans: 5888

freq. of 0 ppm: 100.511306 MHz
processed size: 65536 complex points
LB: 2.500 GF: 0.0000
Hz/cm: 843.889 ppm/cm: 8.39504

SpinWorks 4:

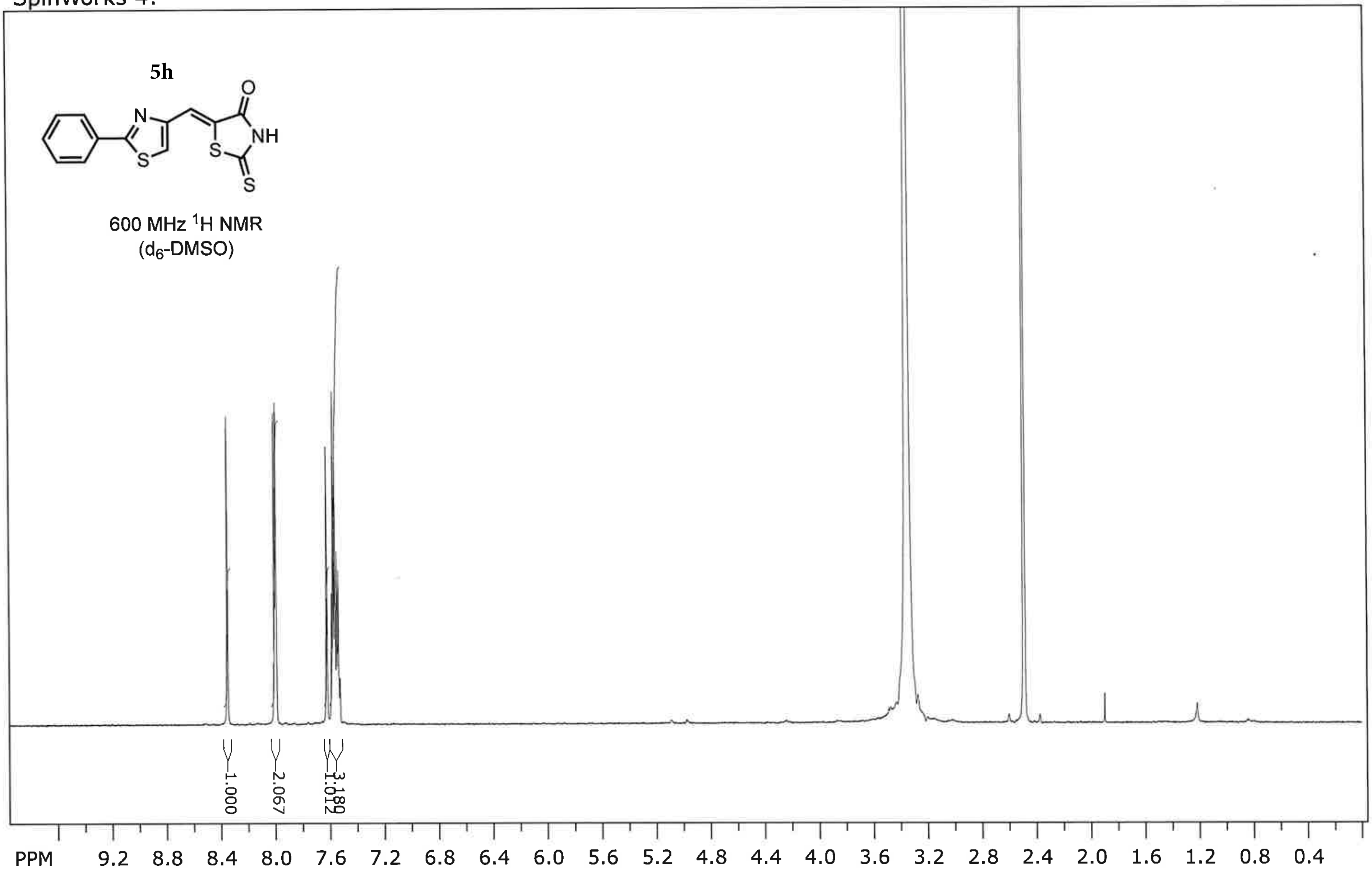

file: ...WD397_1H_d6dmso_2025_12_17.fid\fid block# 1 expt: "s2pul"
transmitter freq.: 599.508636 MHz
time domain size: 32768 points
width: 9615.38 Hz = 16.0388 ppm = 0.293438 Hz/pt
number of scans: 8

freq. of 0 ppm: 599.505036 MHz
processed size: 32768 complex points
LB: 0.500 GF: 0.0000
Hz/cm: 239.744 ppm/cm: 0.39990

SpinWorks 4:

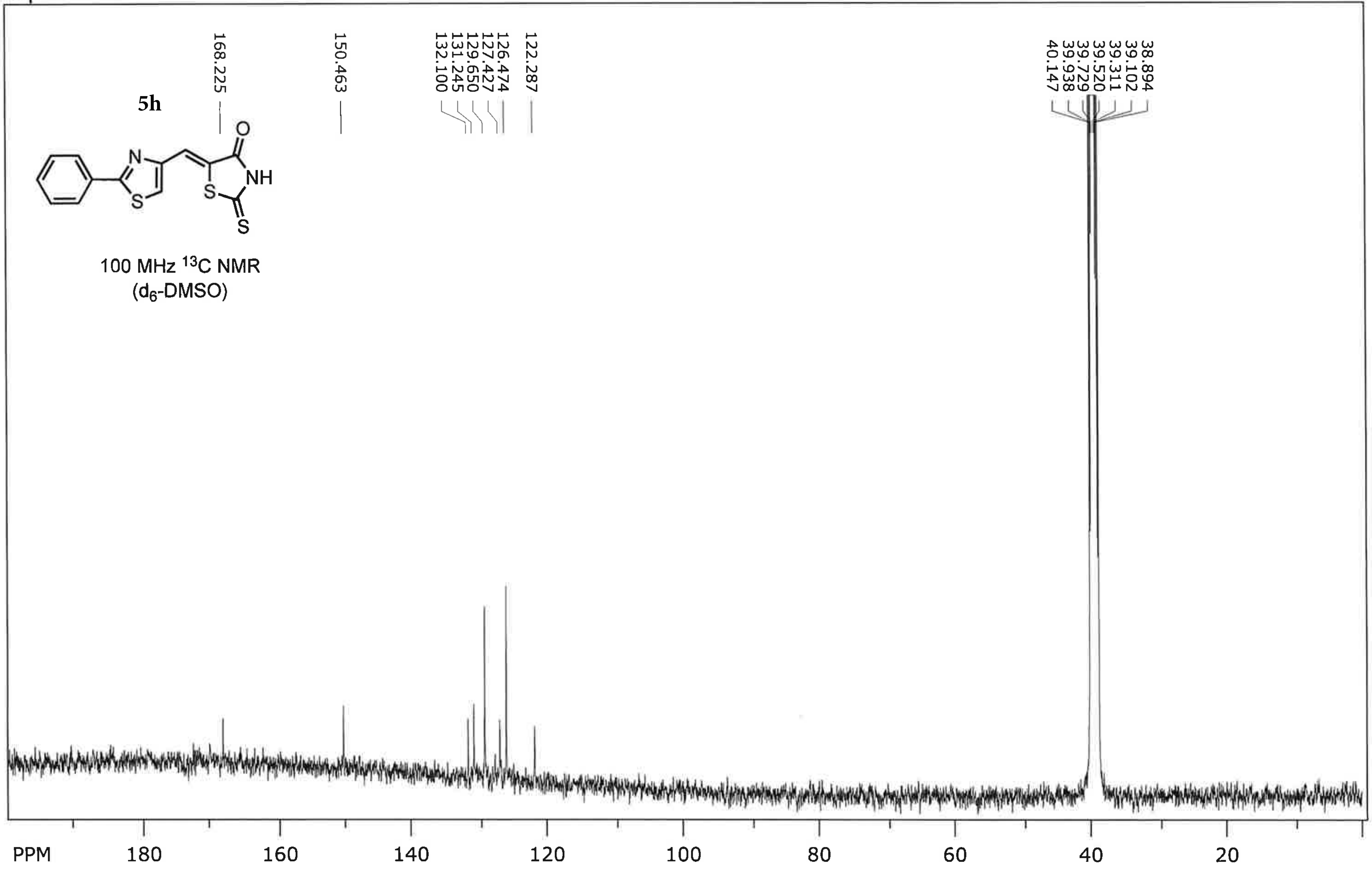

file: ...D397_13C_d6DMSO-2025_12_31.fid\fid block# 1 expt: "s2pul"
transmitter freq.: 100.522893 MHz
time domain size: 50000 points
width: 25000.00 Hz = 248.6996 ppm = 0.500000 Hz/pt
number of scans: 5000

freq. of 0 ppm: 100.511870 MHz
processed size: 65536 complex points
LB: 3.000 GF: 0.0000
Hz/cm: 804.444 ppm/cm: 8.00260

SpinWorks 4:

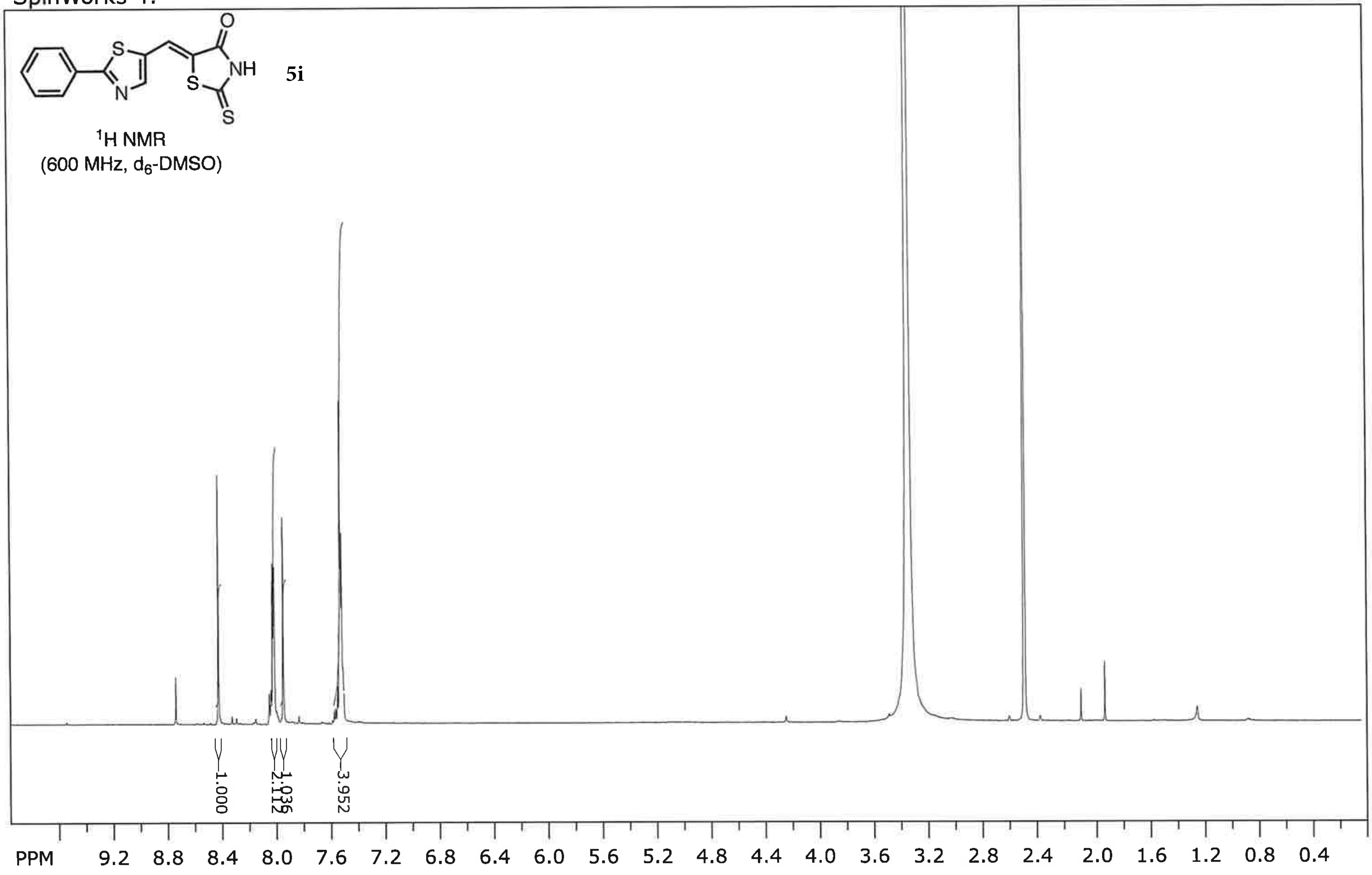

file: ...WD396_1H_d6DMSO_2025_12_10.fid\fid block# 1 expt: "s2pul"
transmitter freq.: 599.508636 MHz
time domain size: 32768 points
width: 9615.38 Hz = 16.0388 ppm = 0.293438 Hz/pt
number of scans: 32

freq. of 0 ppm: 599.505035 MHz
processed size: 32768 complex points
LB: 0.500 GF: 0.0000
Hz/cm: 239.744 ppm/cm: 0.39990

SpinWorks 4:

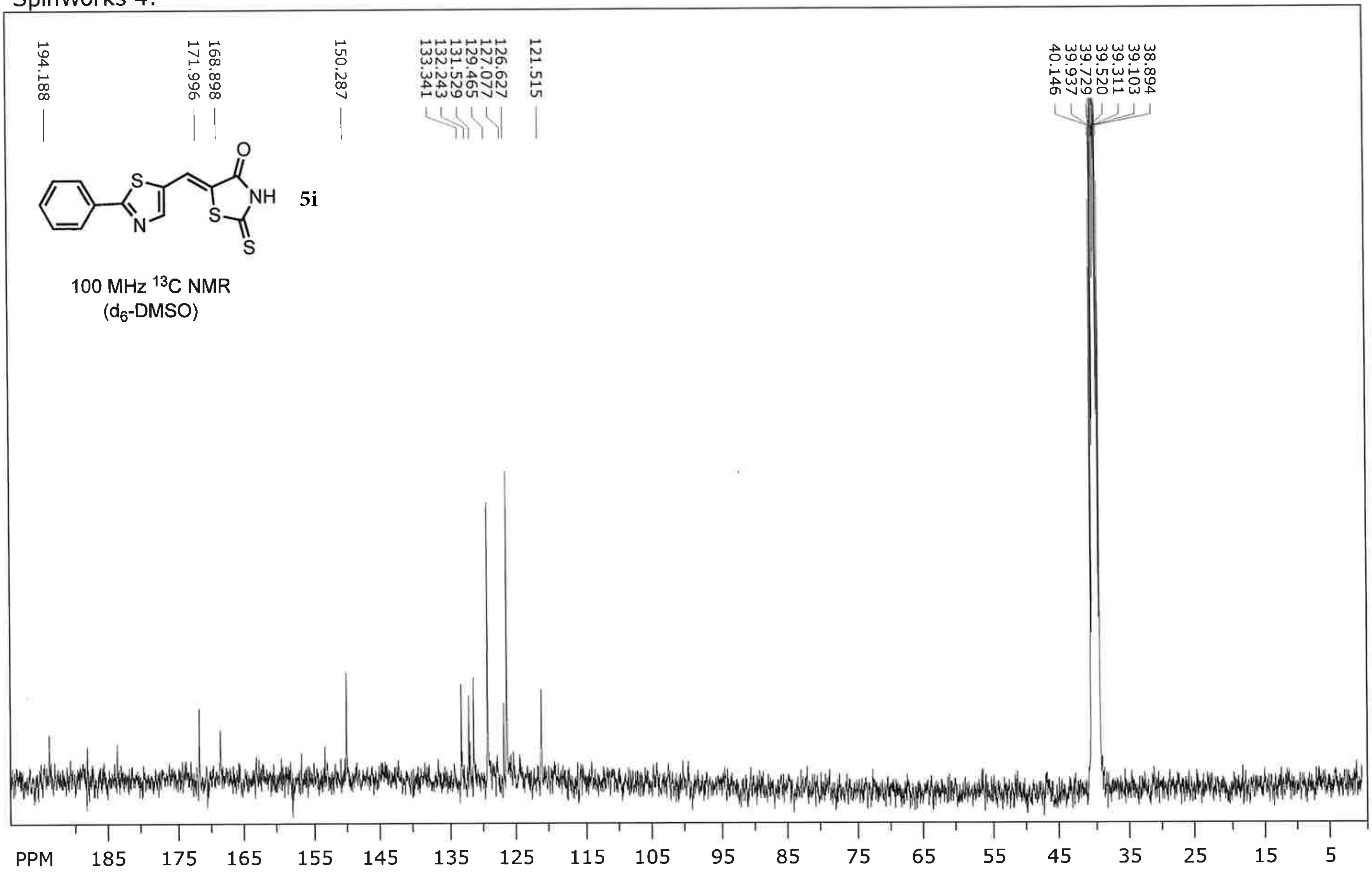

file: ...D396_13C_d6DMSO_2026_05_31.fid\fid block# 1 expt: "s2pul"
transmitter freq.: 100.522537 MHz
time domain size: 50000 points
width: 25000.00 Hz = 248.7004 ppm = 0.500000 Hz/pt
number of scans: 2000

freq. of 0 ppm: 100.511520 MHz
processed size: 65536 complex points
LB: 3.500 GF: 0.0000
Hz/cm: 803.889 ppm/cm: 7.99710

SpinWorks 4:

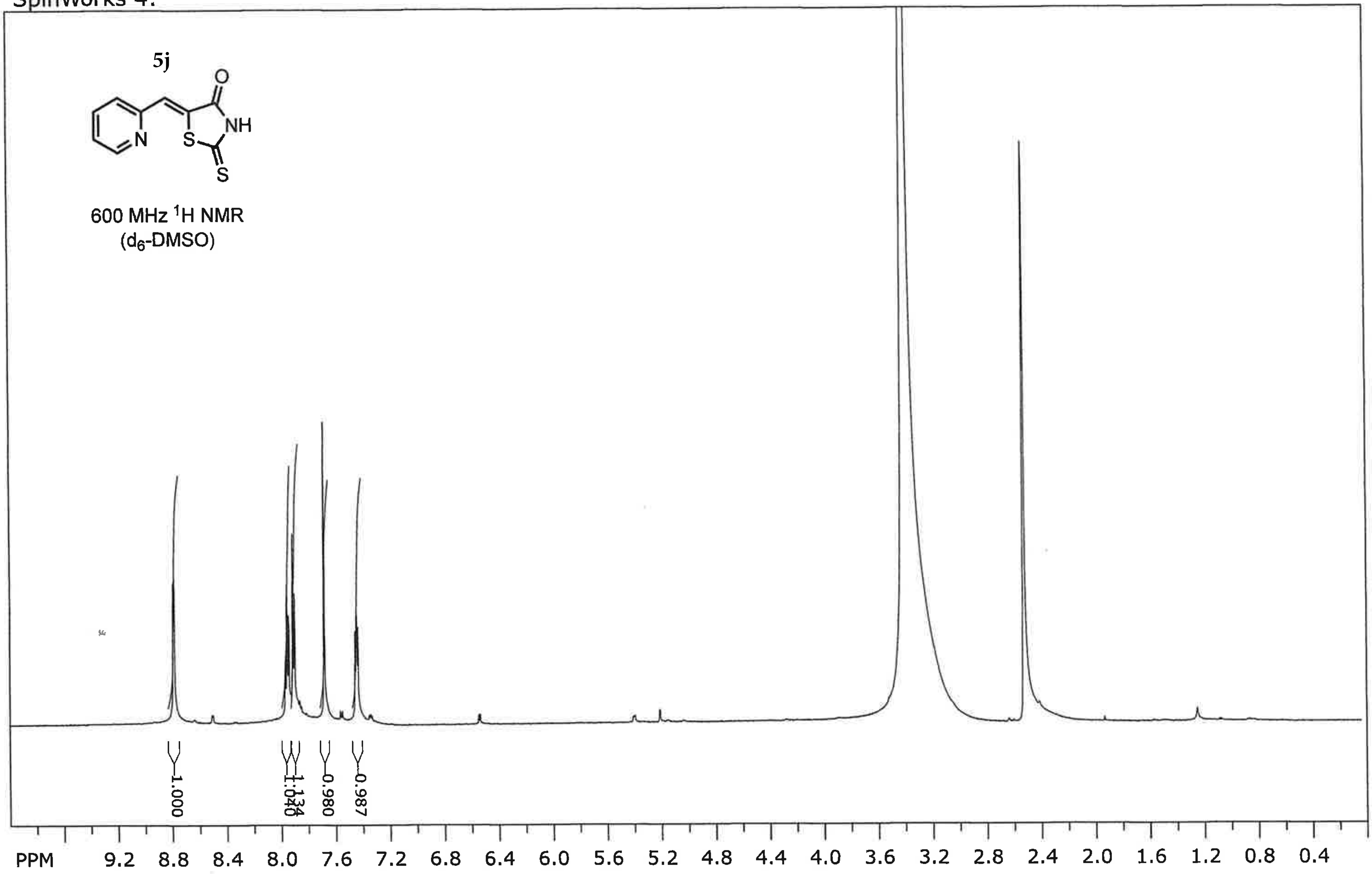

file: ... spectra\KM 060-069\KM-067.fid\fid block# 1 expt: "s2pul"
transmitter freq.: 599.508636 MHz
time domain size: 32768 points
width: 9615.38 Hz = 16.0388 ppm = 0.293438 Hz/pt
number of scans: 64

freq. of 0 ppm: 599.505028 MHz
processed size: 32768 complex points
LB: 0.500 GF: 0.0000
Hz/cm: 239.744 ppm/cm: 0.39990

SpinWorks 4:

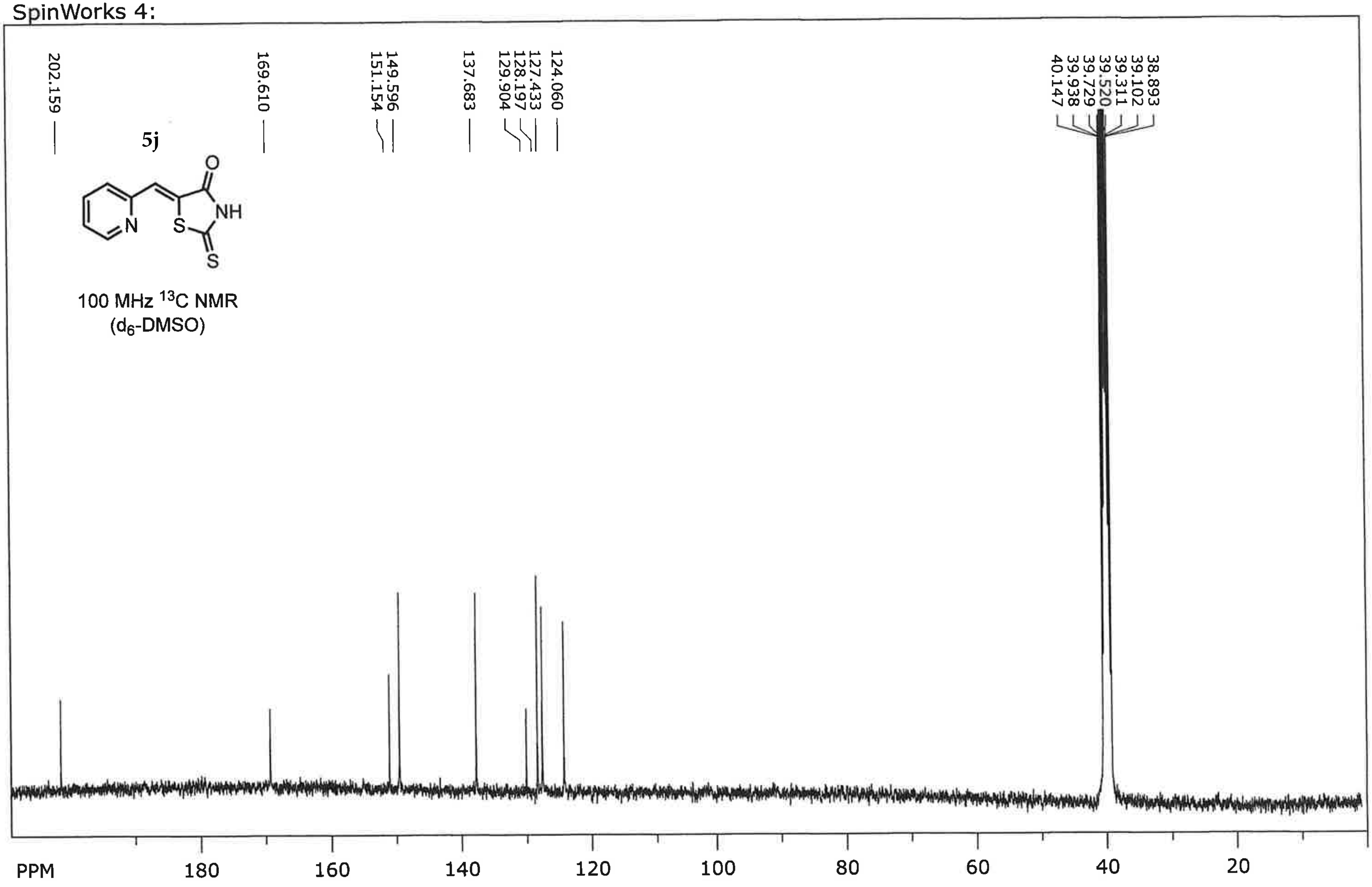

file: ...ectra\KM 060-069\KM-067C13.fid\fid block# 1 expt: "s2pul"
transmitter freq.: 100.522893 MHz
time domain size: 50000 points
width: 25000.00 Hz = 248.6996 ppm = 0.500000 Hz/pt
number of scans: 10000

freq. of 0 ppm: 100.511873 MHz
processed size: 65536 complex points
LB: 2.500 GF: 0.0000
Hz/cm: 843.889 ppm/cm: 8.39499

SpinWorks 4:

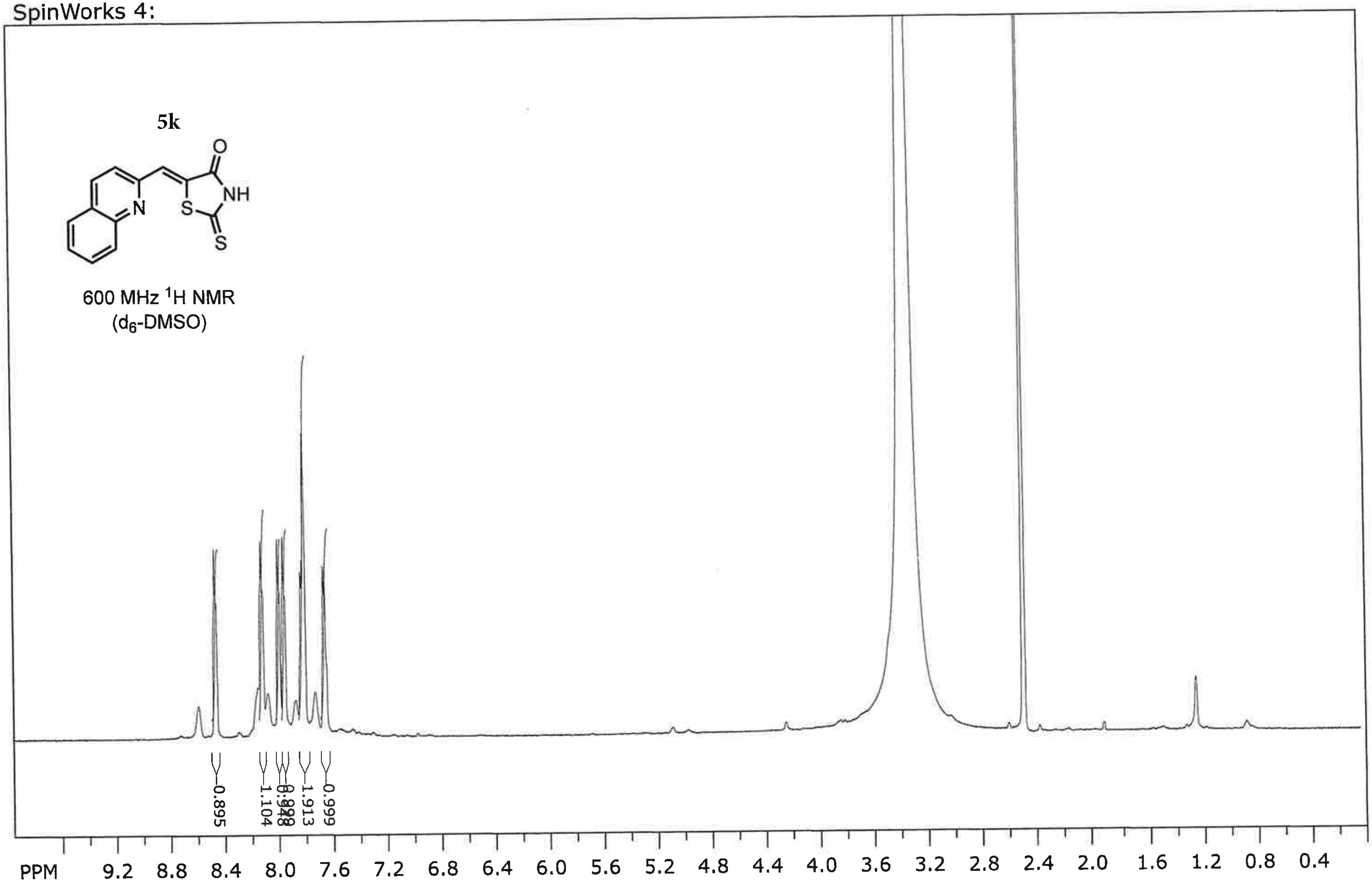

file: ...top\KG28_d6DMSO_2026_02_26.fid\fid block# 1 expt: "s2pul"
transmitter freq.: 599.508636 MHz
time domain size: 32768 points
width: 9615.38 Hz = 16.0388 ppm = 0.293438 Hz/pt
number of scans: 16

freq. of 0 ppm: 599.504875 MHz
processed size: 32768 complex points
LB: 0.500 GF: 0.0000
Hz/cm: 239.744 ppm/cm: 0.39990

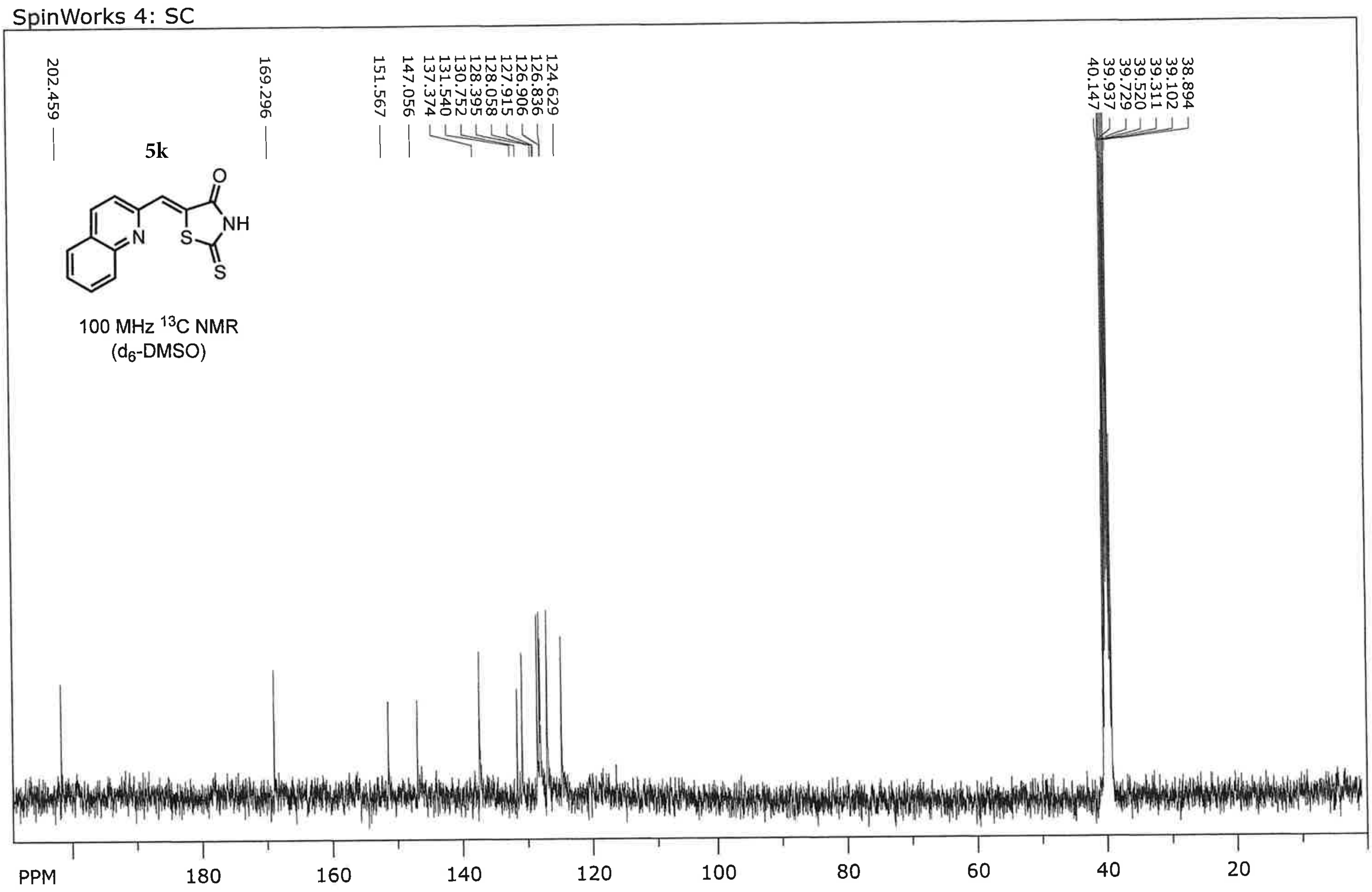

file: ...pectra\KG21-30\KG28-carbon.fid\fid block# 1 expt: "s2pul"
transmitter freq.: 100.523180 MHz
time domain size: 65536 points
width: 25000.00 Hz = 248.6989 ppm = 0.381470 Hz/pt
number of scans: 512

freq. of 0 ppm: 100.512164 MHz
processed size: 65536 complex points
LB: 2.500 GF: 0.0000
Hz/cm: 844.444 ppm/cm: 8.40049

SpinWorks 4:

5l

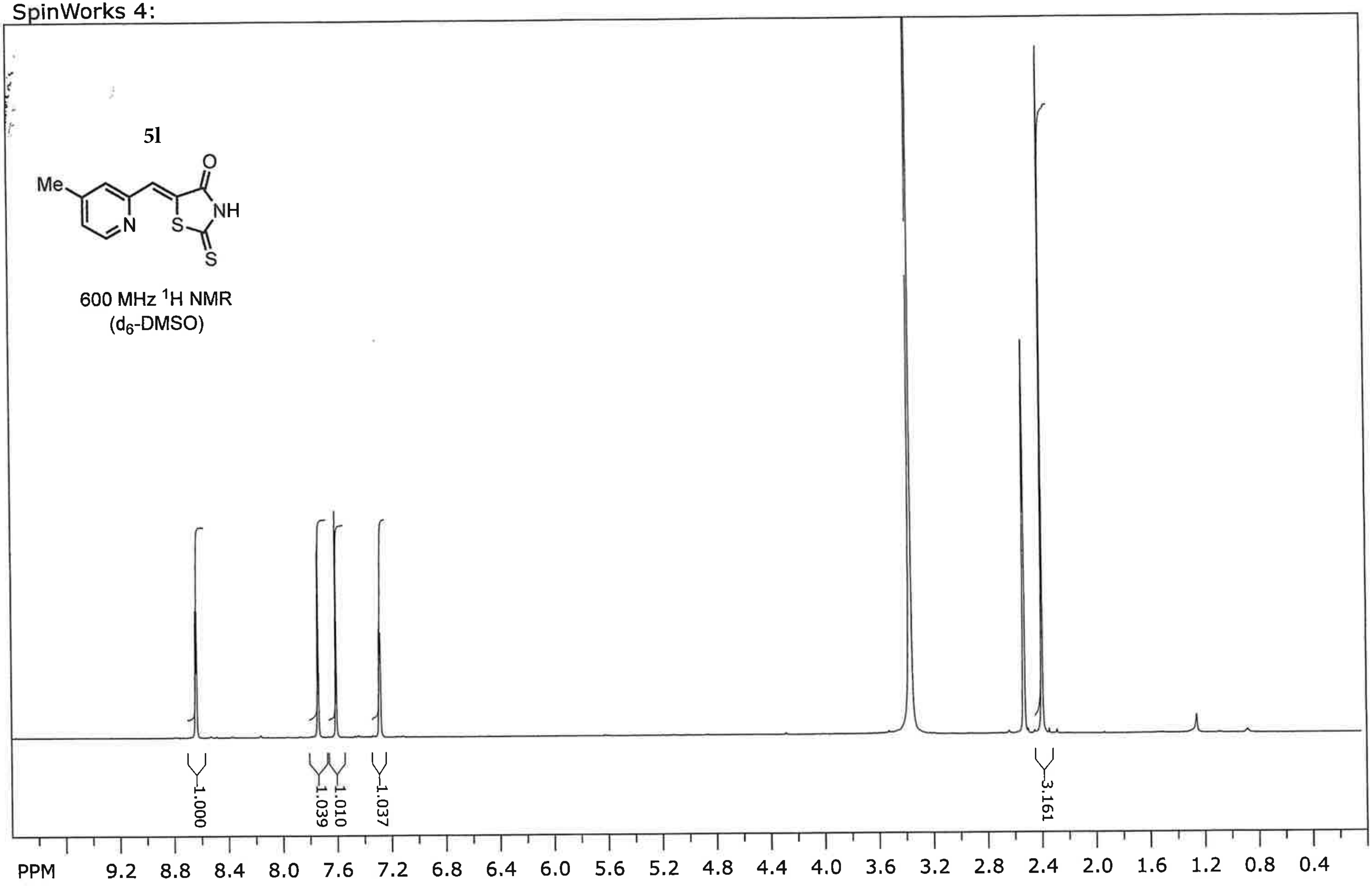

file: ... spectra\KM 060-069\KM-064.fid\fid block# 1 expt: "s2pul"
transmitter freq.: 599.508636 MHz
time domain size: 32768 points
width: 9615.38 Hz = 16.0388 ppm = 0.293438 Hz/pt
number of scans: 32

freq. of 0 ppm: 599.505028 MHz
processed size: 32768 complex points
LB: 0.500 GF: 0.0000
Hz/cm: 239.744 ppm/cm: 0.39990

SpinWorks 4:

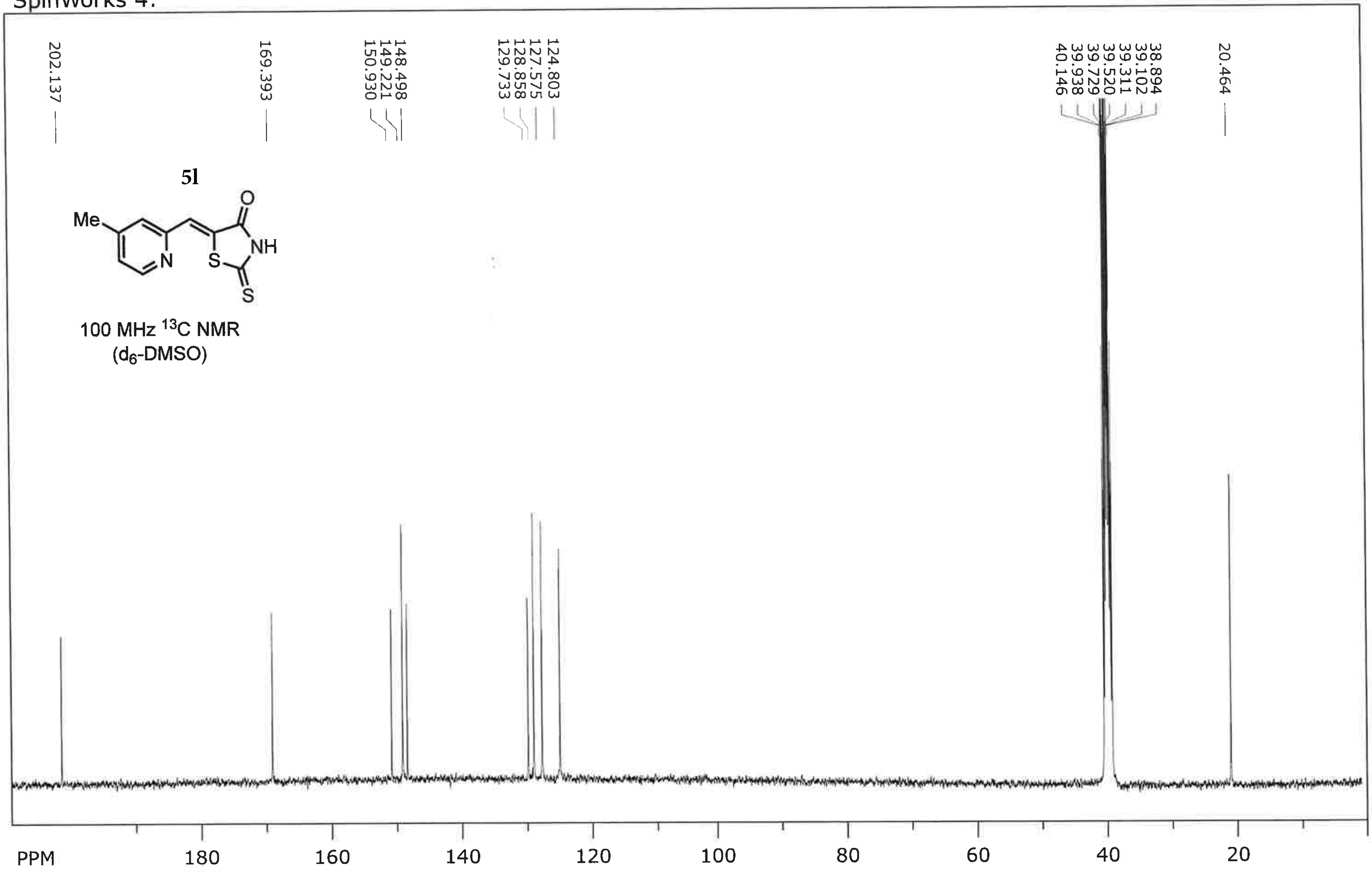

file: ...op\KM064_d6DMSO_2026_08_29.fid\fid block# 1 expt: "s2pul"
transmitter freq.: 100.522325 MHz
time domain size: 50000 points
width: 25000.00 Hz = 248.7010 ppm = 0.500000 Hz/pt
number of scans: 10000

freq. of 0 ppm: 100.511306 MHz
processed size: 65536 complex points
LB: 2.500 GF: 0.0000
Hz/cm: 843.889 ppm/cm: 8.39504

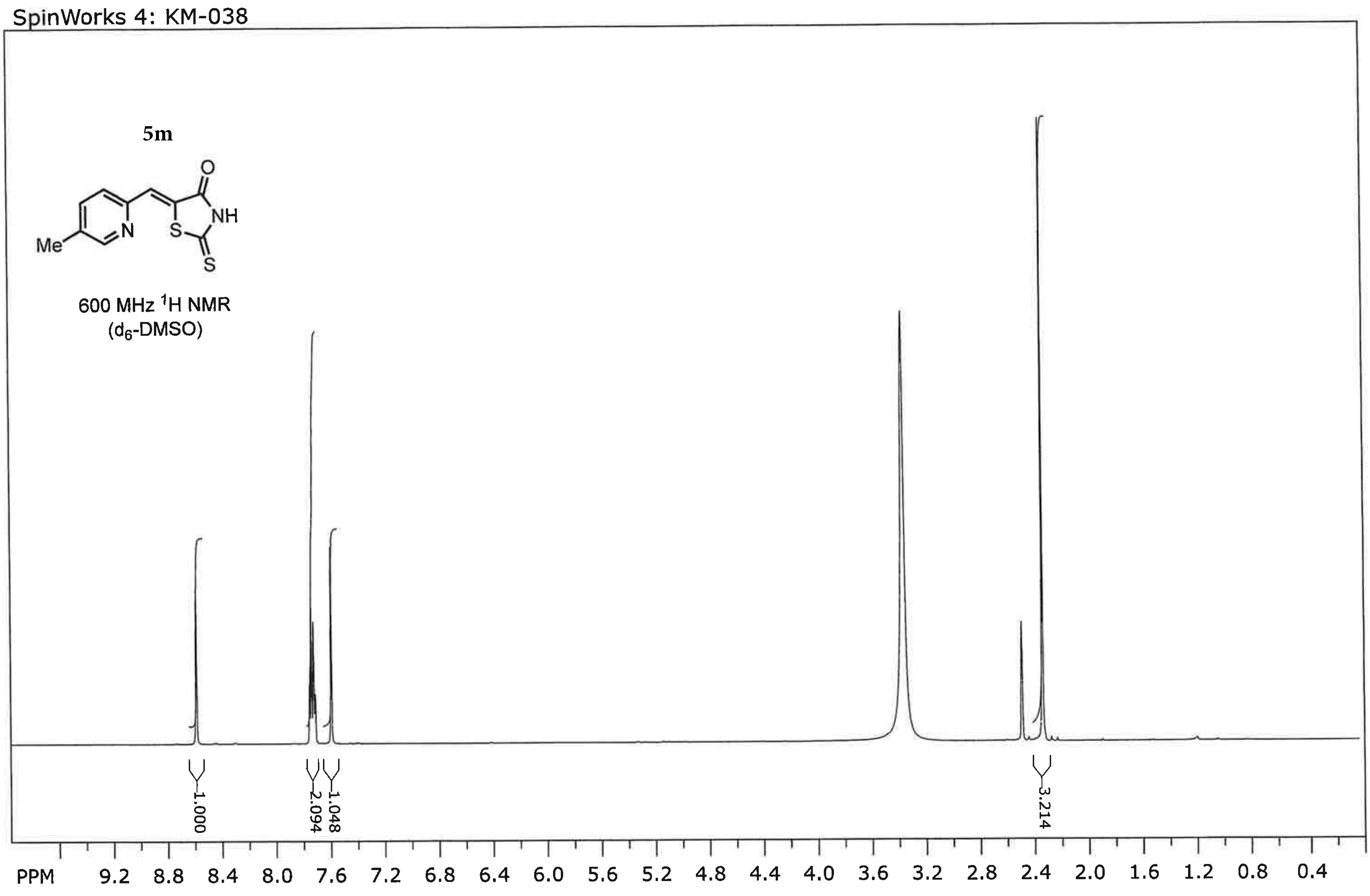

file: ... spectra\KM 050-059\KM-052.fid\fid block# 1 expt: "s2pul"
transmitter freq.: 599.508636 MHz
time domain size: 32768 points
width: 9615.38 Hz = 16.0388 ppm = 0.293438 Hz/pt
number of scans: 32

freq. of 0 ppm: 599.505030 MHz
processed size: 32768 complex points
LB: 0.500 GF: 0.0000
Hz/cm: 239.744 ppm/cm: 0.39990

SpinWorks 4:

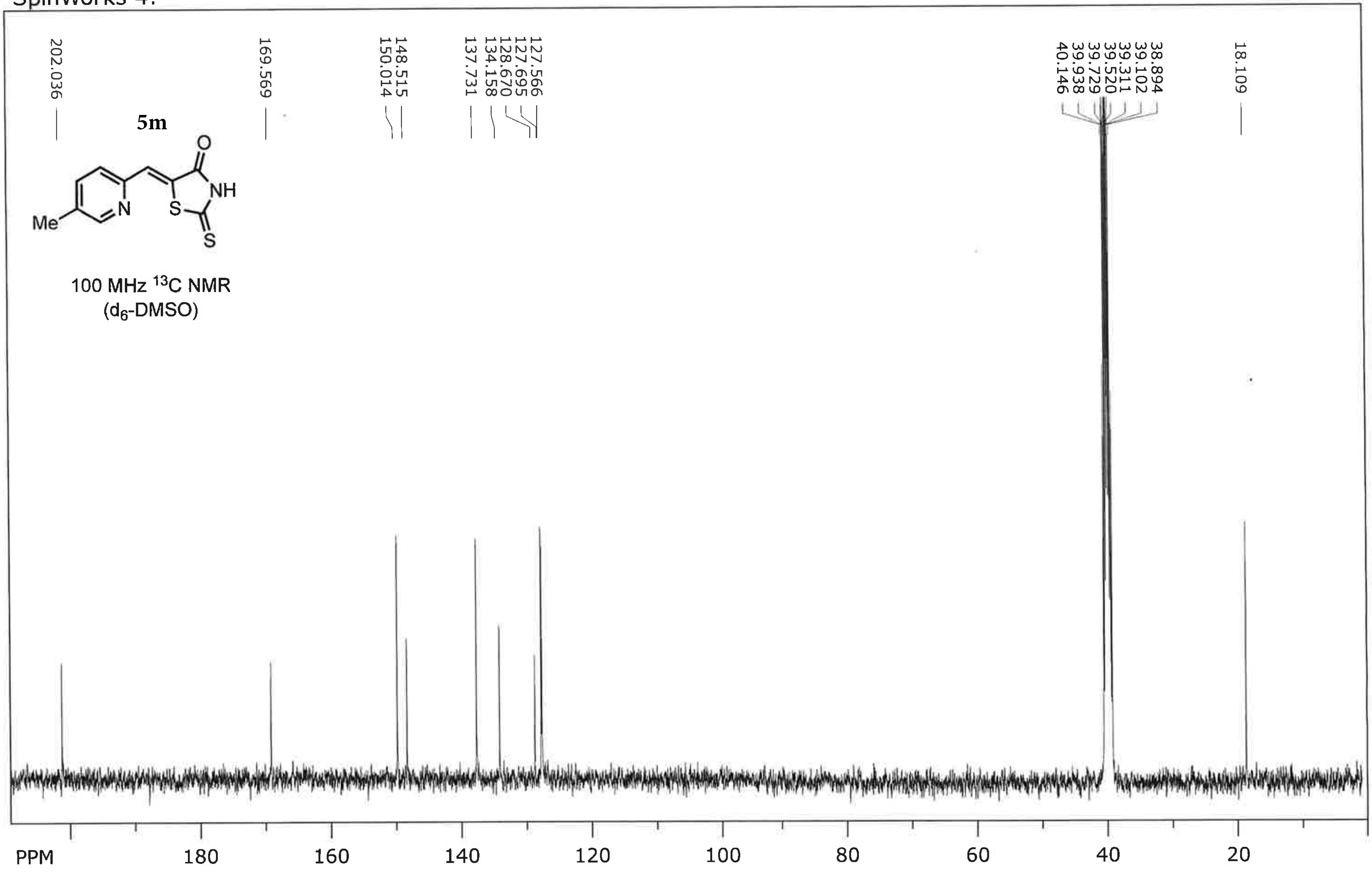

file: ...op\KM052_d6DMSO_2026_05_30.fid\fid block# 1 expt: "s2pul"
transmitter freq.: 100.522537 MHz
time domain size: 50000 points
width: 25000.00 Hz = 248.7004 ppm = 0.500000 Hz/pt
number of scans: 1000

freq. of 0 ppm: 100.511519 MHz
processed size: 65536 complex points
LB: 2.500 GF: 0.0000
Hz/cm: 844.444 ppm/cm: 8.40055

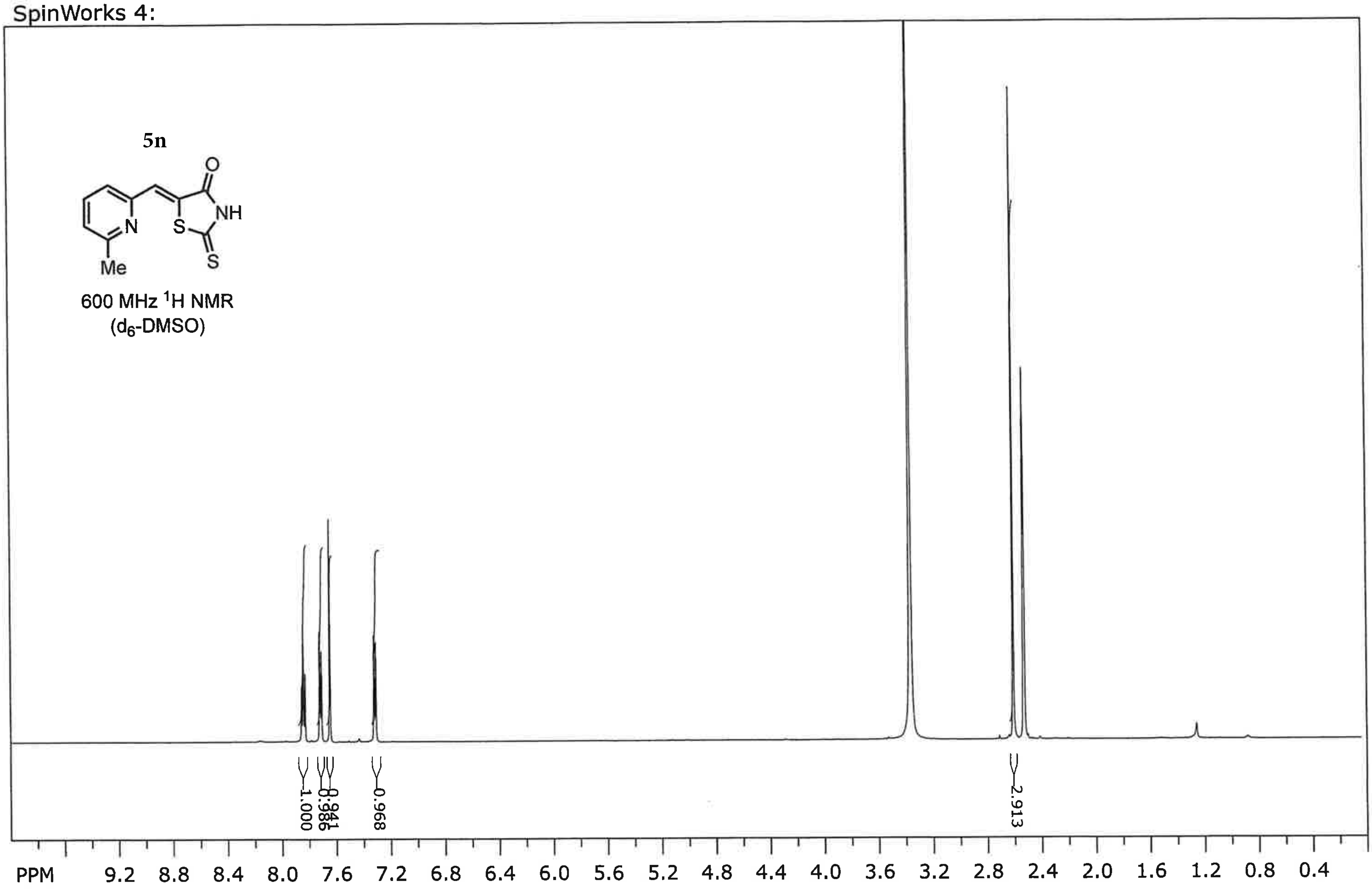

file: ... spectra\KM 060-069\KM-065.fid\fid block# 1 expt: "s2pul"
transmitter freq.: 599.508636 MHz
time domain size: 32768 points
width: 9615.38 Hz = 16.0388 ppm = 0.293438 Hz/pt
number of scans: 32

freq. of 0 ppm: 599.505028 MHz
processed size: 32768 complex points
LB: 0.500 GF: 0.0000
Hz/cm: 239.744 ppm/cm: 0.39990

SpinWorks 4:

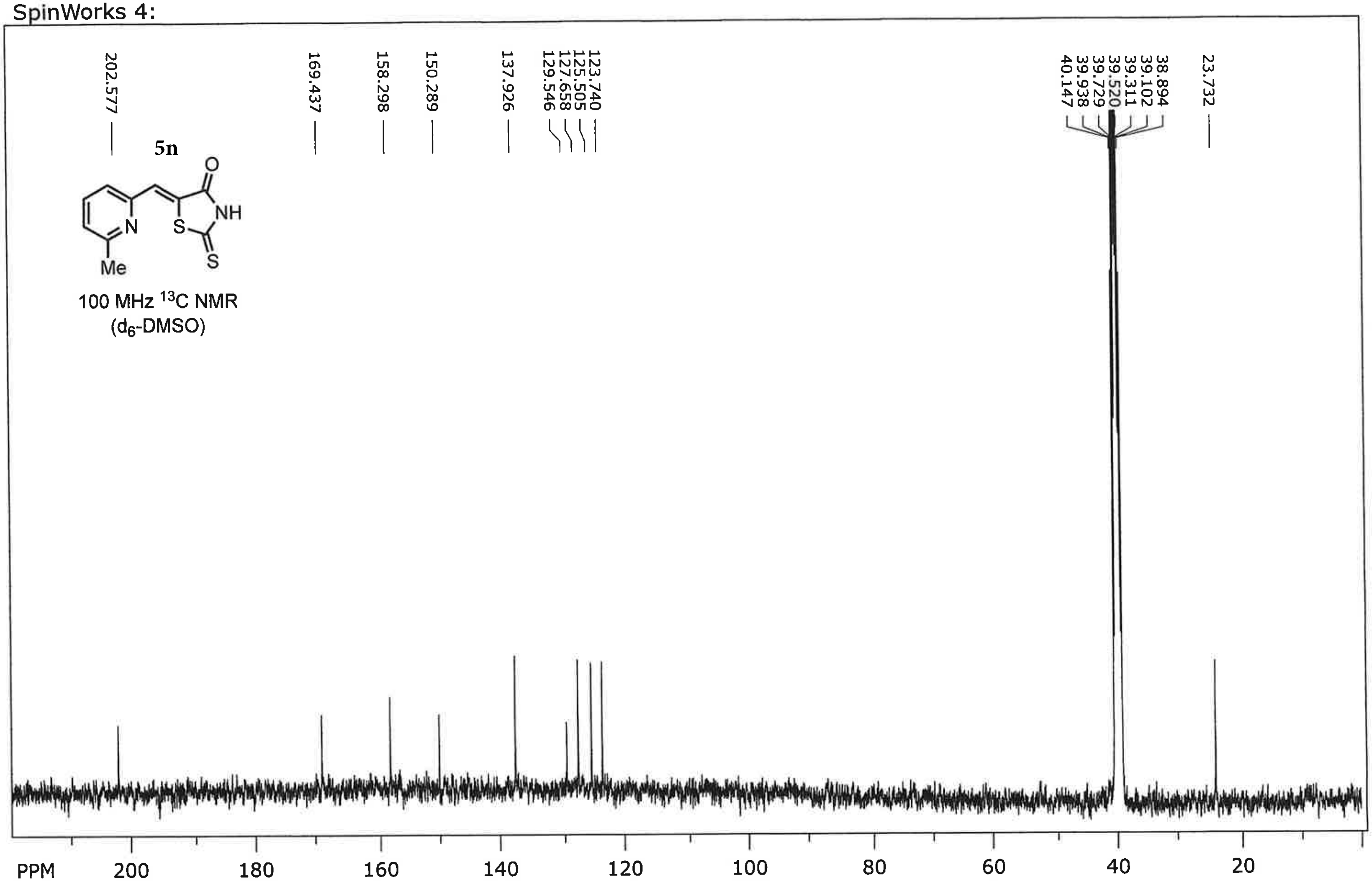

file: ...ectra\KM 060-069\KM-065C13.fid\fid block# 1 expt: "s2pul"
transmitter freq.: 100.523180 MHz
time domain size: 65536 points
width: 25000.00 Hz = 248.6989 ppm = 0.381470 Hz/pt
number of scans: 2000

freq. of 0 ppm: 100.512157 MHz
processed size: 65536 complex points
LB: 3.500 GF: 0.0000
Hz/cm: 885.000 ppm/cm: 8.80394

SpinWorks 4:

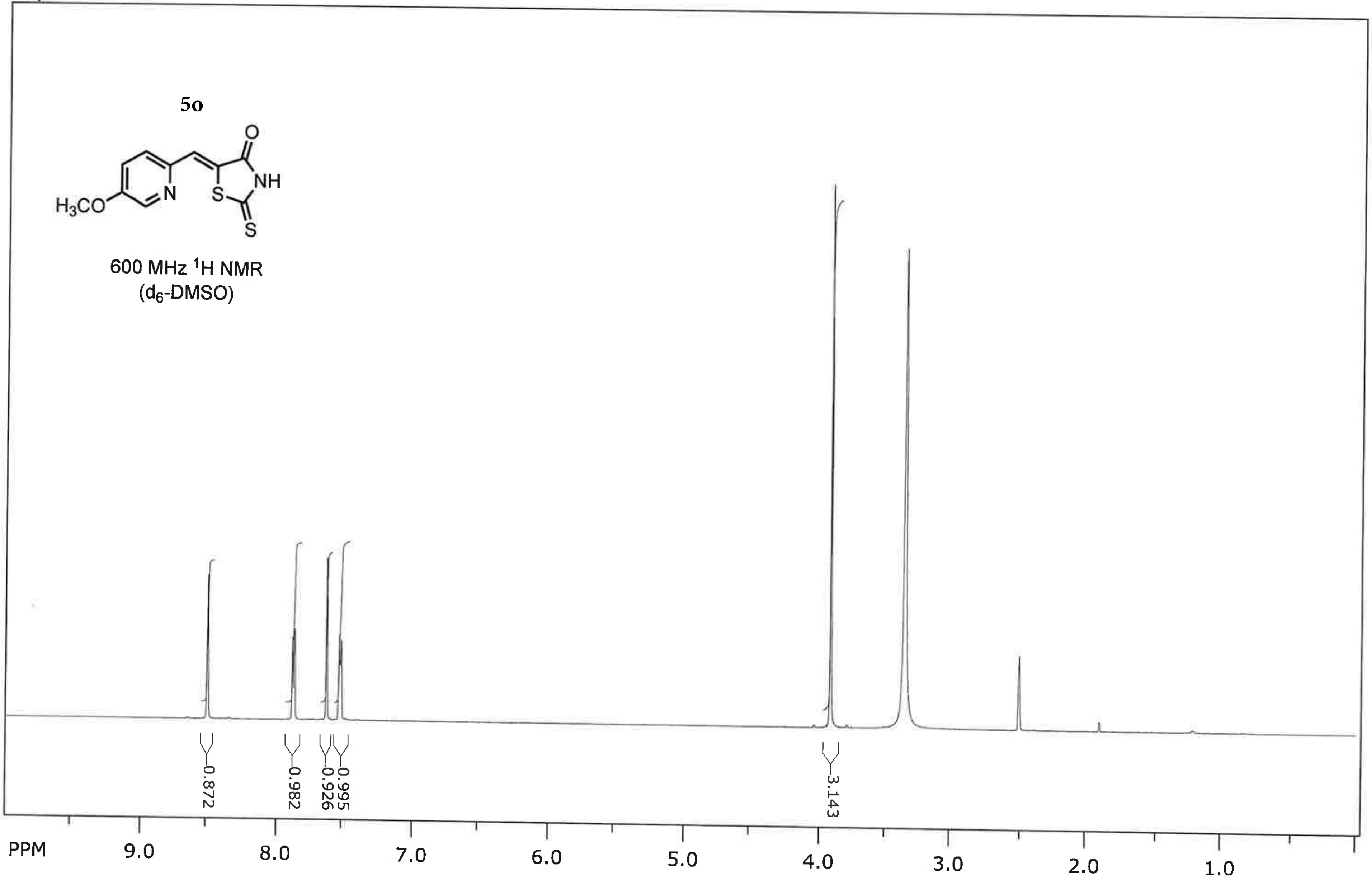

file: ...op\WD400_d6DMSO_2026_04_01.fid\fid block# 1 expt: "s2pul"
transmitter freq.: 599.508636 MHz
time domain size: 32768 points
width: 9615.38 Hz = 16.0388 ppm = 0.293438 Hz/pt
number of scans: 16

freq. of 0 ppm: 599.504909 MHz
processed size: 32768 complex points
LB: 0.500 GF: 0.0000
Hz/cm: 239.957 ppm/cm: 0.40026

SpinWorks 4:

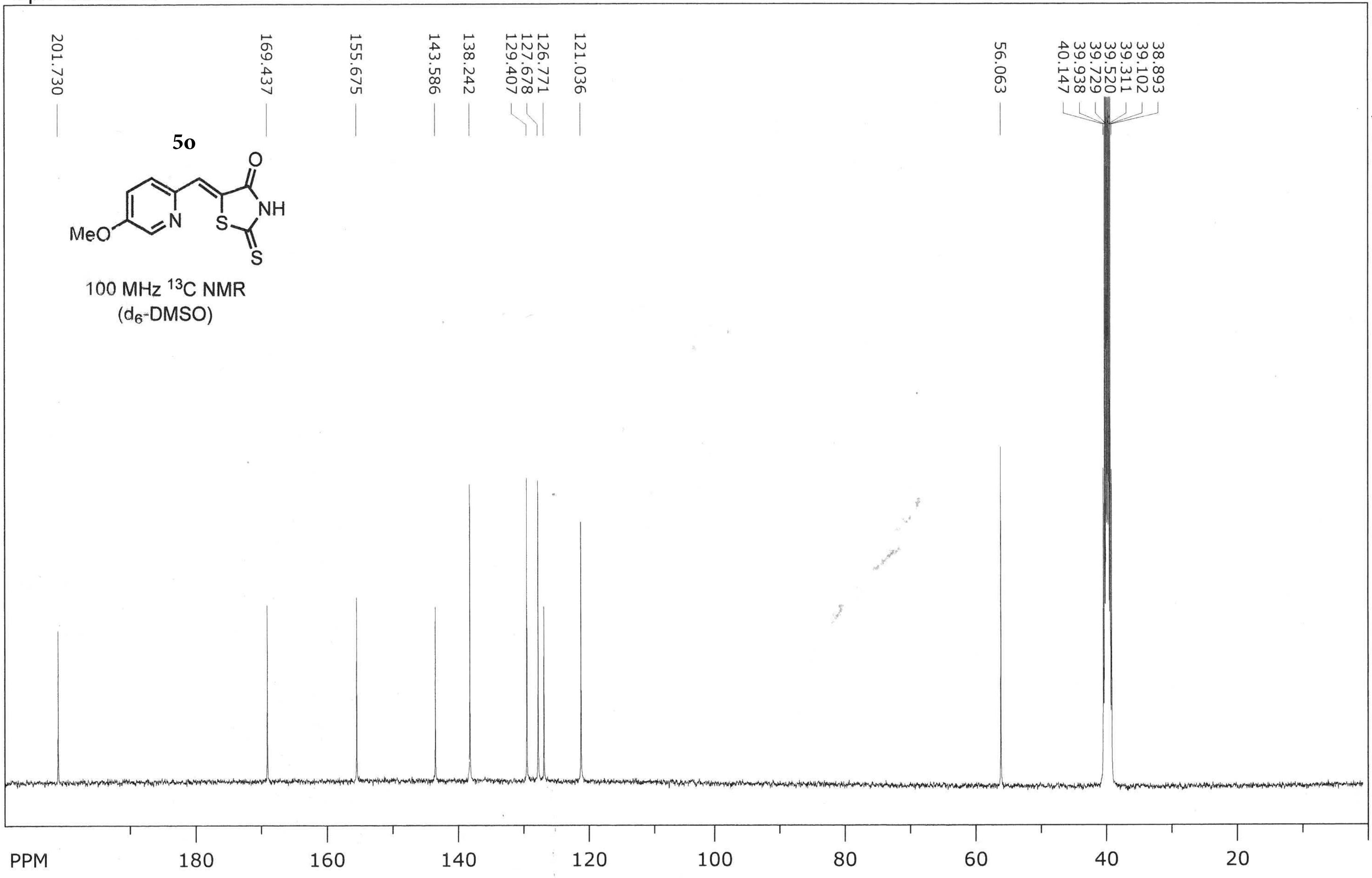

file: ...op\WD400_d6DMSO_2026_09_07.fid\fid block# 1 expt: "s2pul"
transmitter freq.: 100.522325 MHz
time domain size: 50000 points
width: 25000.00 Hz = 248.7010 ppm = 0.500000 Hz/pt
number of scans: 10000

freq. of 0 ppm: 100.511304 MHz
processed size: 65536 complex points
LB: 2.500 GF: 0.0000
Hz/cm: 843.889 ppm/cm: 8.39504

SpinWorks 4:

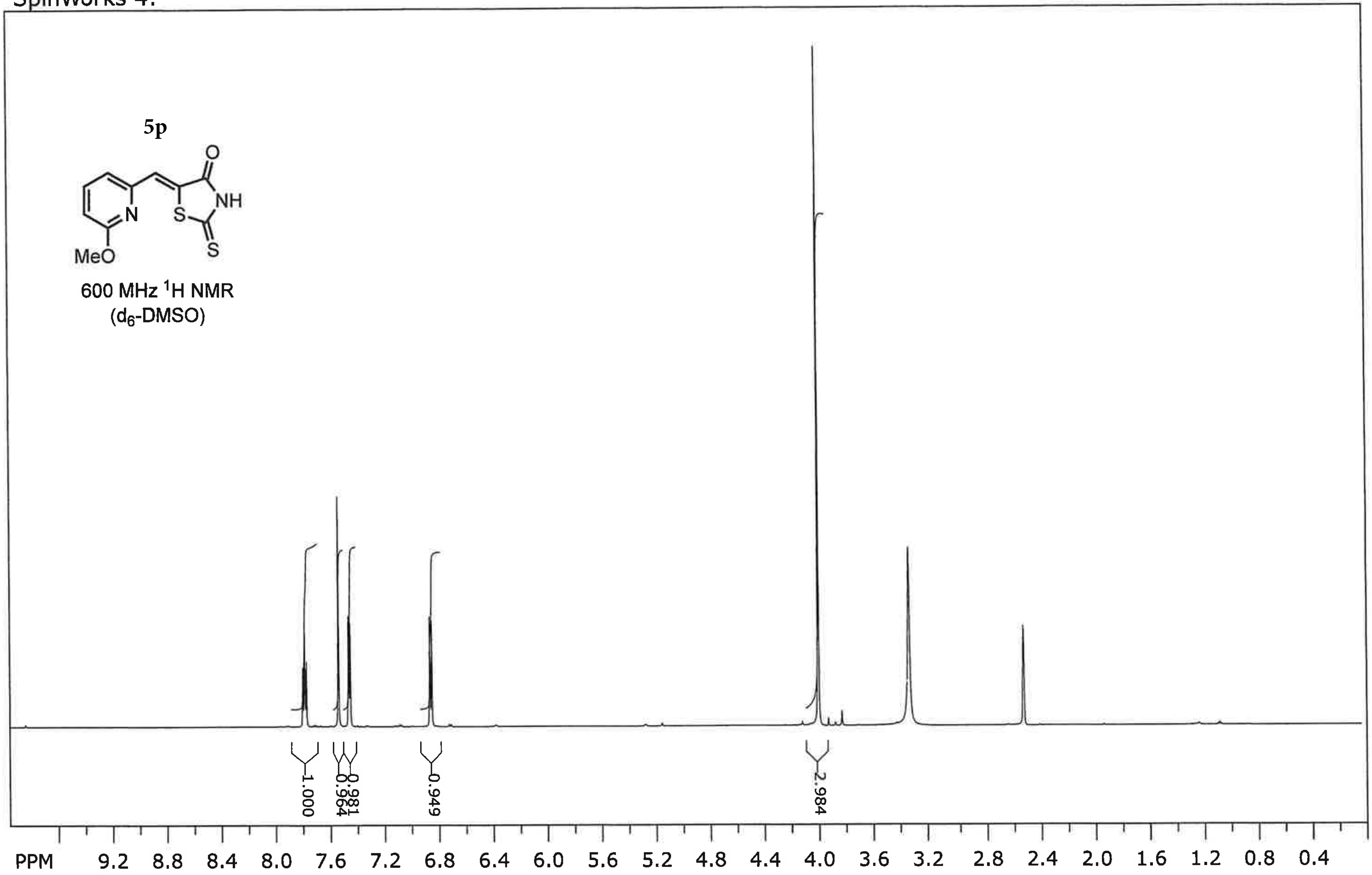

file: ... spectra\KM 060-069\KM-063.fid\fid block# 1 expt: "s2pul"
transmitter freq.: 599.508636 MHz
time domain size: 32768 points
width: 9615.38 Hz = 16.0388 ppm = 0.293438 Hz/pt
number of scans: 32

freq. of 0 ppm: 599.504415 MHz
processed size: 32768 complex points
LB: 0.500 GF: 0.0000
Hz/cm: 239.744 ppm/cm: 0.39990

SpinWorks 4:

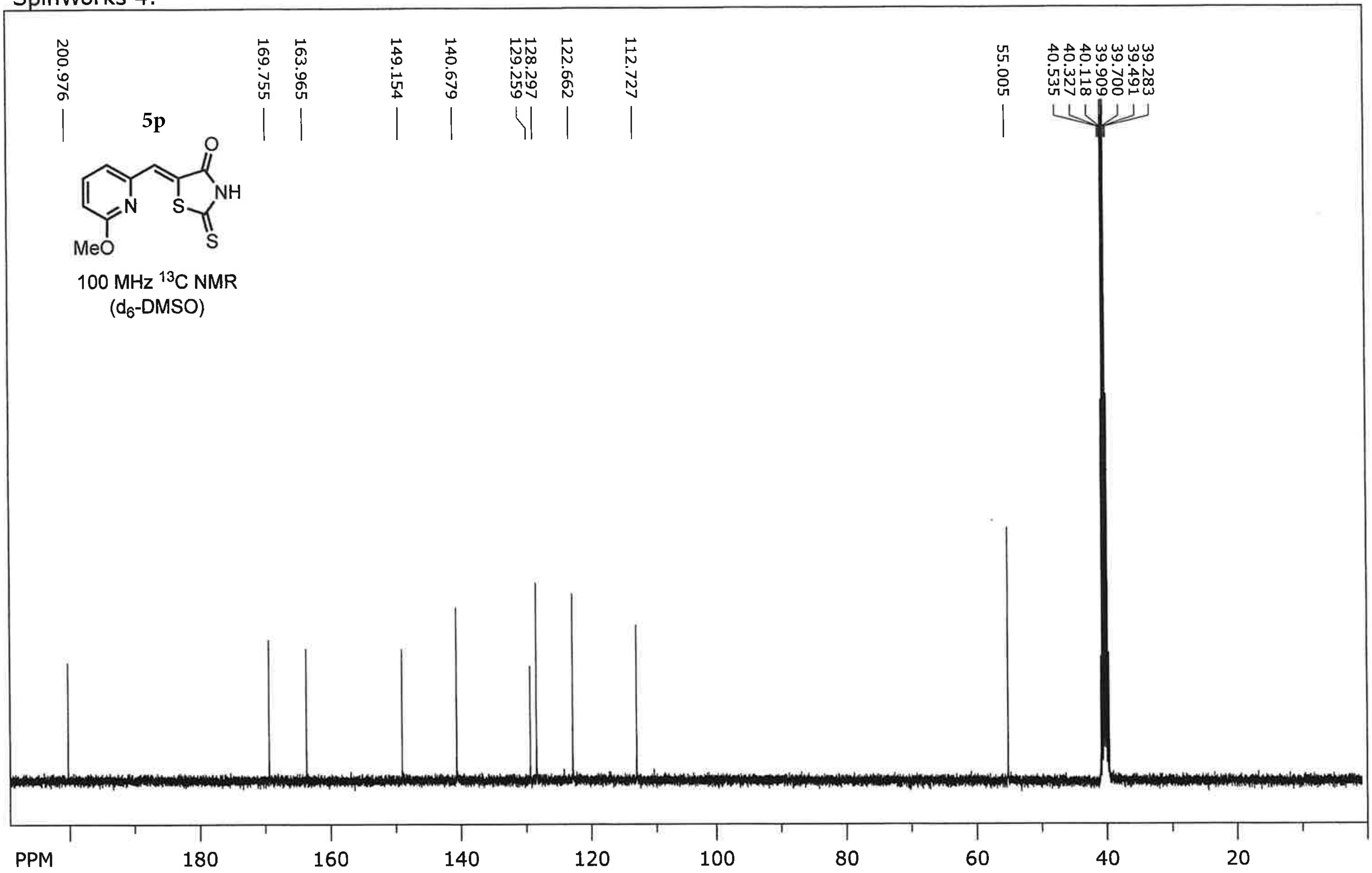

file: ...ectra\KM 060-069\KM-063C13.fid\fid block# 1 expt: "s2pul"
transmitter freq.: 100.523180 MHz
time domain size: 65536 points
width: 25000.00 Hz = 248.6989 ppm = 0.381470 Hz/pt
number of scans: 2000

freq. of 0 ppm: 100.512123 MHz
processed size: 65536 complex points
LB: 0.500 GF: 0.0000
Hz/cm: 844.444 ppm/cm: 8.40049

SpinWorks 4:

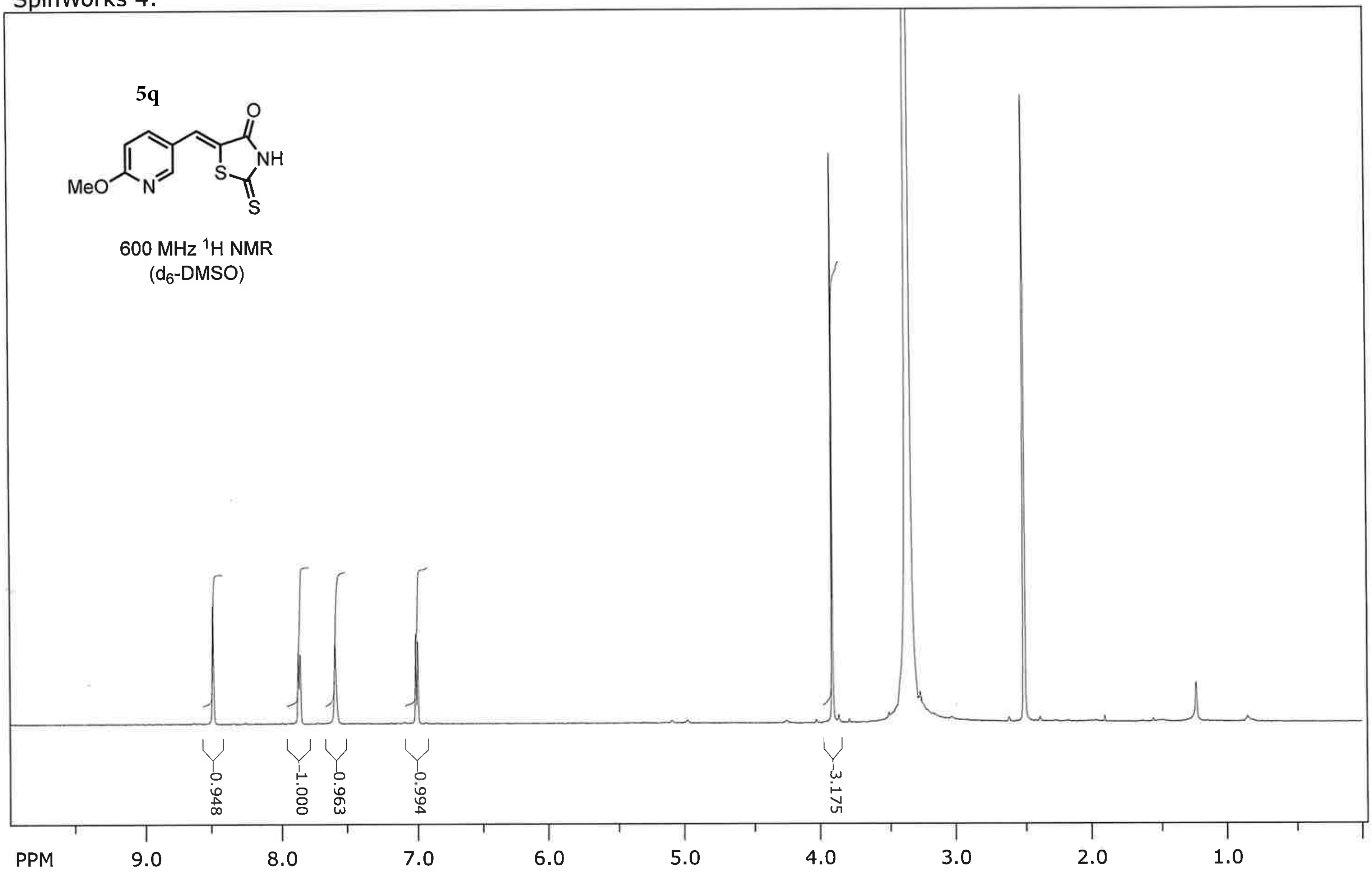

file: ...op\WD401_d6DMSO_2026_04_04.fid\fid block# 1 expt: "s2pul"
transmitter freq.: 599.508636 MHz
time domain size: 32768 points
width: 9615.38 Hz = 16.0388 ppm = 0.293438 Hz/pt
number of scans: 16

freq. of 0 ppm: 599.504974 MHz
processed size: 32768 complex points
LB: 0.500 GF: 0.0000
Hz/cm: 239.957 ppm/cm: 0.40026

SpinWorks 4:

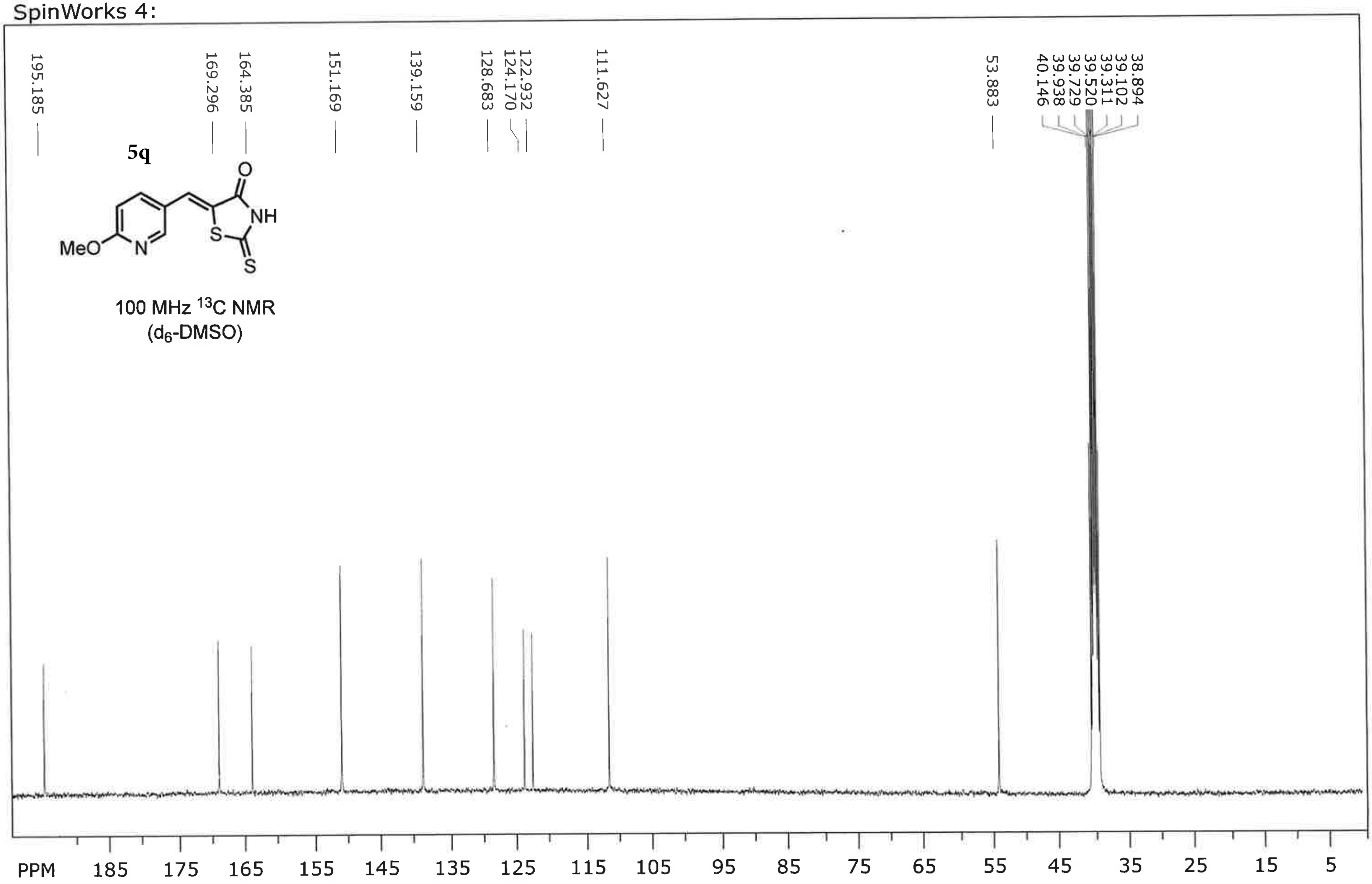

file: ...op\WD401_d6DMSO_2026_08_20.fid\fid block# 1 expt: "s2pul"
transmitter freq.: 100.522325 MHz
time domain size: 50000 points
width: 25000.00 Hz = 248.7010 ppm = 0.500000 Hz/pt
number of scans: 10000

freq. of 0 ppm: 100.511307 MHz
processed size: 65536 complex points
LB: 2.500 GF: 0.0000
Hz/cm: 803.889 ppm/cm: 7.99712

SpinWorks 4:

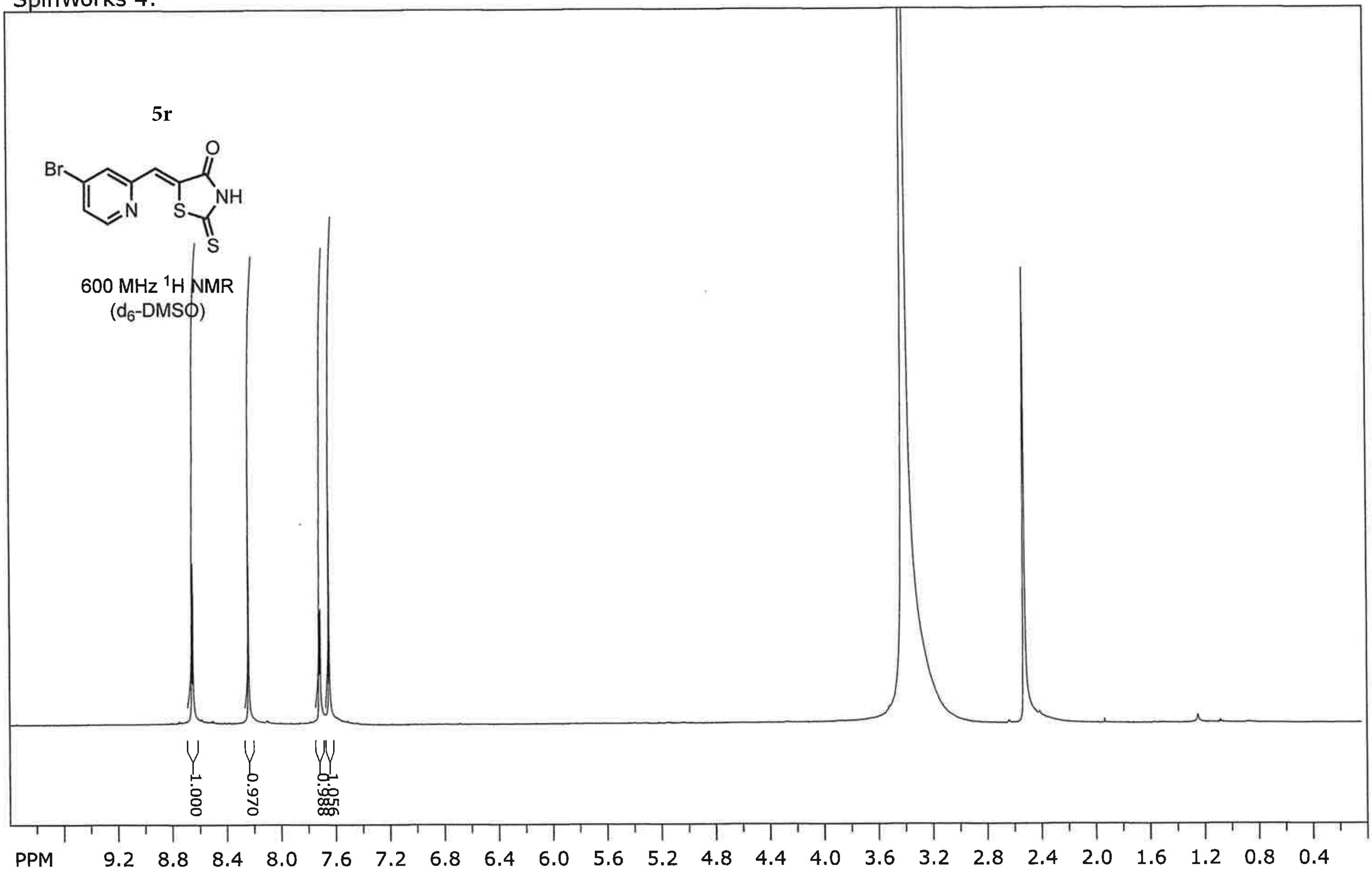

file: ... spectra\KM 060-069\KM-068.fid\fid block# 1 expt: "s2pul"
transmitter freq.: 599.508636 MHz
time domain size: 32768 points
width: 9615.38 Hz = 16.0388 ppm = 0.293438 Hz/pt
number of scans: 64

freq. of 0 ppm: 599.505028 MHz
processed size: 32768 complex points
LB: 0.500 GF: 0.0000
Hz/cm: 239.744 ppm/cm: 0.39990

SpinWorks 4:

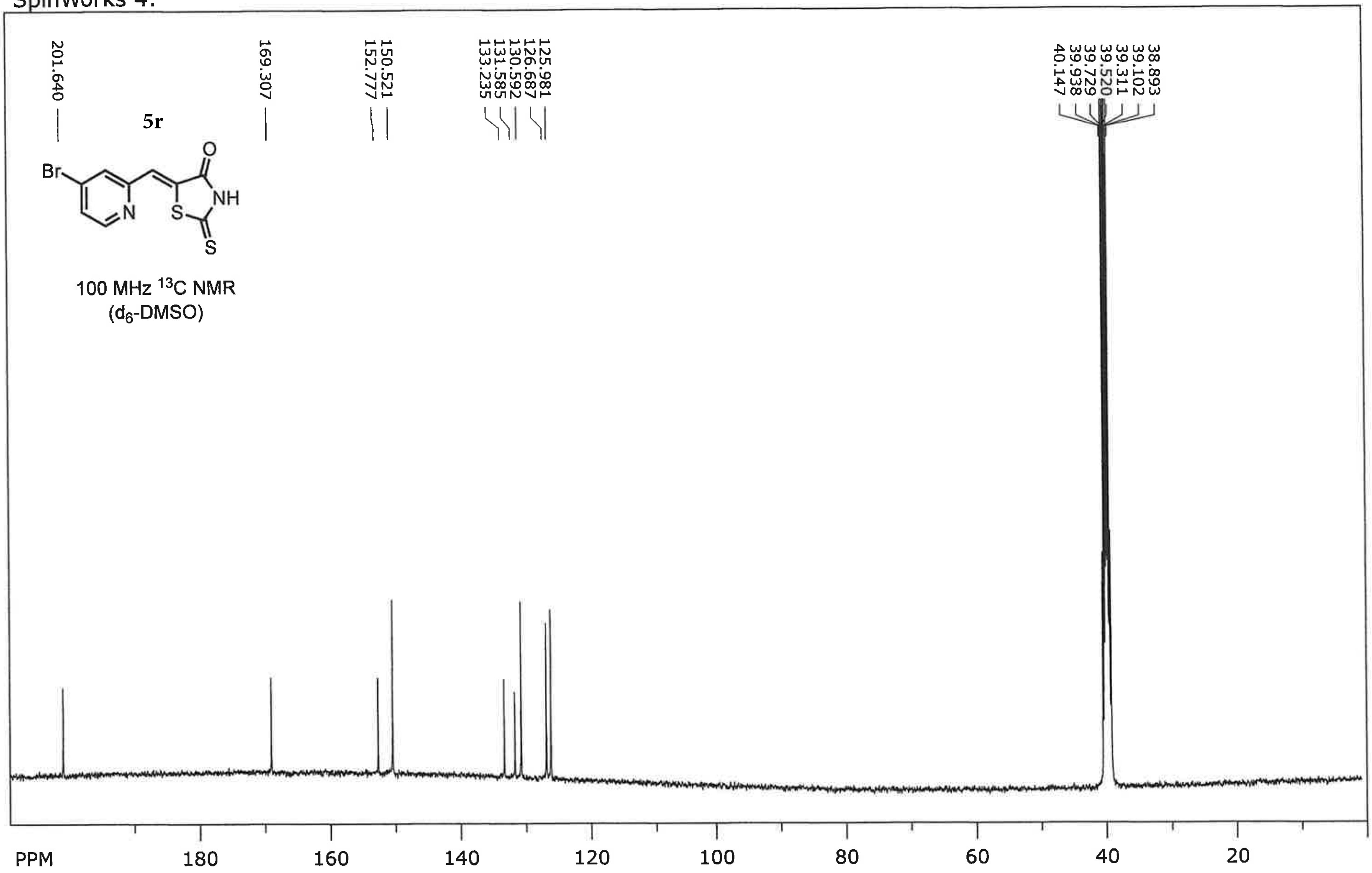

file: ...ectra\KM 060-069\KM-068C13.fid\fid block# 1 expt: "s2pul"
transmitter freq.: 100.522893 MHz
time domain size: 50000 points
width: 25000.00 Hz = 248.6996 ppm = 0.500000 Hz/pt
number of scans: 10000

freq. of 0 ppm: 100.511874 MHz
processed size: 65536 complex points
LB: 2.500 GF: 0.0000
Hz/cm: 843.889 ppm/cm: 8.39499

SpinWorks 4: KM-041

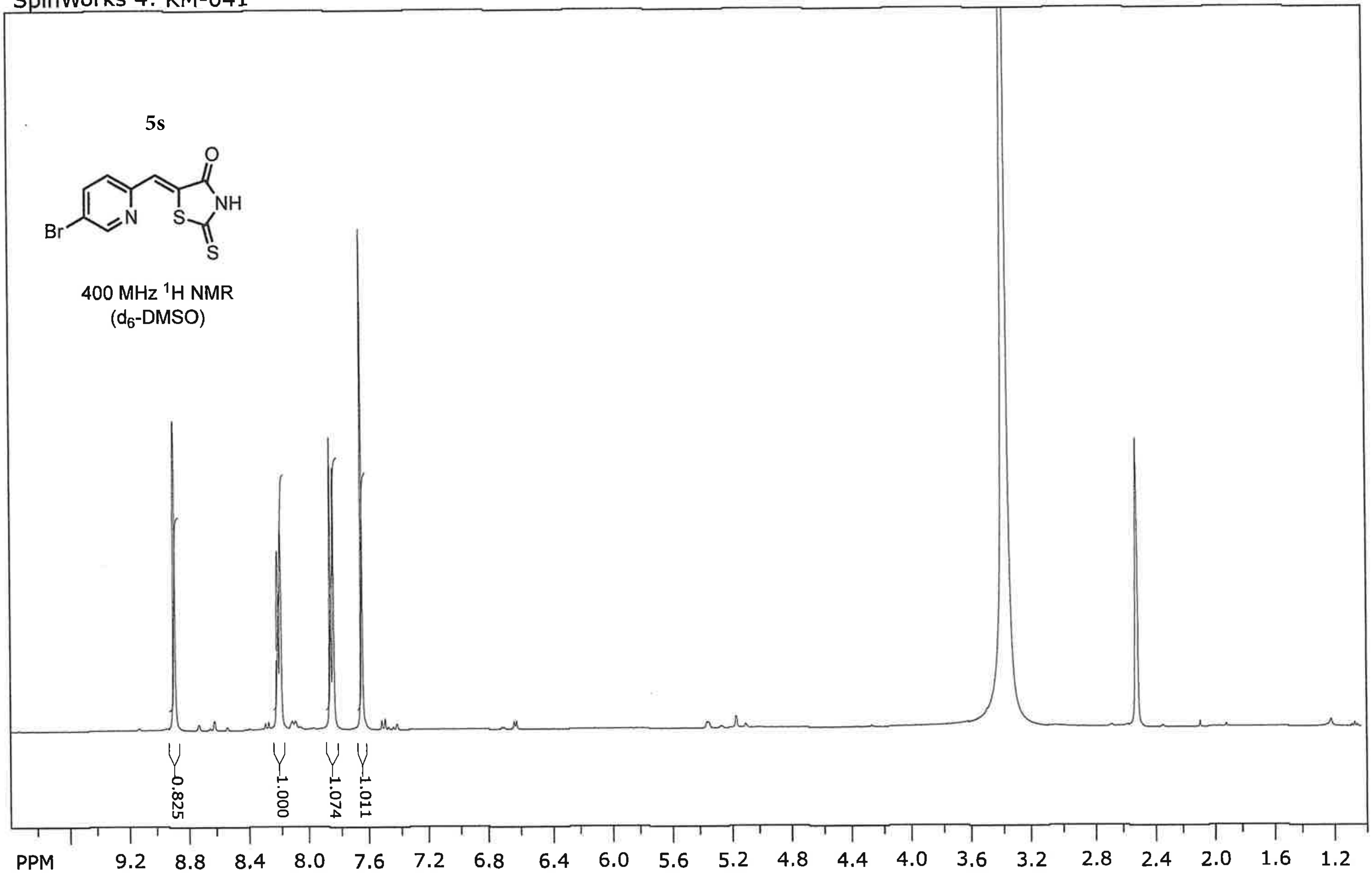

file: ...esktop\KM spectra\KM-048R2.fid\fid block# 1 expt: "s2pul"
transmitter freq.: 399.732139 MHz
time domain size: 32768 points
width: 6410.26 Hz = 16.0364 ppm = 0.195626 Hz/pt
number of scans: 32

freq. of 0 ppm: 399.729736 MHz
processed size: 32768 complex points
LB: 0.500 GF: 0.0000
Hz/cm: 143.875 ppm/cm: 0.35993

SpinWorks 4:

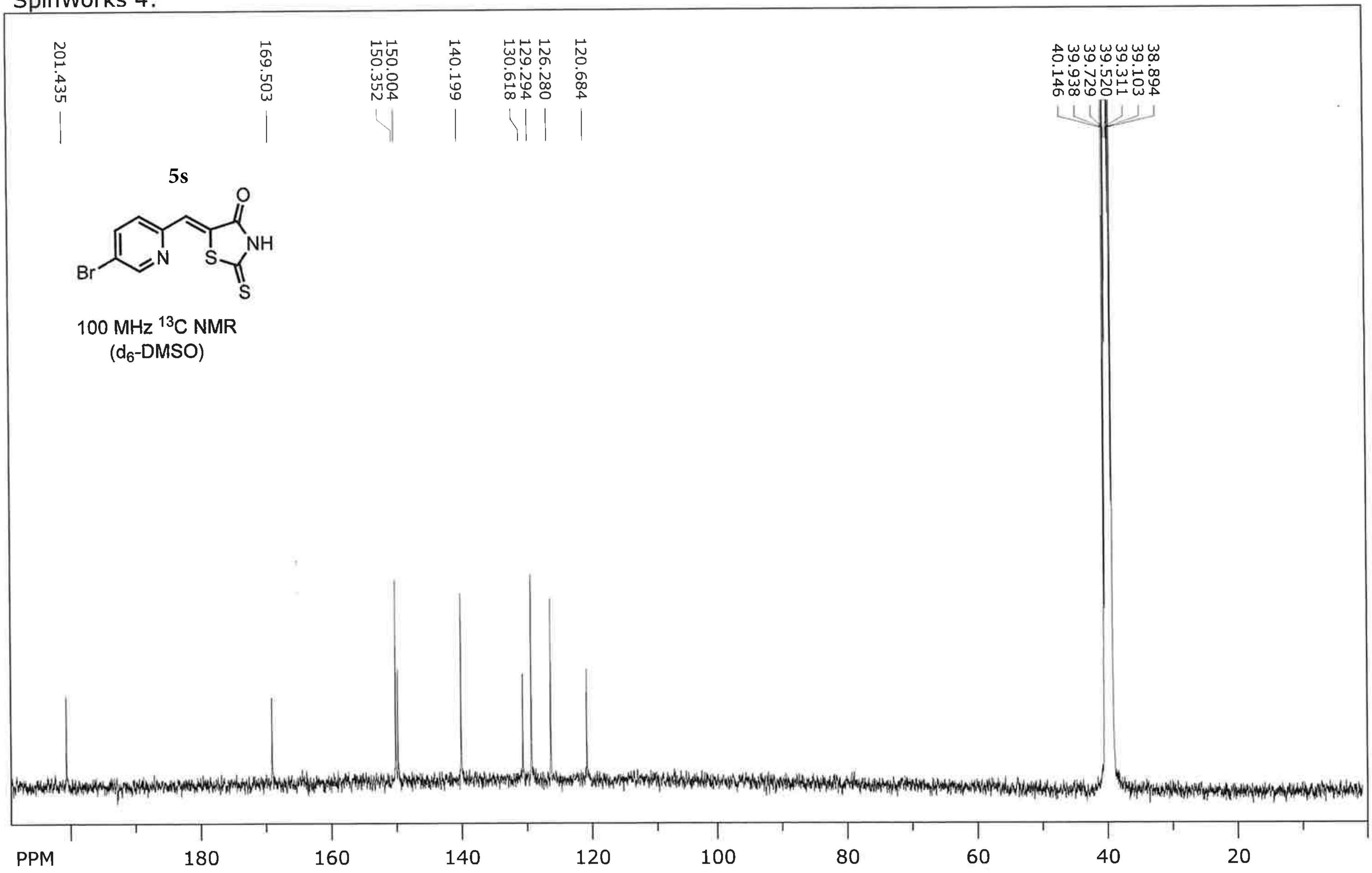

file: ...op\KM048_d6DMSO_2026_08_27.fid\fid block# 1 expt: "s2pul"
transmitter freq.: 100.522325 MHz
time domain size: 50000 points
width: 25000.00 Hz = 248.7010 ppm = 0.500000 Hz/pt
number of scans: 10000

freq. of 0 ppm: 100.511308 MHz
processed size: 65536 complex points
LB: 2.500 GF: 0.0000
Hz/cm: 844.444 ppm/cm: 8.40057

SpinWorks 4: KM-038

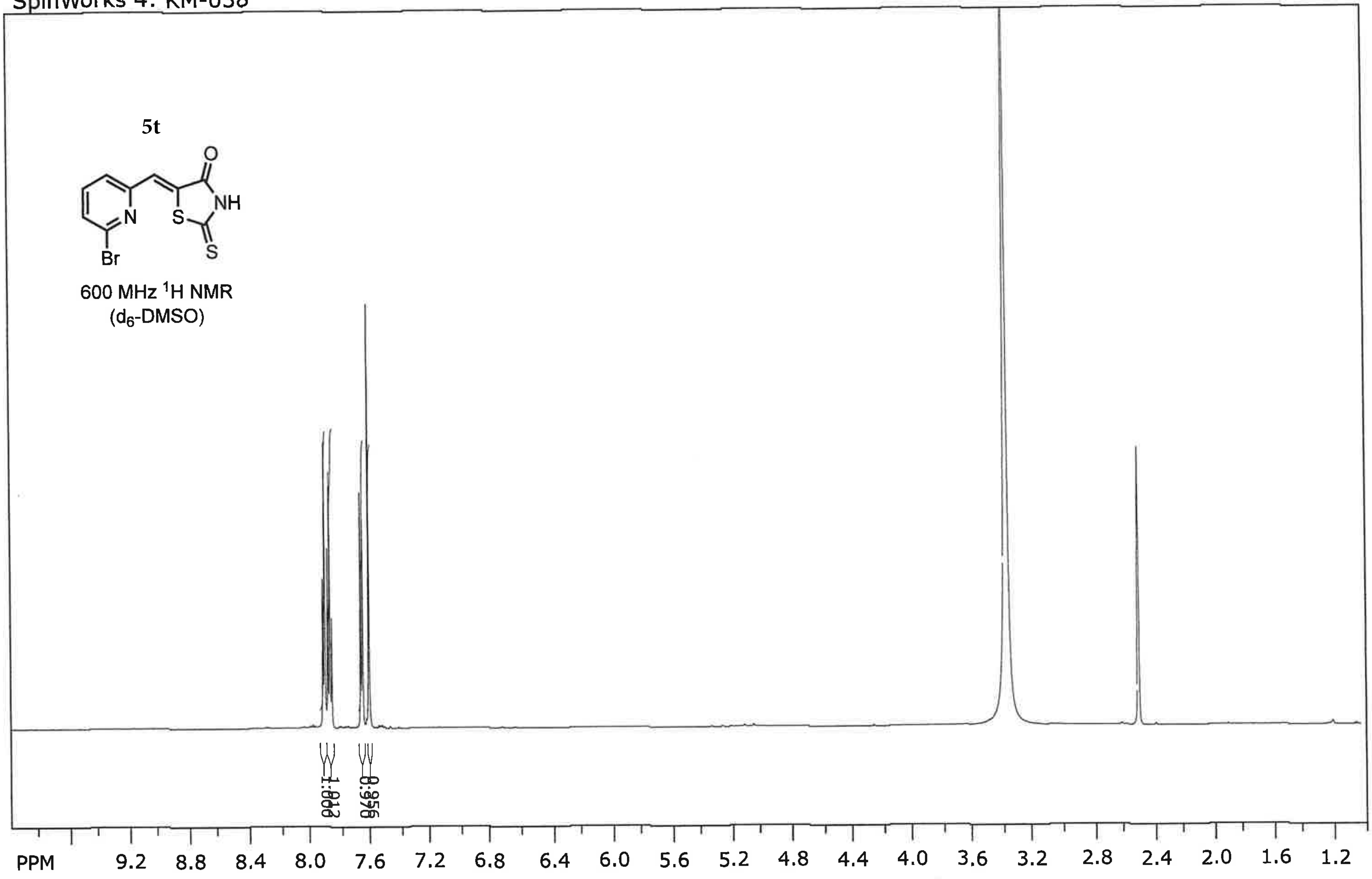

file: ...\Desktop\KM spectra\KM-051.fid\fid block# 1 expt: "s2pul"
transmitter freq.: 599.508636 MHz
time domain size: 32768 points
width: 9615.38 Hz = 16.0388 ppm = 0.293438 Hz/pt
number of scans: 32

freq. of 0 ppm: 599.505038 MHz
processed size: 32768 complex points
LB: 0.500 GF: 0.0000
Hz/cm: 215.812 ppm/cm: 0.35998

SpinWorks 4:

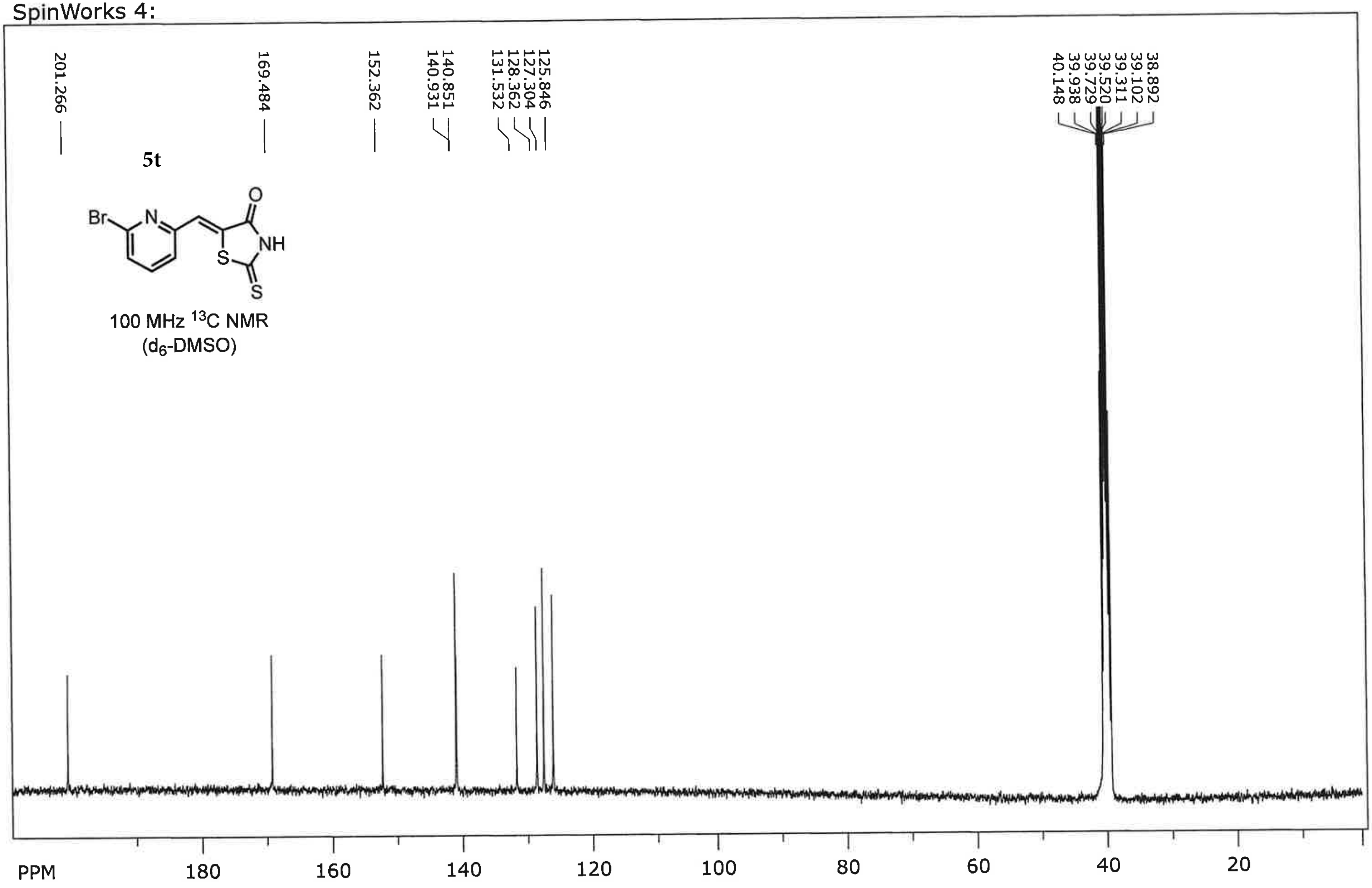

file: ...ectra\KM 050-059\KM-051C13.fid\fid block# 1 expt: "s2pul"
transmitter freq.: 100.522893 MHz
time domain size: 50000 points
width: 25000.00 Hz = 248.6996 ppm = 0.500000 Hz/pt
number of scans: 10000

freq. of 0 ppm: 100.511855 MHz
processed size: 65536 complex points
LB: 2.500 GF: 0.0000
Hz/cm: 844.444 ppm/cm: 8.40052

SpinWorks 4:

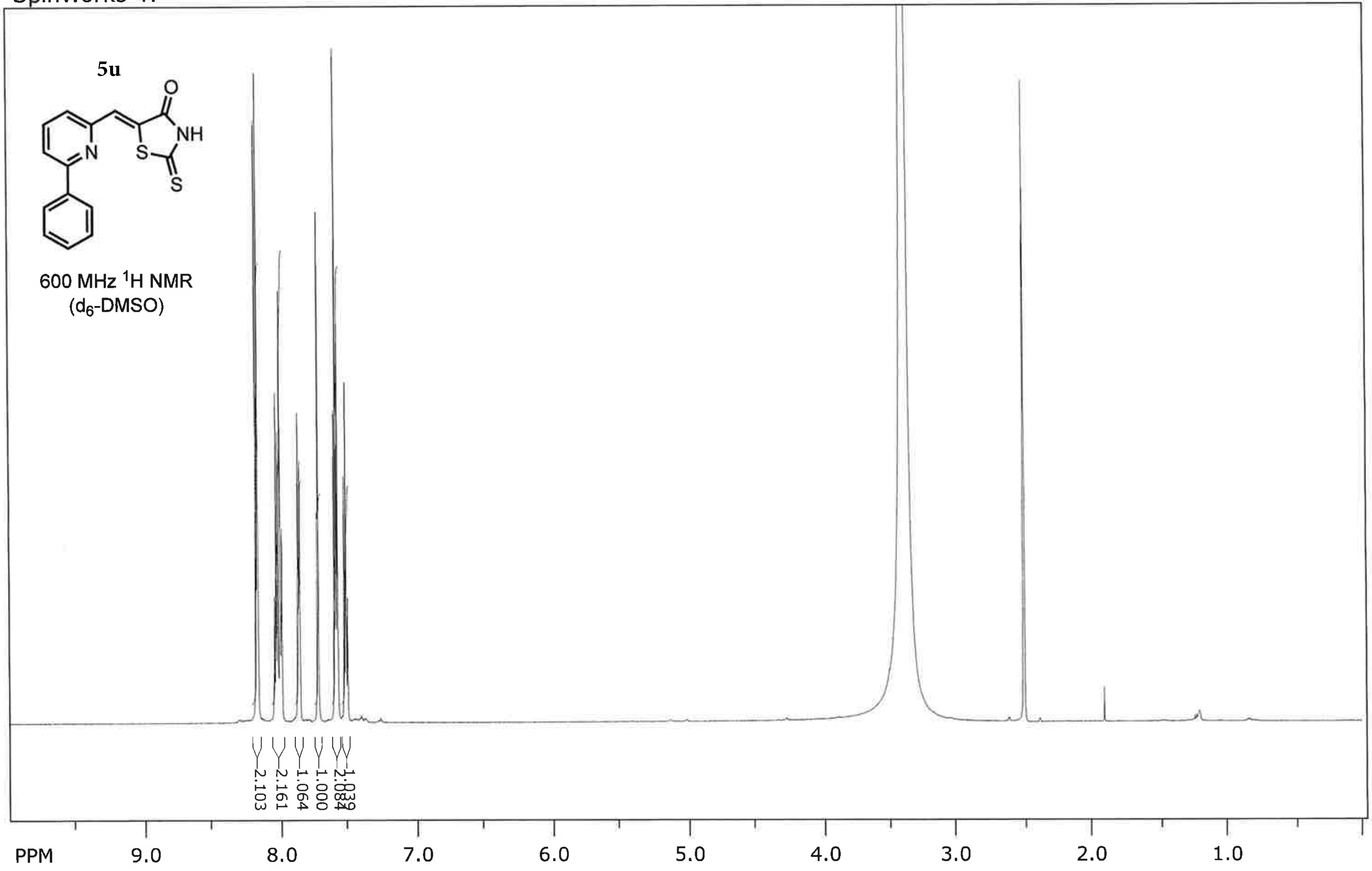

file: ...op\WD404_d6DMSO_2026_05_16.fid\fid block# 1 expt: "s2pul"
transmitter freq.: 599.508651 MHz
time domain size: 32768 points
width: 9615.38 Hz = 16.0388 ppm = 0.293438 Hz/pt
number of scans: 16

freq. of 0 ppm: 599.505041 MHz
processed size: 32768 complex points
LB: 0.500 GF: 0.0000
Hz/cm: 239.957 ppm/cm: 0.40026

SpinWorks 4:

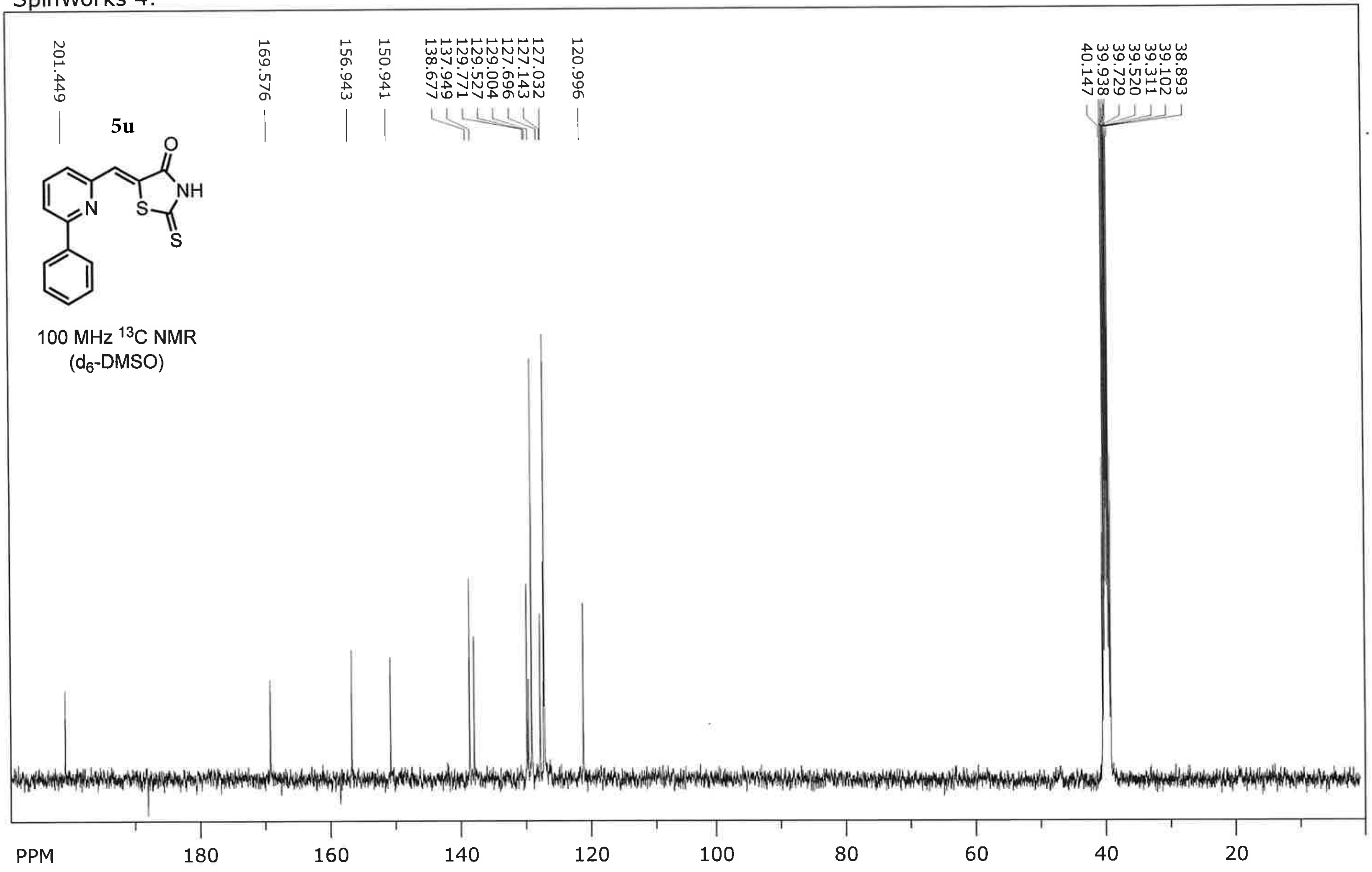

file: ...D404_13C_d6DMSO_2026_05_31.fid\fid block# 1 expt: "s2pul"
transmitter freq.: 100.522537 MHz
time domain size: 50000 points
width: 25000.00 Hz = 248.7004 ppm = 0.500000 Hz/pt
number of scans: 1000

freq. of 0 ppm: 100.511517 MHz
processed size: 65536 complex points
LB: 2.500 GF: 0.0000
Hz/cm: 843.889 ppm/cm: 8.39502

SpinWorks 4: EAW975A

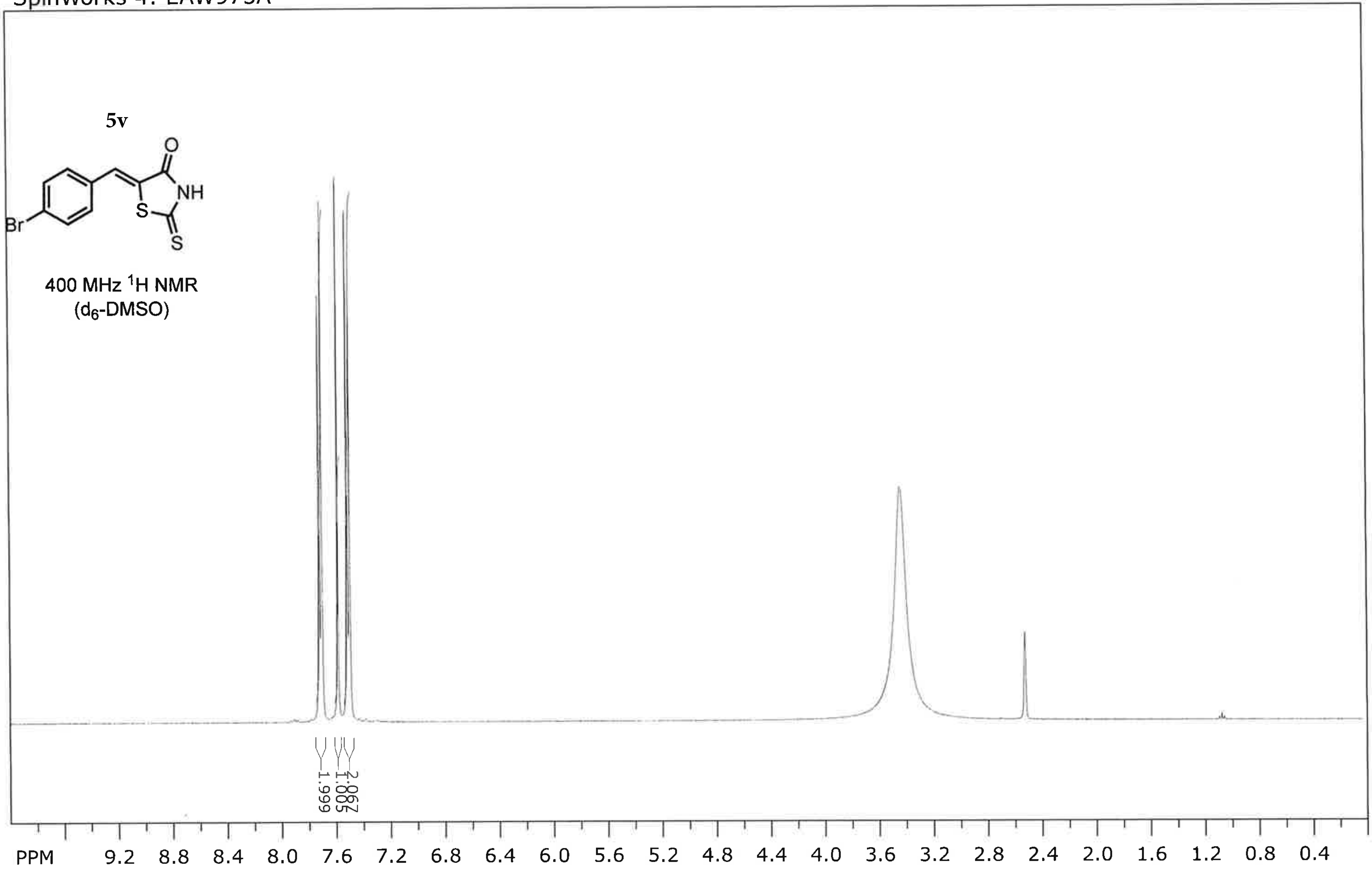

file: ...niversity\Desktop\EAW_975A.fid\fid block# 1 expt: "s2pul"
transmitter freq.: 399.732139 MHz
time domain size: 32768 points
width: 6410.26 Hz = 16.0364 ppm = 0.195626 Hz/pt
number of scans: 8

freq. of 0 ppm: 399.729737 MHz
processed size: 32768 complex points
LB: 0.500 GF: 0.0000
Hz/cm: 159.829 ppm/cm: 0.39984

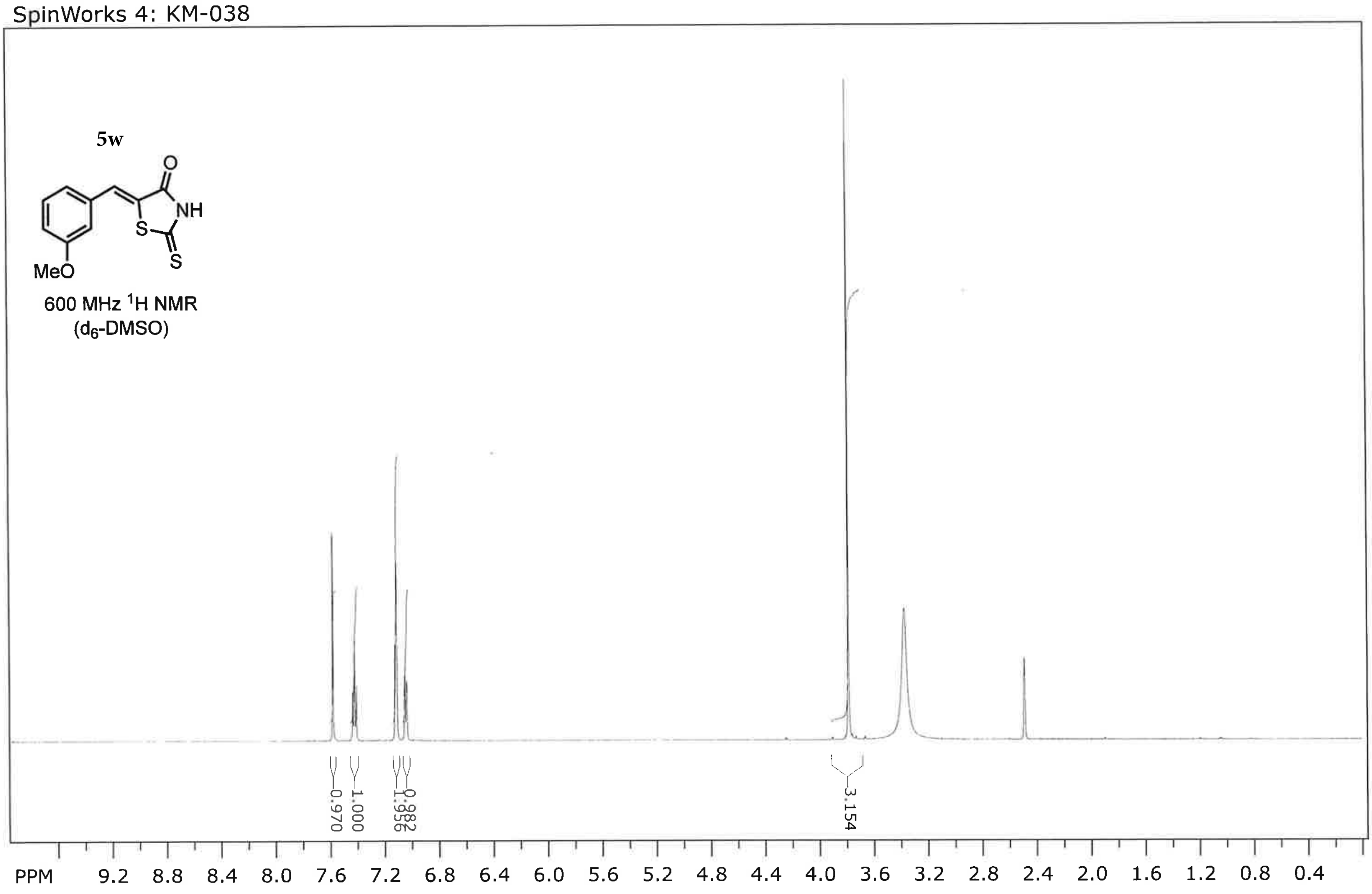

file: ... spectra\KM 050-059\KM-053.fid\fid block# 1 expt: "s2pul"
transmitter freq.: 599.508636 MHz
time domain size: 32768 points
width: 9615.38 Hz = 16.0388 ppm = 0.293438 Hz/pt
number of scans: 32

freq. of 0 ppm: 599.505031 MHz
processed size: 32768 complex points
LB: 0.500 GF: 0.0000
Hz/cm: 239.744 ppm/cm: 0.39990

SpinWorks 4:

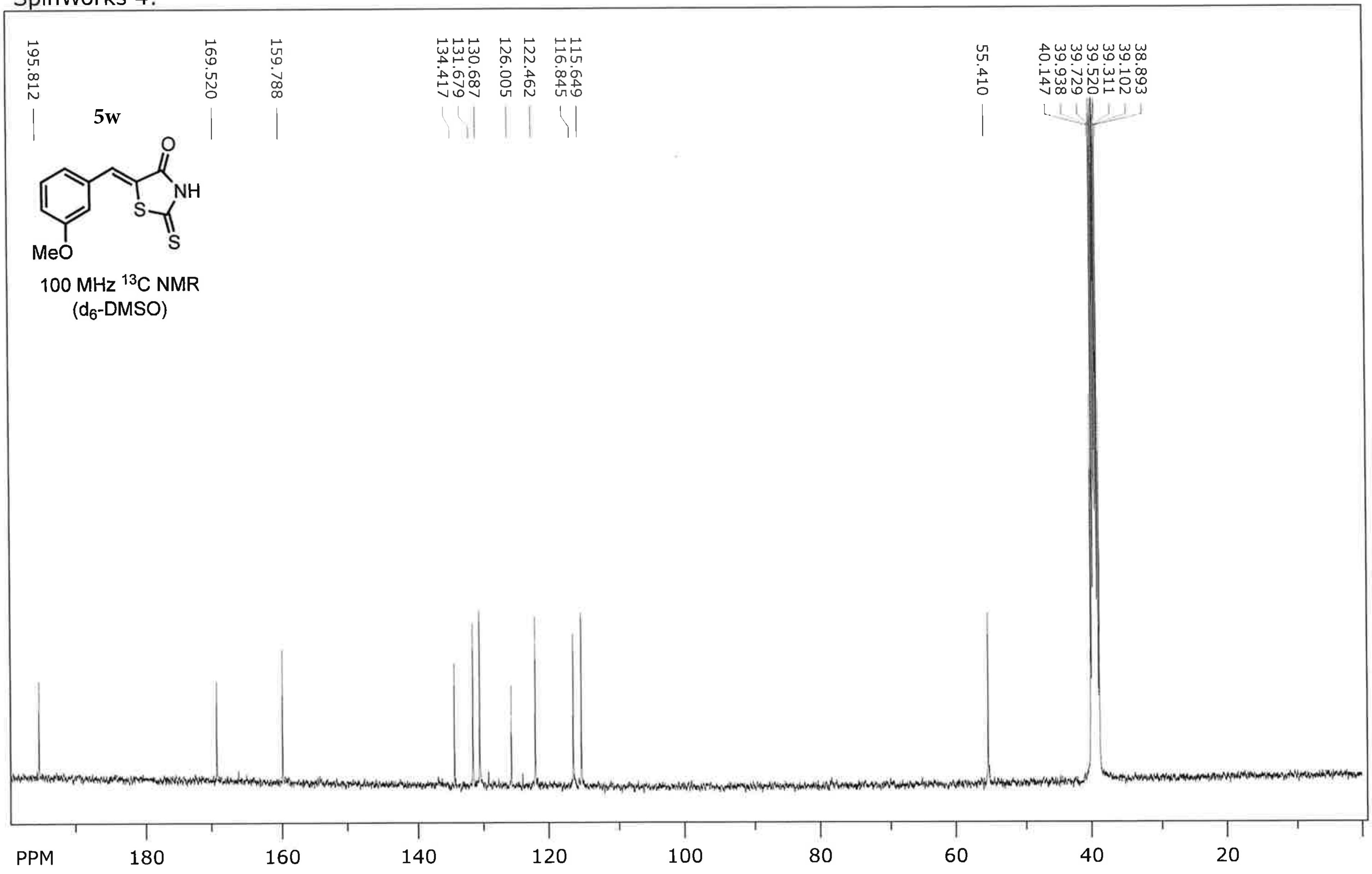

file: ...050-059\KM-053C13_20251120.fid\fid block# 1 expt: "s2pul"
transmitter freq.: 100.522893 MHz
time domain size: 50000 points
width: 25000.00 Hz = 248.6996 ppm = 0.500000 Hz/pt
number of scans: 10000

freq. of 0 ppm: 100.511869 MHz
processed size: 65536 complex points
LB: 2.500 GF: 0.0000
Hz/cm: 804.444 ppm/cm: 8.00260

Generic Display Report

Analysis Info

Analysis Name	D:\Data\Data_20260612\WD-402_ESI_pos_000001.d	Acquisition Date	6/12/2026 10:03:26 AM
Method	ESI_pos_100-500_XR_ODU	Operator	Admin
Sample Name	WD-402	Instrument	solariX XR

Comment: WD-402 in AcN:MeOH (1:1)
Expected Mass of C11H7N3OS2 H+ = 262.010331 m/z
Observed Mass = 262.010281 m/z

Difference < 1.0 ppm

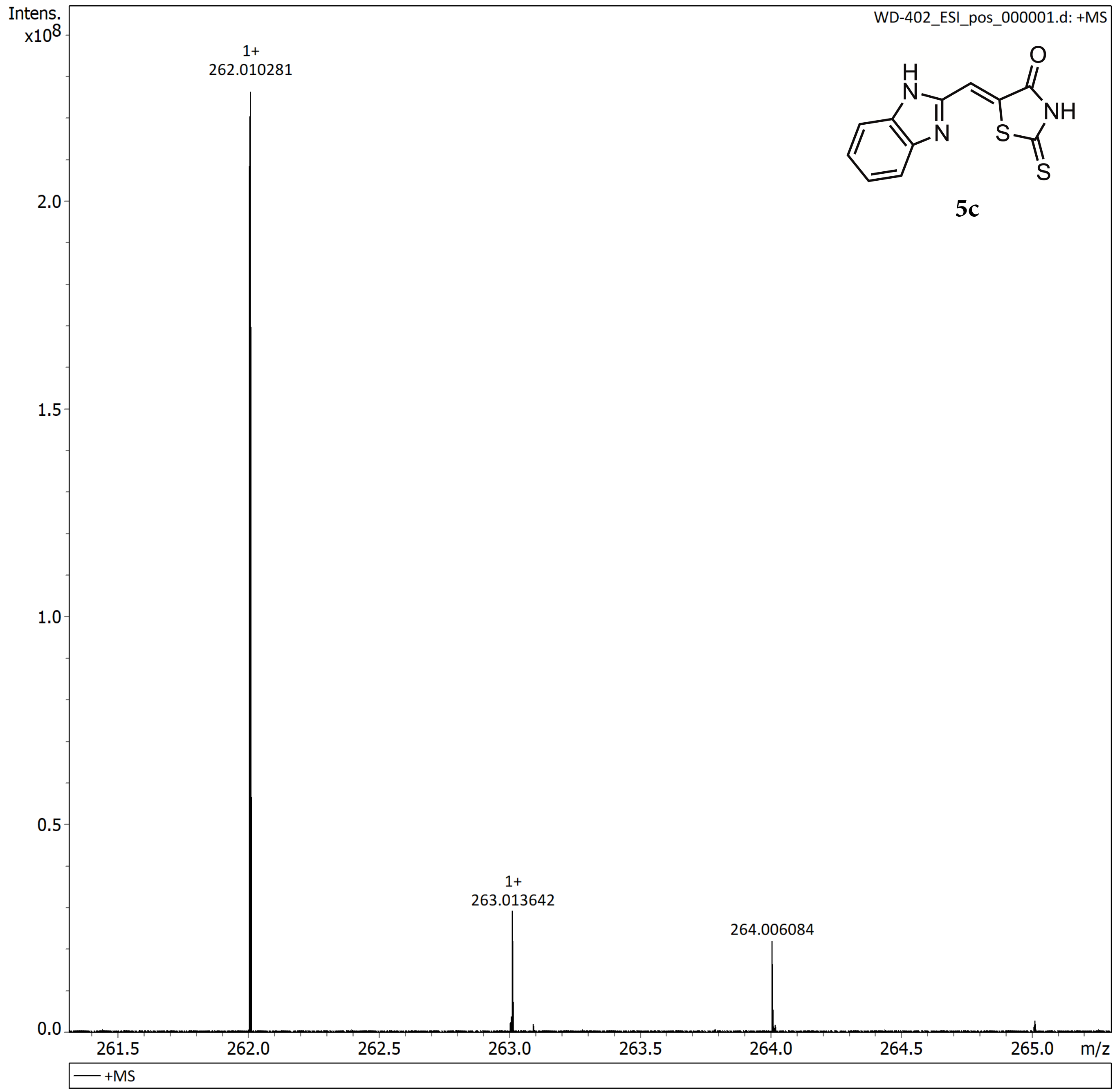

Generic Display Report

Analysis Info

Analysis Name	D:\Data\Data_20260330\WD393_pos_000001.d	Acquisition Date	3/30/2026 3:21:46 PM
Method	LowMass_150-1000_050825	Operator	Admin
Sample Name	WD393_pos	Instrument	solariX XR
Comment	WD393_pos in AcN:MeOH (1:1) C7H5N3S2O H+		

Sample Name WD393_pos in AcN:MeOH (1:1)

Exact Mass of C7H5N3S2O H+ = 211.994681 m/z

Mass Observed = 211.994624 m/z

Difference < 1.0 ppm

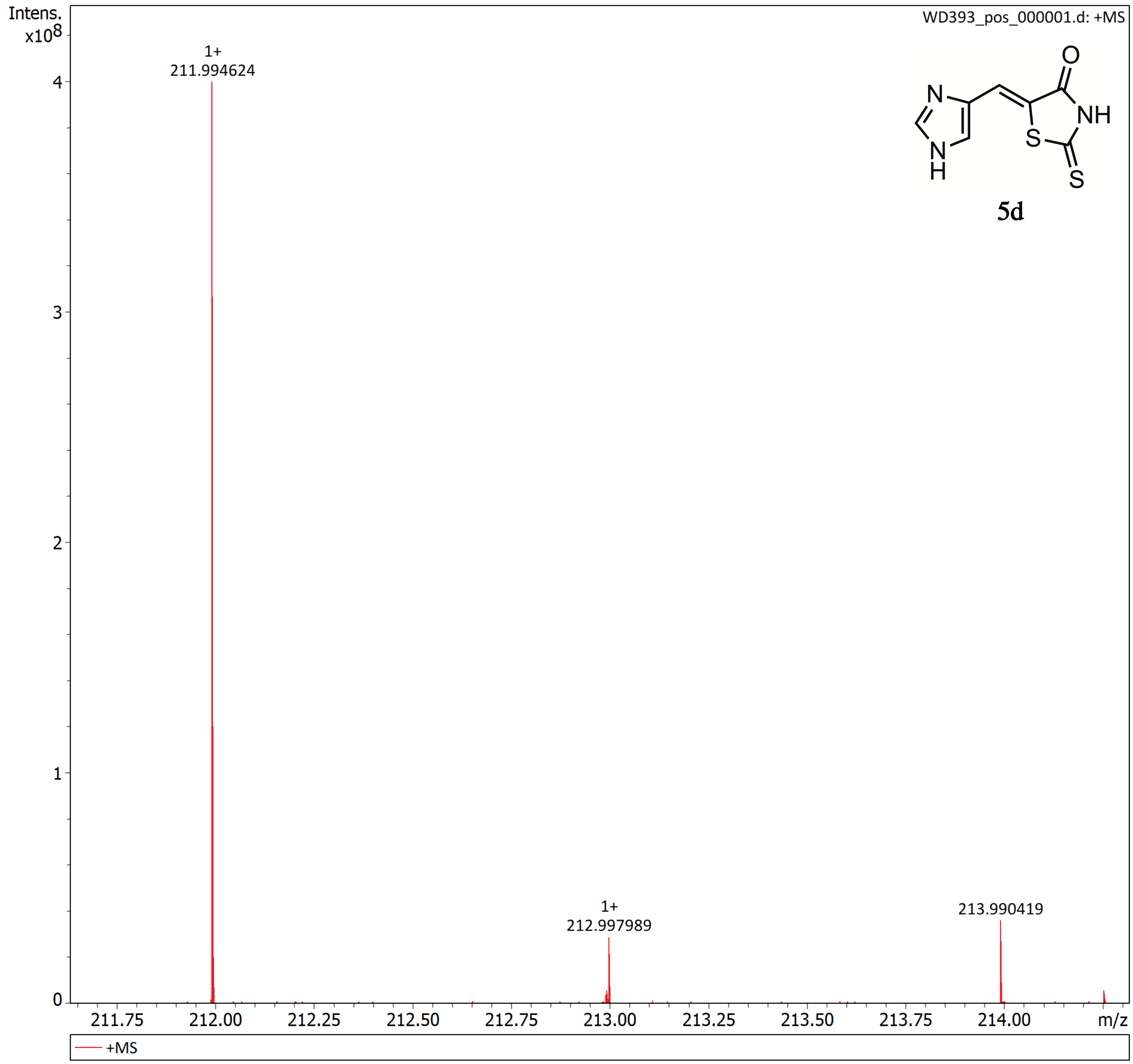

Generic Display Report

Analysis Info

		Acquisition Date	3/30/2026 4:02:28 PM
Analysis Name	D:\Data\Data_20260330\WD394_pos_000001.d		
Method	LowMass_150-1000_050825	Operator	Admin
Sample Name	WD394_pos	Instrument	solariX XR
Comment	WD394_pos in AcN:MeOH (1:1)	C8H7N3S2O H+	

Sample Name WD394_pos in AcN:MeOH (1:1)

Exact Mass of C8H7N3S2O H+ = 226.010331 m/z

Mass Observed = 226.010369 m/z

Difference < 1.0 ppm

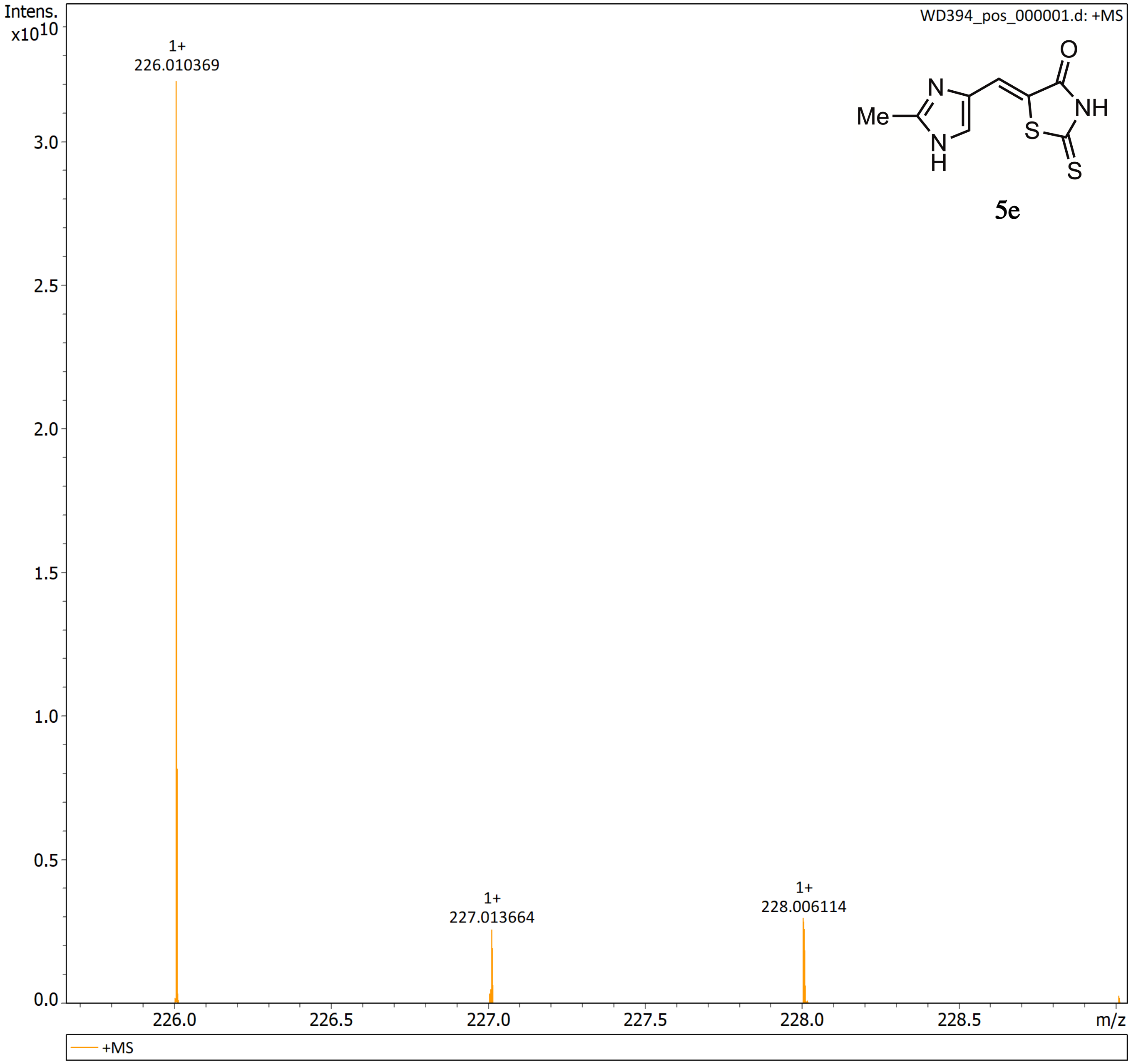

Generic Display Report

Analysis Info

Analysis Name	D:\Data\Data_20260612\WD-405_ESI_pos_000001.d	Acquisition Date	6/12/2026 10:51:26 AM
Method	ESI_pos_100-500_XR_ODU	Operator	Admin
Sample Name	WD-405	Instrument	solariX XR

Comment: WD-405 in AcN:MeOH (1:1)
Expected Mass of C13H9N3OS2 H+ = 288.025981 m/z
Observed Mass = 288.025942 m/z
Difference < 1.0 ppm

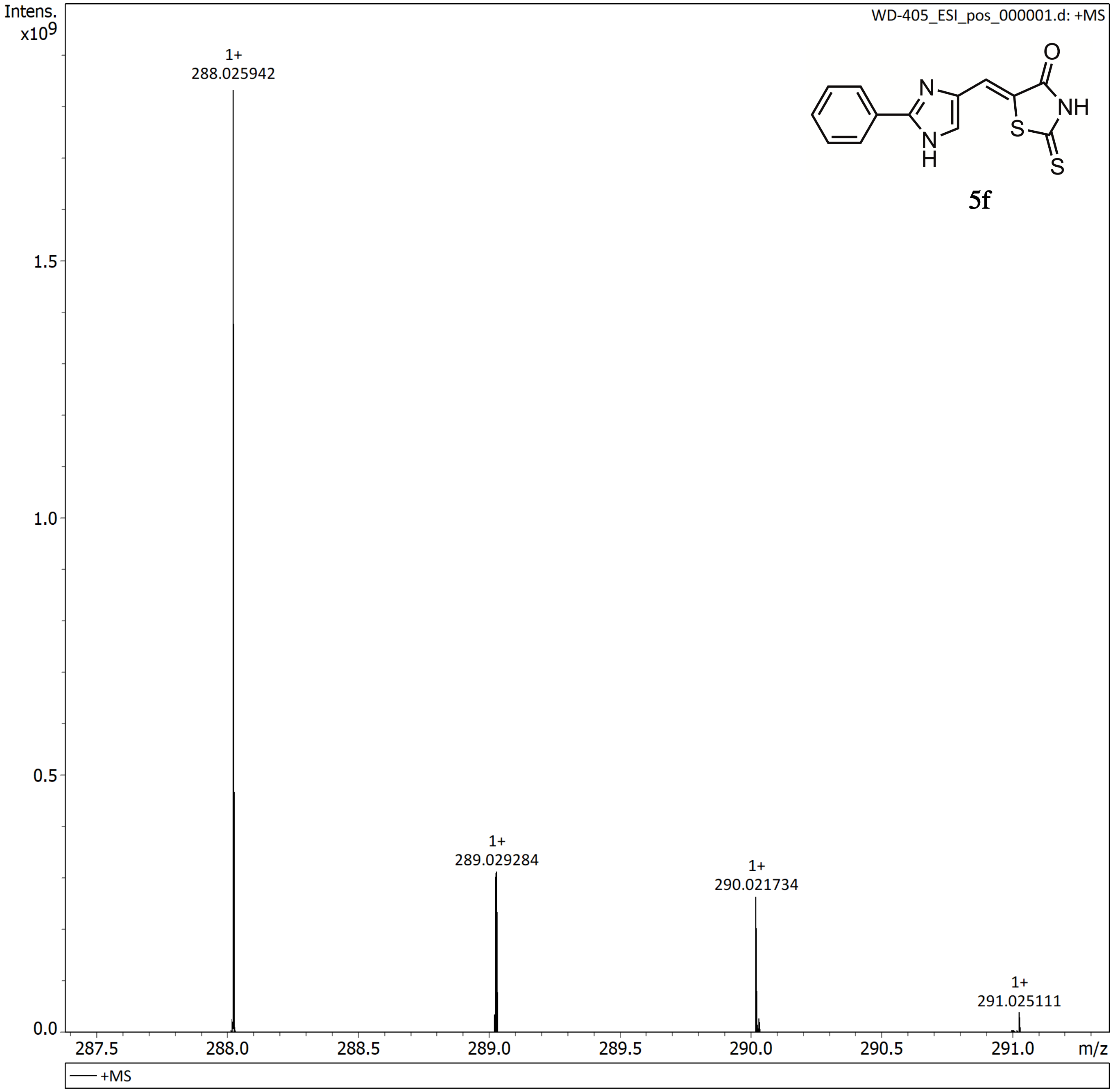

Generic Display Report

Analysis Info

		Acquisition Date	3/30/2026 4:29:33 PM
Analysis Name	D:\Data\Data_20260330\WD398_pos_000001.d		
Method	LowMass_150-1000_050825	Operator	Admin
Sample Name	WD398_pos	Instrument	solariX XR
Comment	WD398_pos in AcN:MeOH (1:1)	C11H6N2S3O H+	

Sample Name WD398_pos in AcN:MeOH (1:1)

Exact Mass of C11H6N2S3O H+ = 278.971503 m/z

Mass Observed = 278.971412 m/z

Difference < 1.0 ppm

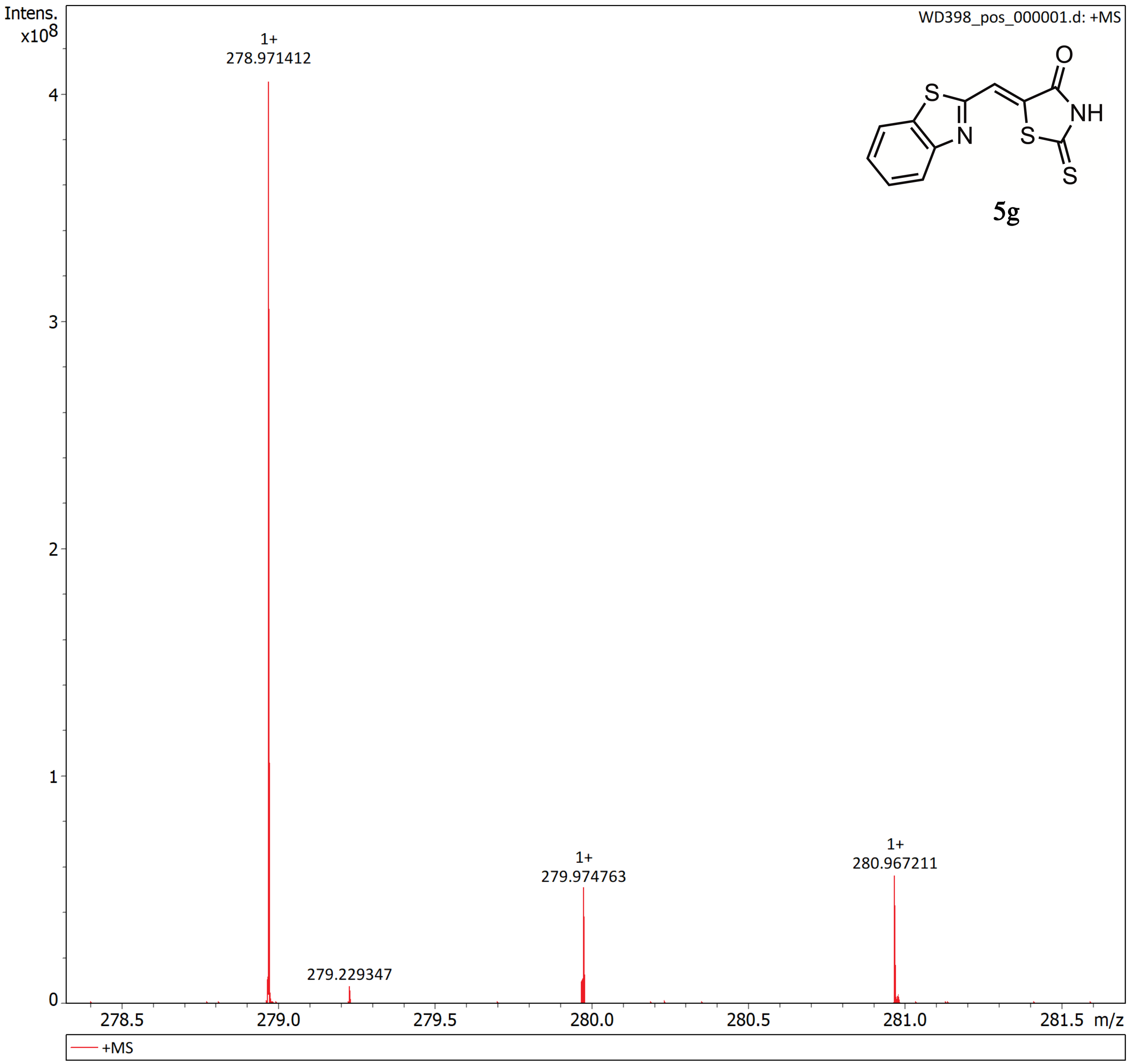

Generic Display Report

Analysis Info

		Acquisition Date	3/30/2026 4:17:47 PM
Analysis Name	D:\Data\Data_20260330\WD396_pos_000001.d		
Method	LowMass_150-1000_050825	Operator	Admin
Sample Name	WD396_pos	Instrument	solariX XR
Comment	WD396_pos in AcN:MeOH (1:1) C13H8N2S3O H+		

Sample Name WD396_pos in AcN:MeOH (1:1)

Exact Mass of C13H8N2S3O H+ = 304.987153 m/z

Mass Observed = 304.987043 m/z

Difference < 1.0 ppm

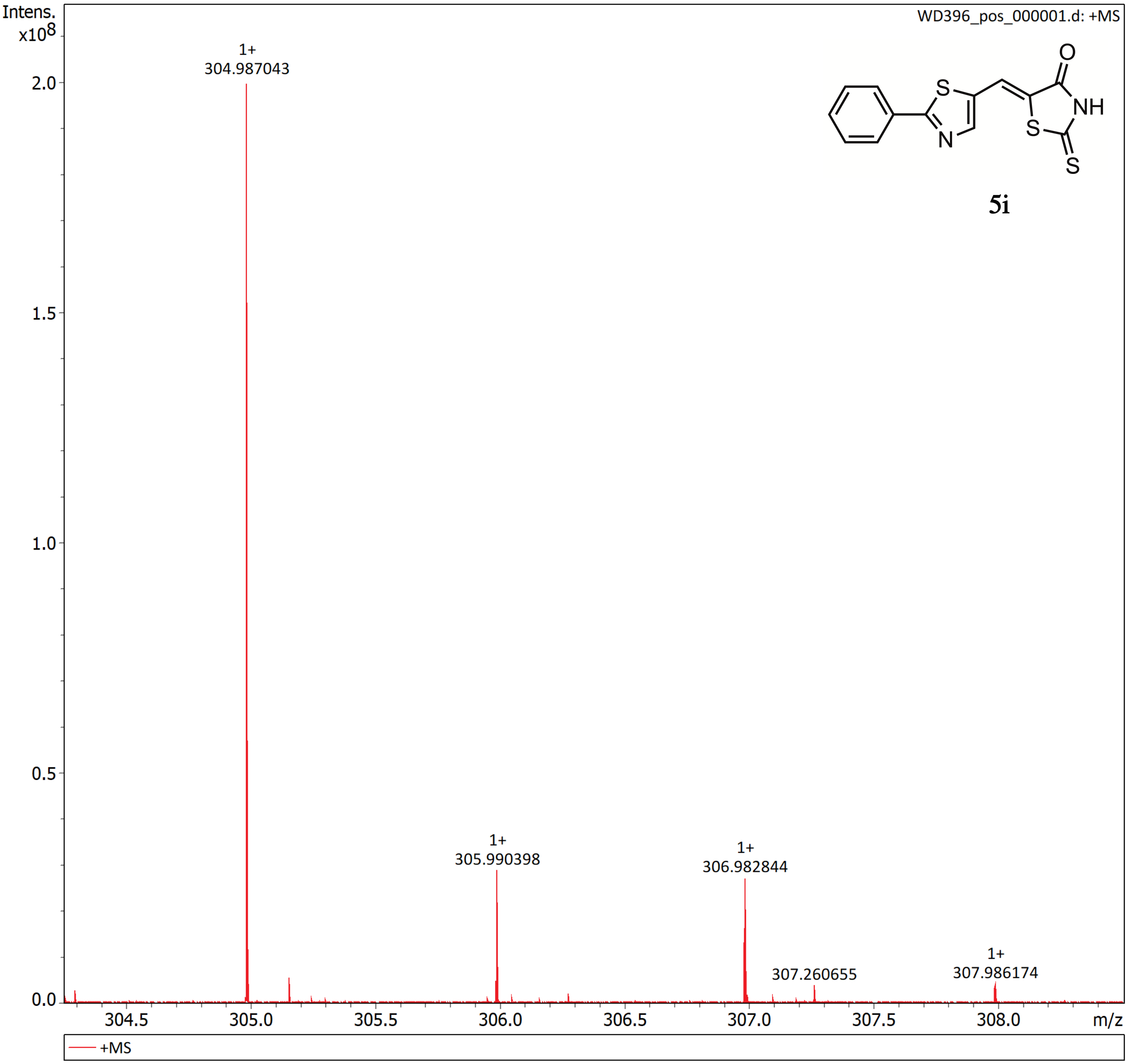

Generic Display Report

Analysis Info

		Acquisition Date	3/30/2026 5:34:07 PM
Analysis Name	D:\Data\Data_20260330\KM064_pos_000001.d		
Method	LowMass_150-1000_050825	Operator	Admin
Sample Name	KM064_pos	Instrument	solariX XR
Comment	KM064_pos in AcN:MeOH (1:1) C10H8N2S2O Na+		

Sample Name	KM064_pos in AcN:MeOH (1:1)			
Exact Mass of	C10H8N2S2O Na+	=	258.997026	m/z
Mass Observed		=	258.996952	m/z

Difference < 1.0 ppm

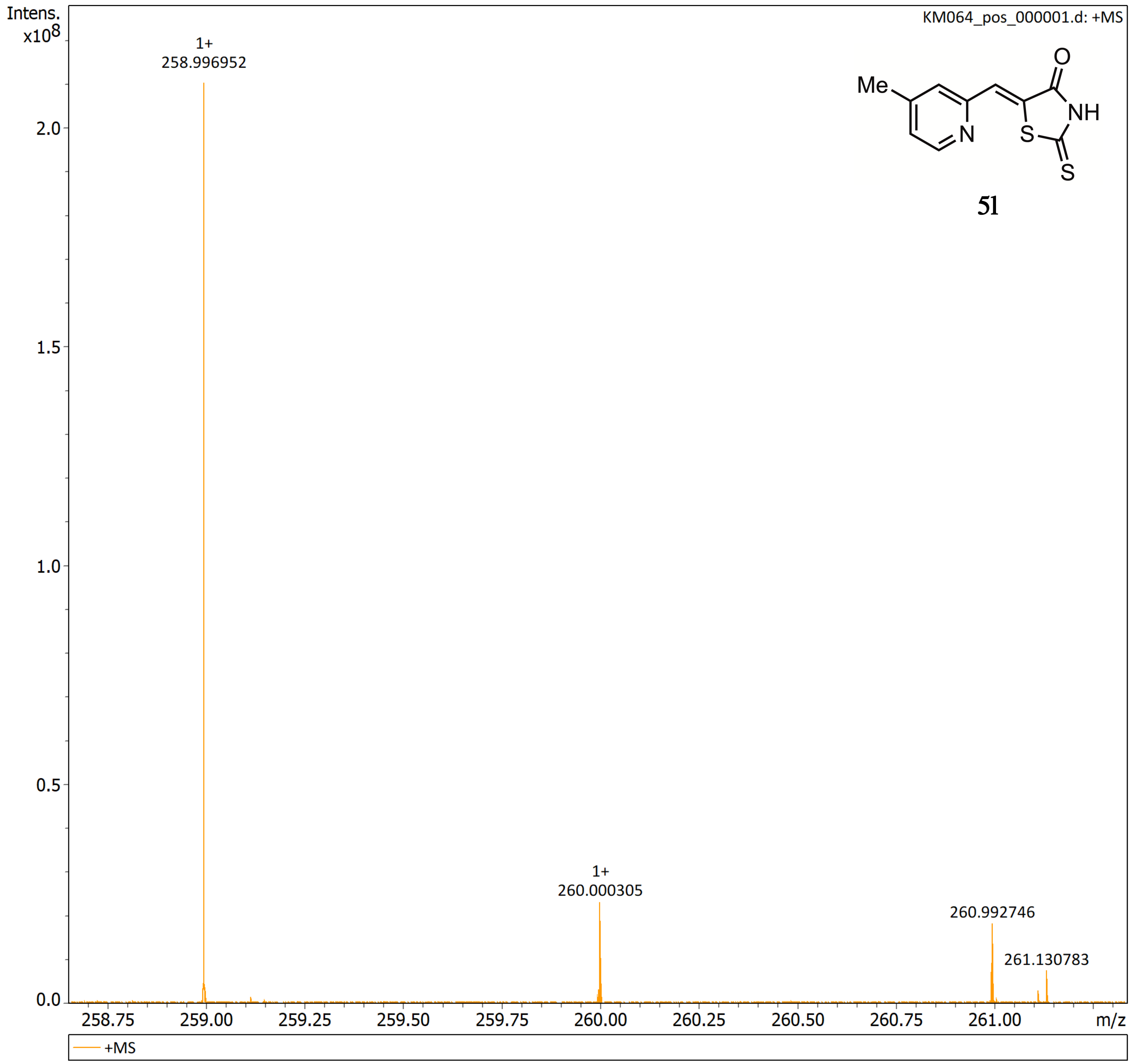

Generic Display Report

Analysis Info

Analysis Name	D:\Data\Data_20260330\KM052_pos_000001.d	Acquisition Date	3/30/2026 5:19:51 PM
Method	LowMass_150-1000_050825	Operator	Admin
Sample Name	KM052_pos	Instrument	solariX XR
Comment	KM052_pos in AcN:MeOH (1:1) C10H8N2S2O Na+		

Sample Name KM052_pos in AcN:MeOH (1:1)

Exact Mass of C10H8N2S2O Na+ = 258.997026 m/z

Mass Observed = 258.996947 m/z

Difference < 1.0 ppm

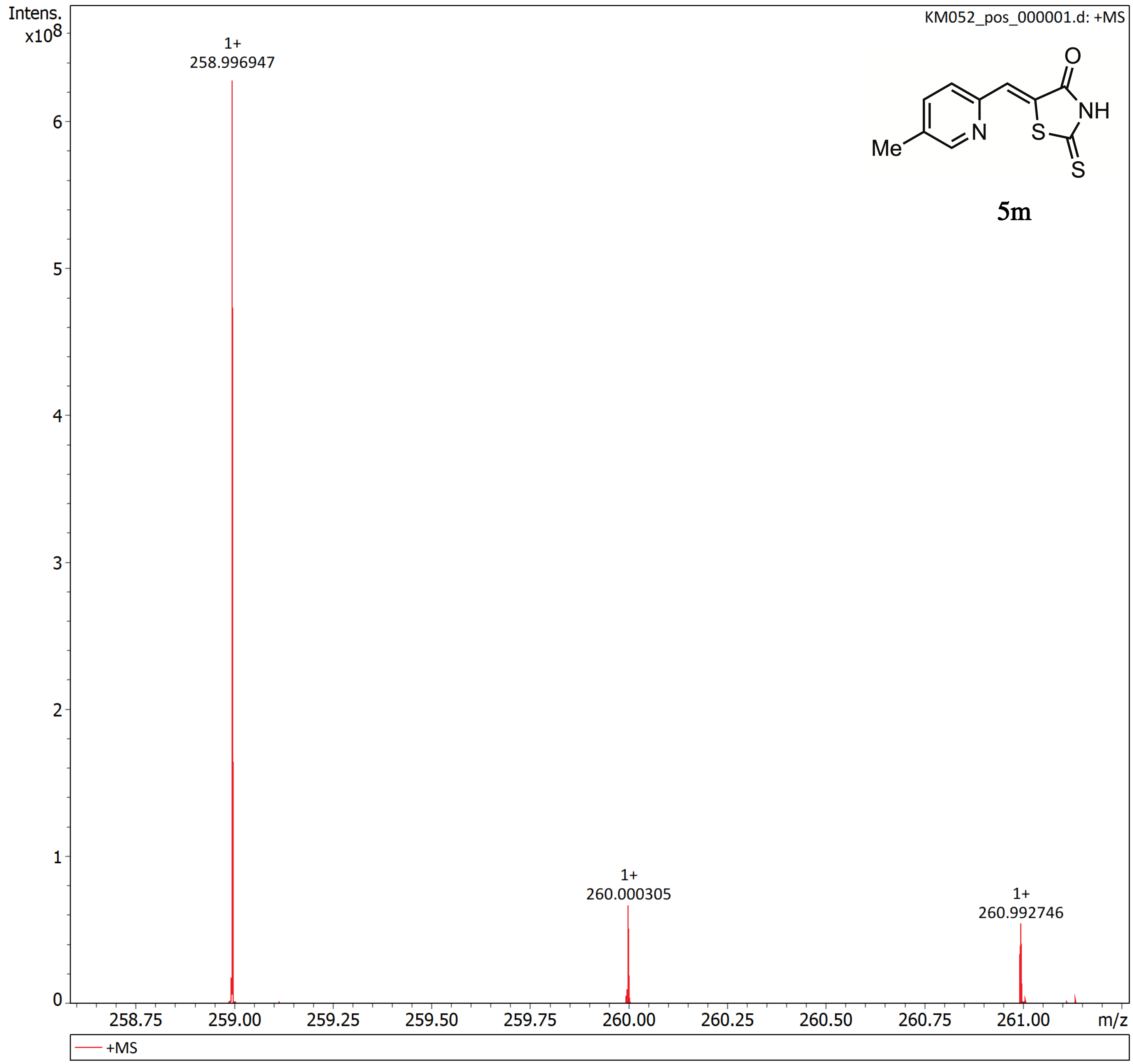

Generic Display Report

Analysis Info

Analysis Name	D:\Data\Data_20260330\KM065_pos_000001.d	Acquisition Date	3/30/2026 5:40:38 PM
Method	LowMass_150-1000_050825	Operator	Admin
Sample Name	KM065_pos	Instrument	solariX XR
Comment	KM065_pos in AcN:MeOH (1:1) C10H8N2S2O Na+		

Sample Name KM065_pos in AcN:MeOH (1:1)

Exact Mass of C10H8N2S2O Na+ = 258.997026 m/z

Mass Observed = 258.996941 m/z

Difference < 1.0 ppm

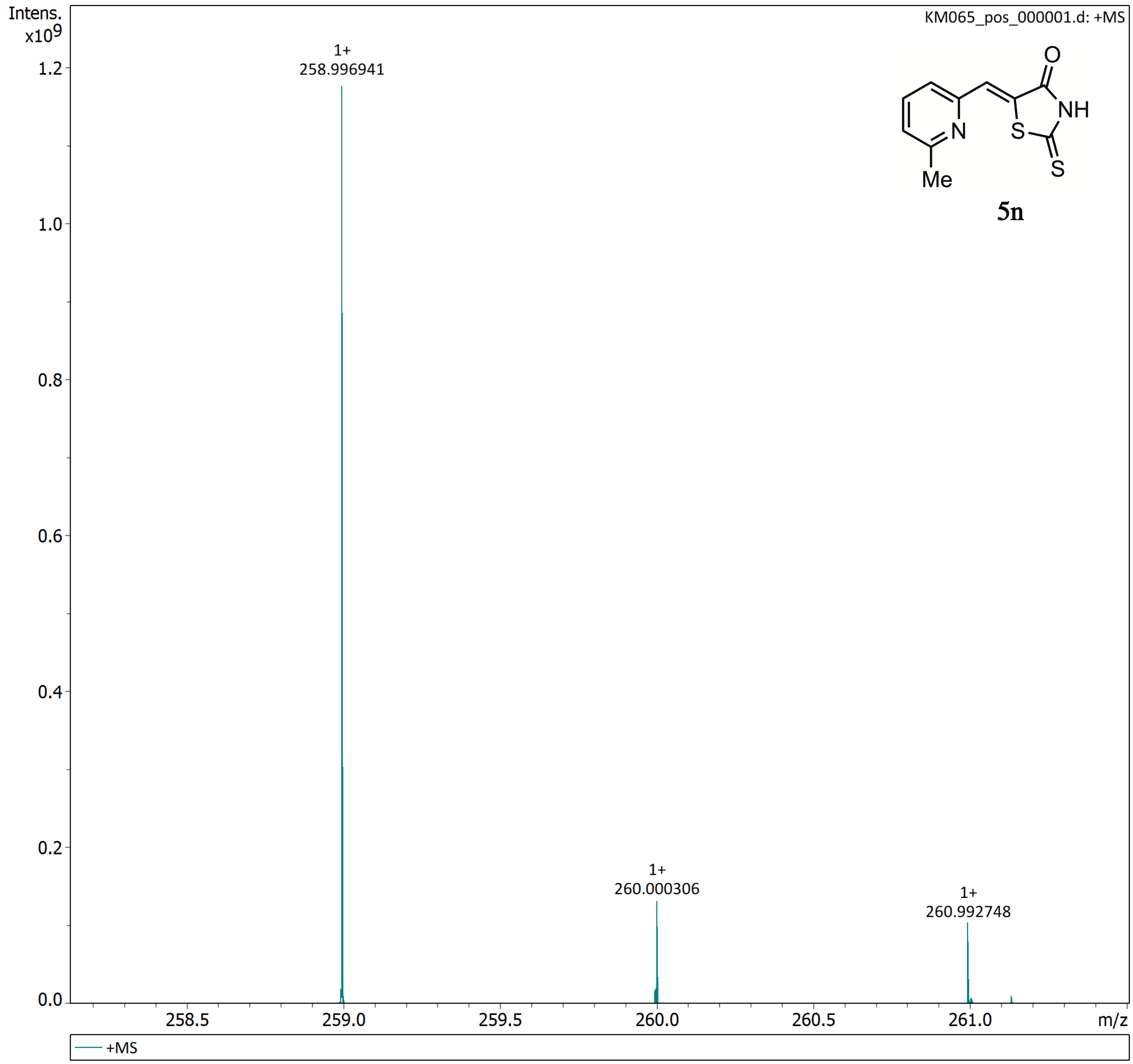

Generic Display Report

Analysis Info

Analysis Name	D:\Data\Data_20260612\WD-400_ESI_pos_000001.d	Acquisition Date	6/12/2026 9:54:31 AM
Method	ESI_pos_100-500_XR_ODU	Operator	Admin
Sample Name	WD-400	Instrument	solariX XR

Comment: WD-400 in AcN:MeOH (1:1)
Expected Mass of C10H8N2O2S2 H+ = 253.009996 m/z
Observed Mass = 253.009970 m/z
Difference < 1.0 ppm

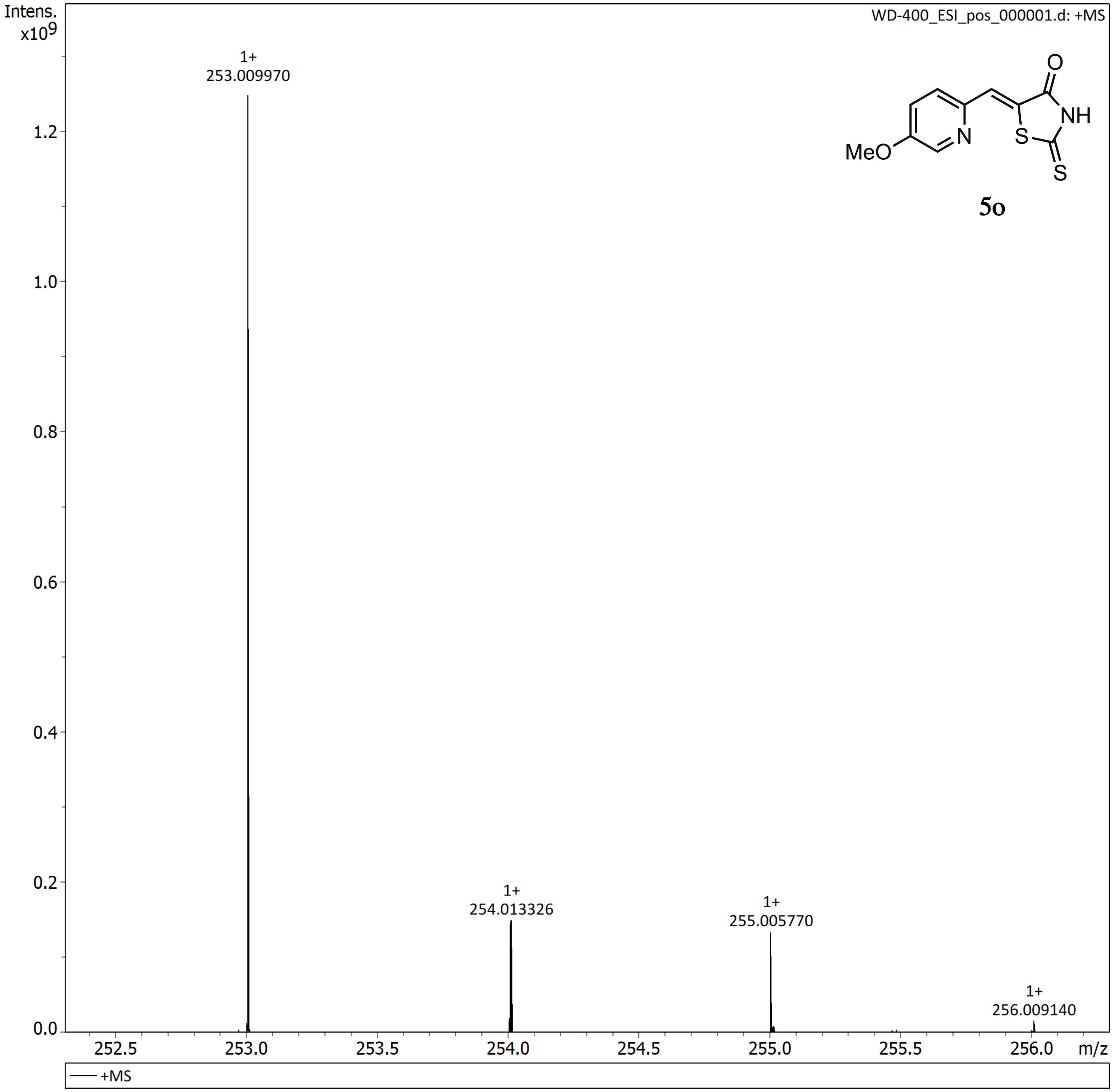

Generic Display Report

Analysis Info

		Acquisition Date	3/30/2026 5:27:25 PM
Analysis Name	D:\Data\Data_20260330\KM063_pos_000001.d		
Method	LowMass_150-1000_050825	Operator	Admin
Sample Name	KM063_pos	Instrument	solariX XR
Comment	KM063_pos in AcN:MeOH (1:1) C10H8N2S2O2 Na+		

Sample Name KM063_pos in AcN:MeOH (1:1)

Exact Mass of C10H8N2S2O2 Na+ = 274.991941 m/z

Mass Observed = 274.991832 m/z

Difference < 1.0 ppm

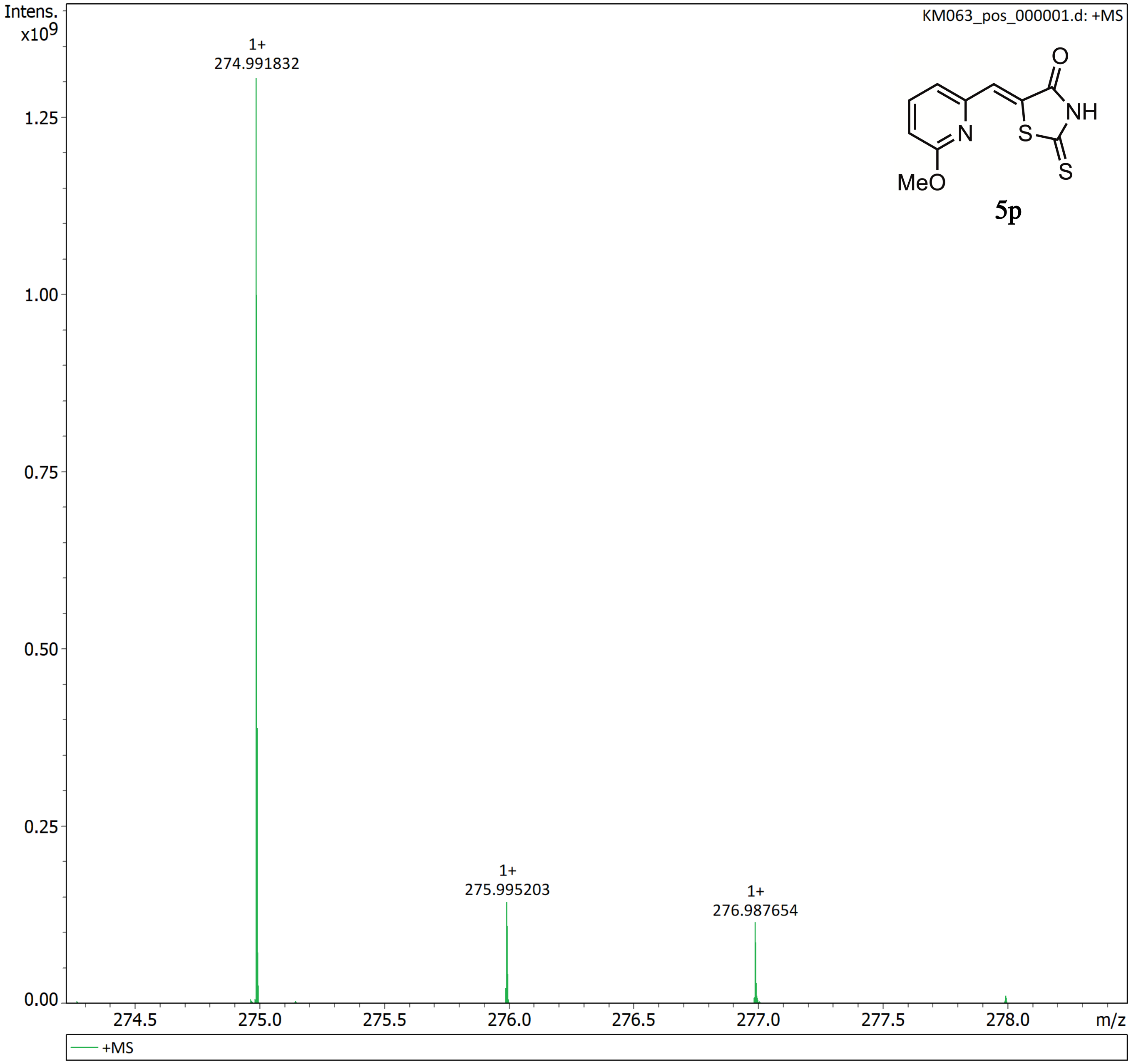

5p

Generic Display Report

Analysis Info

Analysis Name	D:\Data\Data_20260612\WD-401_ESI_pos_000001.d	Acquisition Date	6/12/2026 10:30:06 AM
Method	ESI_pos_100-500_XR_ODU	Operator	Admin
Sample Name	WD-401	Instrument	solariX XR

Comment: WD-401 in AcN:MeOH (1:1)
Expected Mass of C10H8N2O2S2 H+ = 253.009996 m/z
Observed Mass = 253.009945 m/z
Difference < 1.0 ppm

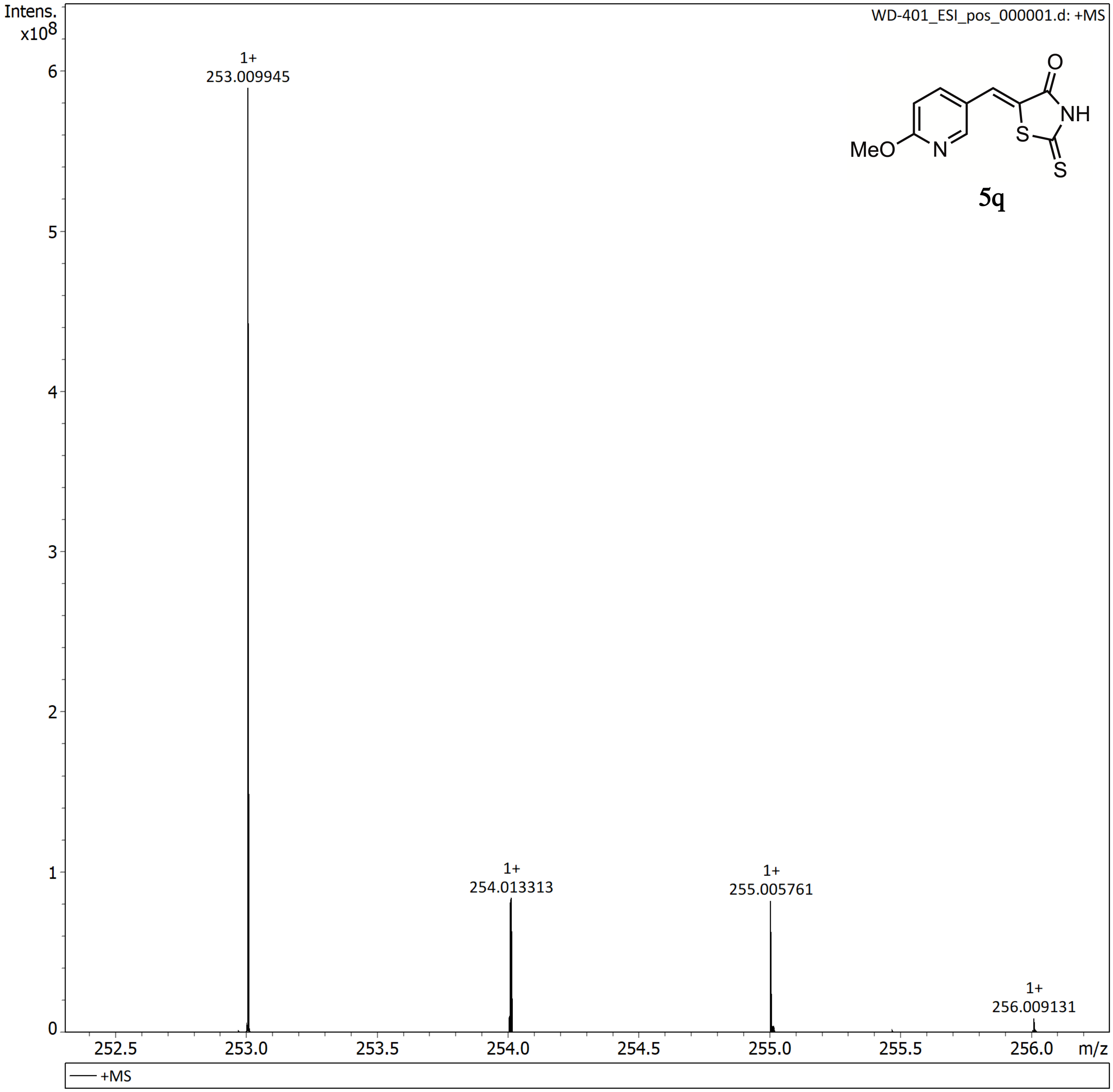

Generic Display Report

Analysis Info

Analysis Name	D:\Data\Data_20260330\KM068_pos_000001.d	Acquisition Date	3/30/2026 5:50:00 PM
Method	LowMass_150-1000_050825	Operator	Admin
Sample Name	KM068_pos	Instrument	solariX XR
Comment	KM068_pos in AcN:MeOH (1:1) C9H5N2S2OBr H+		

Sample Name KM068_pos in AcN:MeOH (1:1)

Exact Mass of C9H5N2S2OBr H+ = 300.909944 m/z

Mass Observed = 300.909839 m/z

Difference < 1.0 ppm

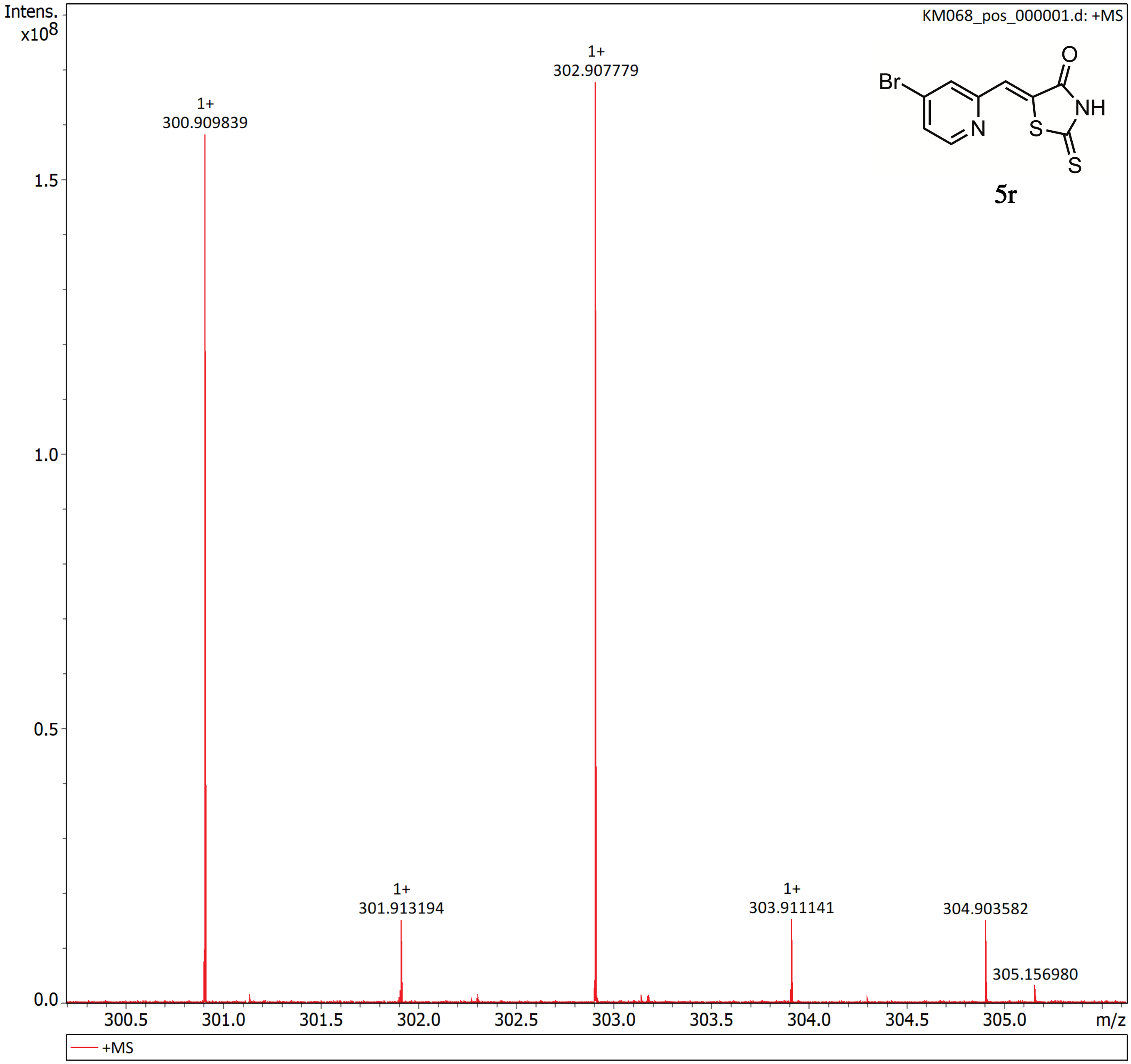

Generic Display Report

Analysis Info

Analysis Name	D:\Data\Data_20260330\KM048_pos_000001.d	Acquisition Date	3/30/2026 4:55:29 PM
Method	LowMass_150-1000_050825	Operator	Admin
Sample Name	KM048_pos	Instrument	solariX XR
Comment	KM048_pos in AcN:MeOH (1:1) C9H5N2S2OBr Na+		

Sample Name KM048_pos in AcN:MeOH (1:1)

Exact Mass of C9H5N2S2OBr Na+ = 322.891888 m/z

Mass Observed = 322.891759 m/z

Difference < 1.0 ppm

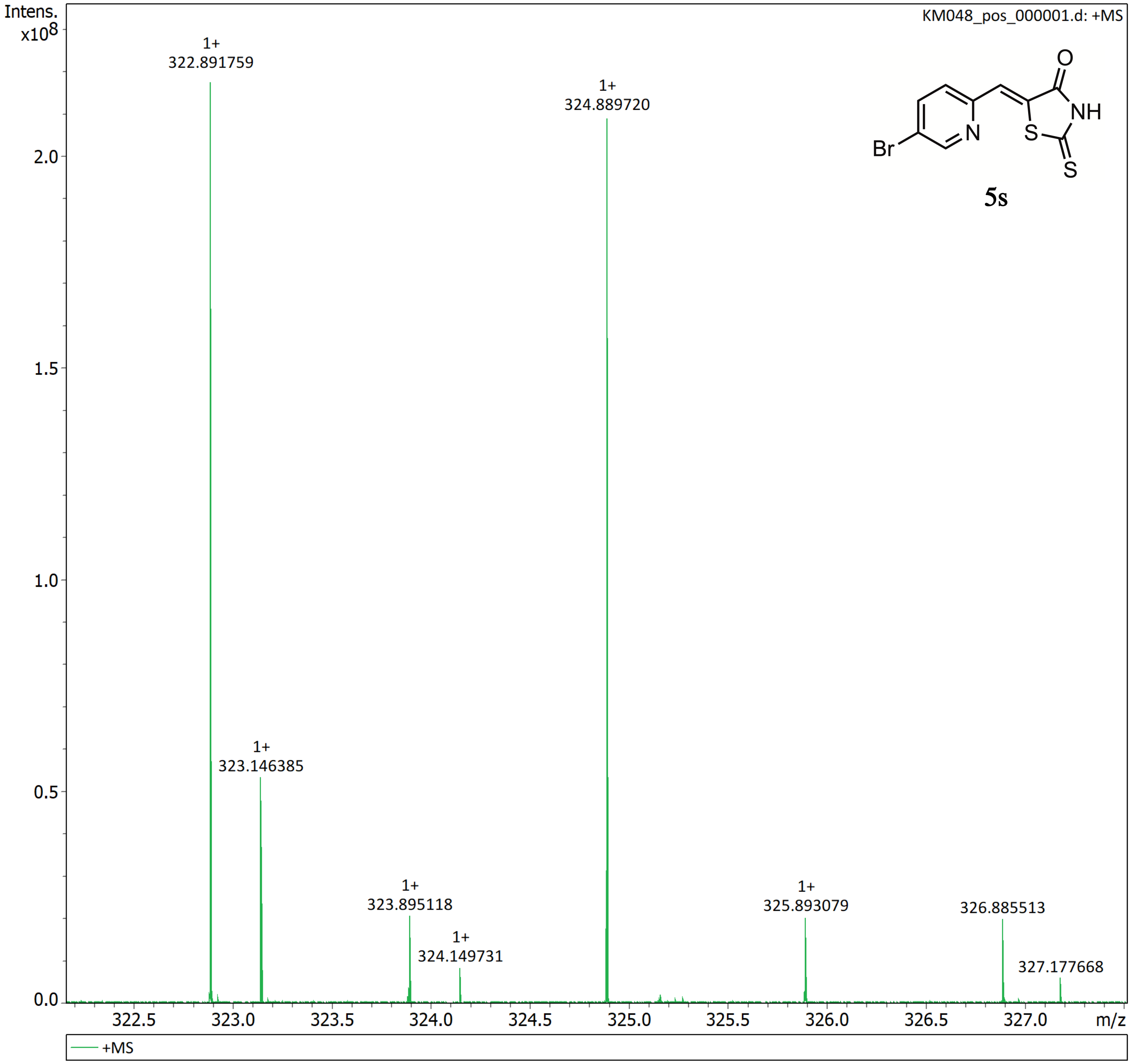

Generic Display Report

Analysis Info

Analysis Name	D:\Data\Data_20260330\KM051_pos_000001.d	Acquisition Date	3/30/2026 5:13:55 PM
Method	LowMass_150-1000_050825	Operator	Admin
Sample Name	KM051_pos	Instrument	solariX XR
Comment	KM051_pos in AcN:MeOH (1:1) C9H5N2S2OBr Na+		

Sample Name KM051_pos in AcN:MeOH (1:1)

Exact Mass of C9H5N2S2OBr Na+ = 322.891888 m/z

Mass Observed = 322.891755 m/z

Difference < 1.0 ppm

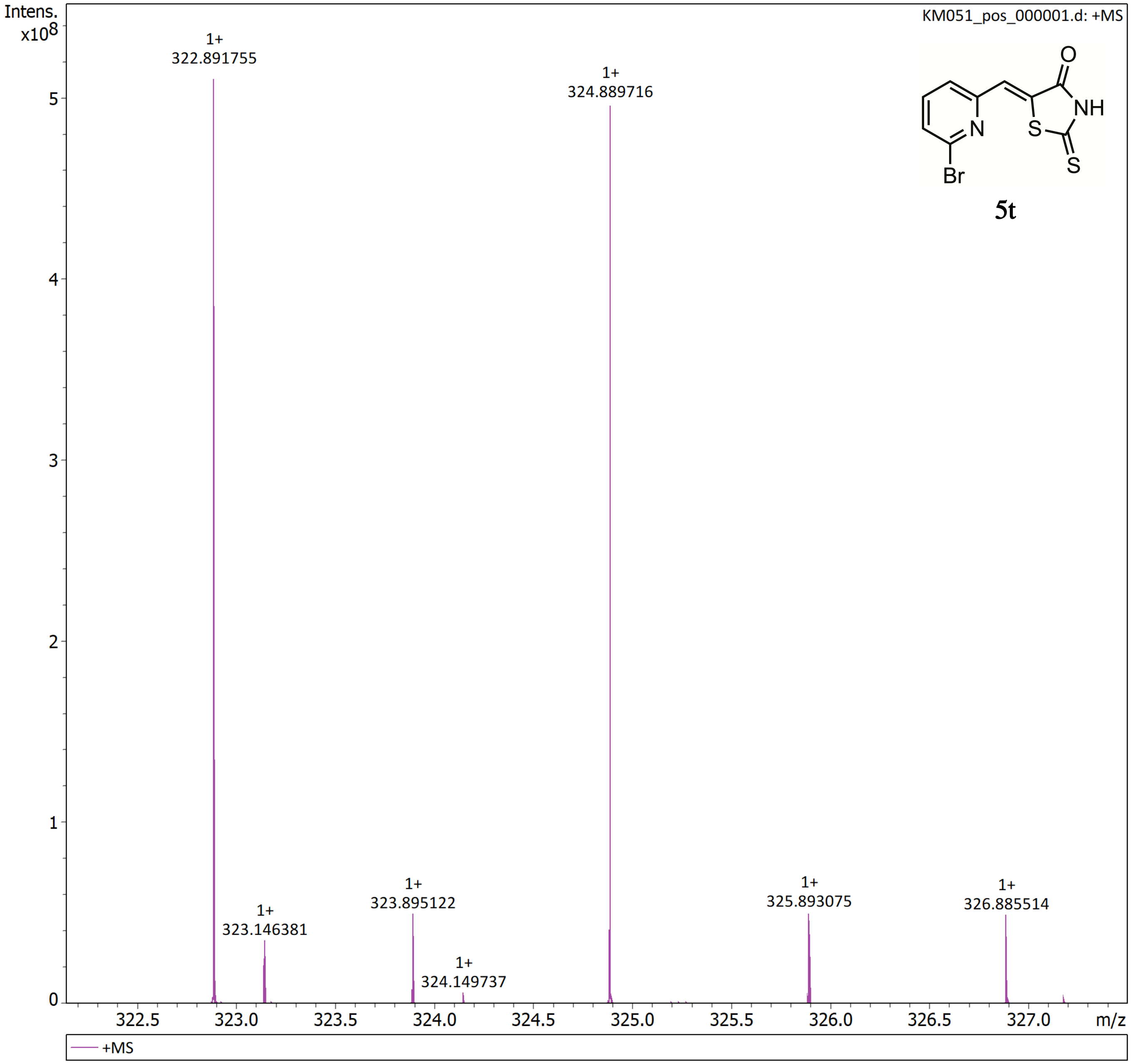

Generic Display Report

Analysis Info

Analysis Name	D:\Data\Data_20260612\WD-404_ESI_pos_000001.d	Acquisition Date	6/12/2026 10:45:10 AM
Method	ESI_pos_100-500_XR_ODU	Operator	Admin
Sample Name	WD-401	Instrument	solariX XR

Comment: WD-404 in AcN:MeOH (1:1)
Expected Mass of C15H10N2OS2 H+ = 299.030732 m/z
Observed Mass = 299.030685 m/z
Difference < 1.0 ppm

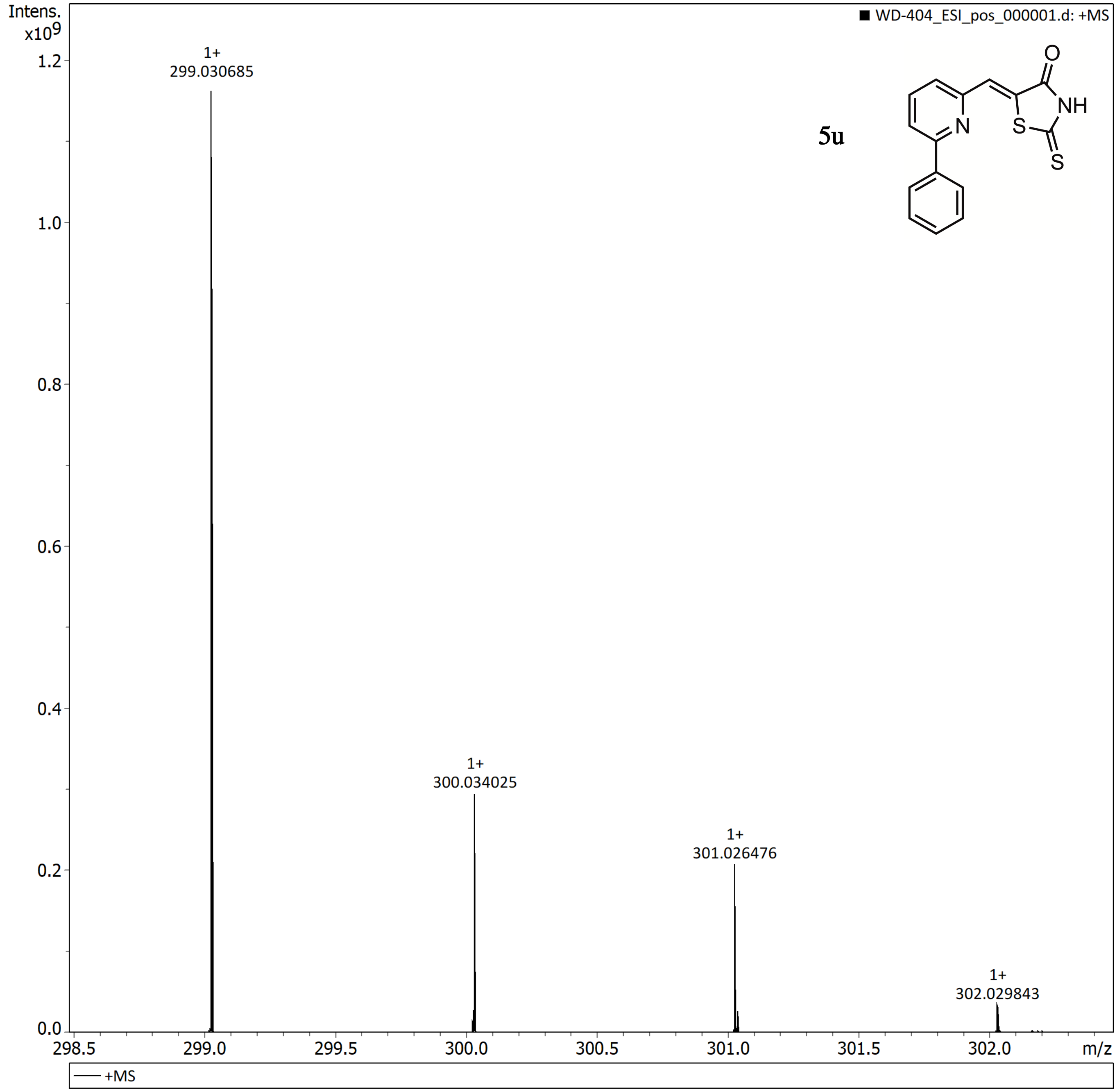

Supplement: Supplementary file 1 [file molecules-31-03413-s001.zip › molecules-4502949-supplementary.pdf]
